# Accessing Perfluoroaryl Sulfonimidamides and Sulfoximines via Photogenerated Perfluoroaryl Nitrenes: Synthesis and Application as a Chiral Auxiliary

**DOI:** 10.1021/acs.joc.1c02241

**Published:** 2021-11-12

**Authors:** Giampiero Proietti, Julius Kuzmin, Azamat Z. Temerdashev, Peter Dinér

**Affiliations:** † Division of Organic Chemistry, Department of Chemistry, KTH-Royal Institute of Technology, Teknikringen 30, 10044 Stockholm, Sweden; ‡ Department of Analytical Chemistry, Kuban State University, Stavropolskaya St. 149, 350040 Krasnodar, Russia

## Abstract

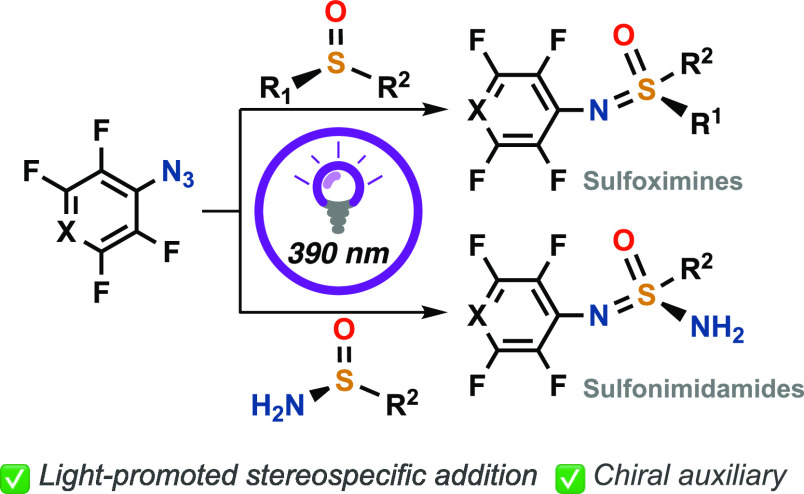

Sulfonimidamides (SIAs) and sulfoximines (SOIs) have attracted attention due to their potential in agriculture and in medicinal chemistry as bioisosteres of biologically active compounds, and new synthetic methods are needed to access and explore these compounds. Herein, we present a light-promoted generation of perfluorinated aromatic nitrenes, from perfluorinated azides, that subsequently are allowed to react with sulfinamides and sulfoxides, generating achiral and chiral SIAs and SOIs. One of the enantiopure SIAs was evaluated as a novel chiral auxiliary in Grignard additions to the imines yielding the product in up to 96:4 diastereomeric ratio.

## Introduction

**Figure 1:**
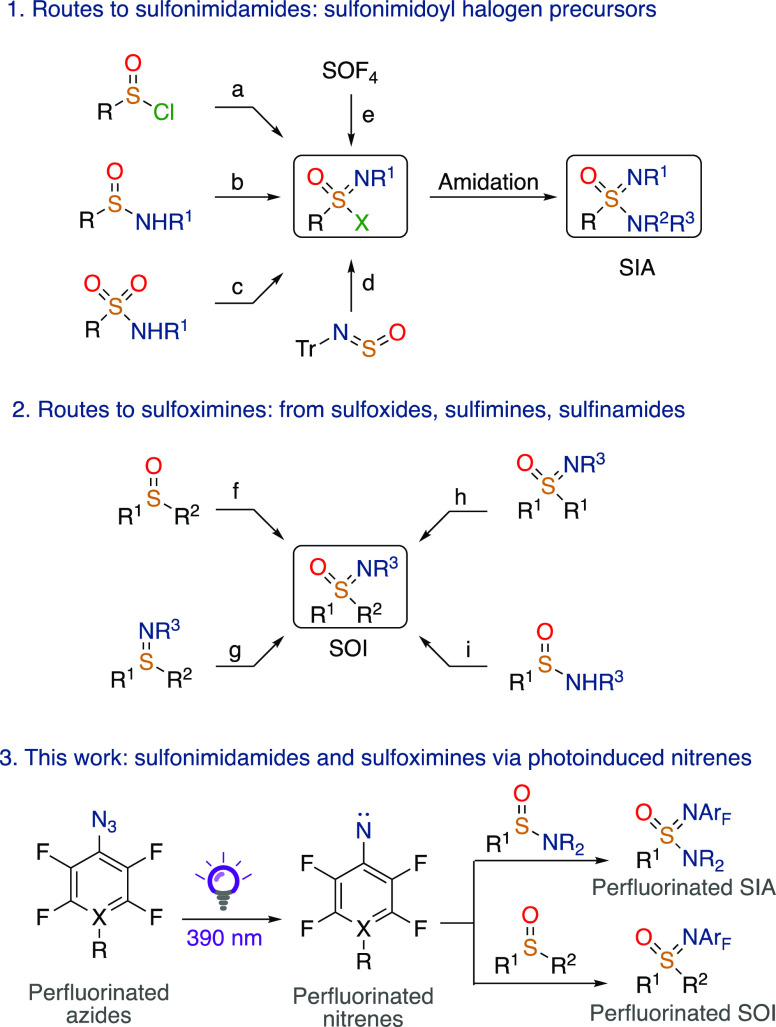
(1) Routes to SIAs: (a) oxidative imidation, (b) oxidative chlorination, (c) deoxychlorination, (d) Grignard addition and chlorination, (e) sulfur–fluorine exchange via sulfinimidoyl fluoride; (2) routes to SOIs: (f) imidation, (g) oxidation, (h) desymmetrization of SOIs, and (i) *S*-alkylation; and (3) this work: SIAs and SOIs via photogenerated nitrenes.

During the last decades, the utility of sulfonimidamides (SIAs)^1−5^ and sulfoximines (SOIs)^6−12^ has been demonstrated in synthesis, agrochemical applications, and as bioisosteres in medicinal chemistry due to their notable properties, such as basicity, nucleophilicity, and solubility in polar solvents. The classical synthetic routes^13^ to access SIAs usually rely on the formation of sulfonimidoyl chloride as a precursor, followed by an amidation reaction (Figure 1).

Sulfonimidoyl chloride can be generated in several different ways, such as oxidative imidation (Figure 1a),^14^ oxidative chlorination (Figure 1b),^15^ deoxychlorination^16^ (Figure 1c), and via Grignard addition to a sulfinylamine, followed by chlorination (Figure 1d).^17^ Similarly, sulfur–fluorine exchange reactions (Figure 1e) with sulfonimidoyl fluoride as the key intermediate have been used to yield SIAs.^18,19^ Other approaches to form SIAs involve copper-catalyzed transamidation of sulfinamides (SAs) or copper-catalyzed oxidation of methyl SOIs.^20,21^ Furthermore, several metal-free approaches using N–H transfer to SAs have been disclosed.^22^

One of the most convenient ways to synthesize chiral SOIs^23^ involves the formation of a sulfur–nitrogen bond between chiral SOs and nitrenes, either using metal-catalyzed procedures (Fe, Rh, and Ag)^24−28^ or hypervalent iodine or bromine reagents (Figure 1f).^29−32^ Other approaches involve stereospecific oxidation of enantioenriched sulfinimines (Figure 1g), desymmetrization of homochiral SOIs^33,34^ (Figure 1h), and stereospecific S—alkylation of chiral SOIs (Figure 1i).^35^

The introduction of fluoro-substituents into drug-like molecules and agrochemicals can tremendously affect their properties by, for example, decreasing their basicity and improving their bioavailability,^36−38^ and in this context, several different methods to synthesize fluorinated SOIs were developed.^39−46^ In addition, SOIs containing N–C_aryl–F_ bonds can be accessed either via copper-catalyzed direct sulfoximination or via S_N_Ar.^47,48^ An alternative approach is to incorporate aromatic fluorinated moieties via perfluorinated aromatic azides (PFAAs). Phenyl azides can generate, either via photo- or thermolysis, highly reactive nitrenes that rapidly rearrange via ring-expansion to form ketenimines. These ketenimines will ultimately lead to polymeric tar unless intercepted with a good nucleophile.^49^ On the contrary, PFAAs are regarded as superior phenylnitrene precursors, enabling higher yields of the C–H and N–H insertion products.^50,51^ The improved selectivity is attributed to the “*ortho*-difluoro effect” where fluorine atoms in the ortho-position to the azide effectively retard the ring-expansion pathway and instead promote a long-lived singlet nitrene that is responsible for the productive bimolecular reaction.^52^ The reaction between dimethyl sulfoxide (DMSO) and perfluorinated phenylnitrene, generated via the thermolysis of 4-azido-2,3,5,6-tetrafluoropyridine, was first observed by Banks and Sparkes,^50^ but no attempts to expand the nitrene-promoted coupling between PFAAs and SOs or related derivatives were undertaken. In this work, we investigated a light-promoted approach to *ortho*-fluoro nitrenes from PFAAs, leading to the stereospecific addition to SAs and SOs. In addition, one of the chiral SIAs was evaluated as a chiral auxiliary in the stereoselective addition of Grignard reagents to SIA-derived imines, yielding the addition products in high stereoselectivity (up to 96:4).

## Results and Discussion

Upon the irradiation of PFAA (**1a**) in DMSO with a 390 nm light-emitting diode (LED) light, we noticed the formation of an SOI adduct between the in situ generated perfluoroaryl nitrene and DMSO. In our group, we have previously developed procedures for the catalytic formation of sulfinimines from chiral SAs and aldehydes,^53,54^ and therefore, we became interested in investigating the reactivity between perfluoroaryl nitrenes and optically active SAs or SO. Our initial screening started with **1a** and (*S*)-*tert*-butylsulfinamide in different solvents and with an irradiation of 390 nm light for 1.5 h at room temperature. In most of the solvents (Table 1, entries 1–9), SIA (*S*)-**1** is formed together with varying amounts of the perfluorinated aniline.

**Table 1:**
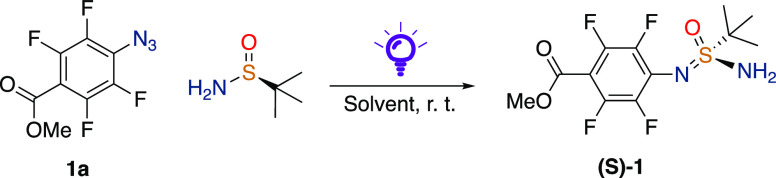
Optimization of Reaction Conditions for the Synthesis of (*S*)-**1**^a^

entry	solvent	yield (*S*)-**1** (%)^b^	aniline (%)^b^
1	THF	23	77
2	EtOH	21	59
3	toluene	52	21
4	acetone	51	9
5	CH_2_Cl_2_	47	4
6	CHCl_3_	57	6
7	MeCN	47	6
8	EtOAc	65	5
9	PhCF_3_	66	4
10	DMF	–b	–
11	H_2_O	0^b^	1

a Reaction conditions: azide (0.075 mmol, 0.05 M), (*S*)-*tert*-butylsulfinamide (0.15 mmol, 1.5 equiv), degassed solvent (1.5 mL), and 390 nm Kessil LED light, 1.5 h.

b Determined by ^1^H NMR with an internal standard.

In tetrahydrofuran (THF) and ethanol, perfluorinated aniline was the major product (Table 1, entries 1–2), while reactions in toluene, acetone, dichloromethane, chloroform, and acetonitrile led to increased yields of (S)-**1** and with less formation of the aniline derivative (Table 1, entries 3–7). The highest yields, together with the lowest formation of side products, were obtained in ethyl acetate and α,α,α-trifluorotoluene (PhCF_3_) (Table 1, entries 8 and 9), while DMF gave a complex mixture of fluorinated products and the reaction in water led to the formation of the perfluorinated azo-compound mainly (Table 1, entries 10–11). The reaction also proceeded using blue light (440 nm), but the reaction times increased significantly (about 10 times).

Next, we explored the substrate scope of the photopromoted coupling between enantiopure *tert*-butylsulfinamides and different PFAAs using PhCF_3_ as the solvent (Table 2). Methyl 4-azidotetrafluorobenzoate reacted with both (*S*)- and (*R*)-*tert*-butylsulfinamides to form SIAs (*S*)-**1** and (*R*)-**1** in good yields (66 and 65%, respectively) and without the loss of enantiopurity, as determined by chiral high-performance liquid chromatography (HPLC). The cyano-substituted PFAA derivative showed increased reactivity than the ester-containing substrate and yielded product (*S*)-**2** in 62% yield upon irradiation at 390 nm for merely 2 h in the presence of (*S*)-*tert*-butylsulfinamide. The pyridine-based PFAA gave similar yields toward the formation of products (*S*)-**3** and (*R*)-**3** (64 and 65%, respectively) but required a considerably longer irradiation time (16 h). Next, the reaction was extended to other PFAA derivatives, such as pentafluoroazidobenzene and 4-azido-tetrafluorobenzoic acid. However, this afforded lower yields of the target products (*S*)-**4** and (*S*)-**5** (32 and 34%, respectively) compared to the other derivatives (**1**–**3**). This highlights the importance of the substituent in para-position in influencing the reactivity of the photogenerated nitrene.

**Table 2:**
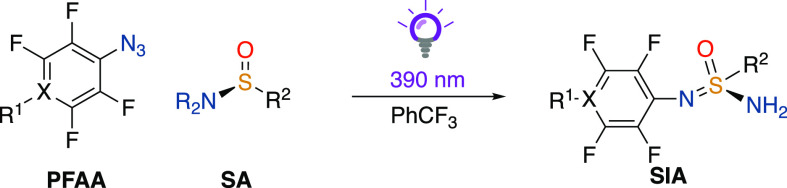

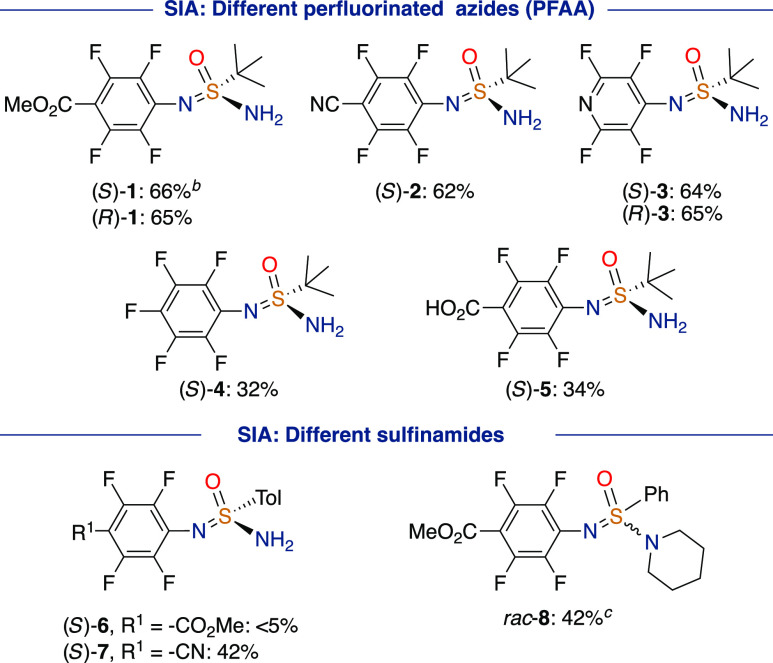
Synthesis of SIAs from PFAA and SAs^a^

a Reaction conditions: PFAA (0.3–0.9 mmol, 0.05 M), SA (0.45–1.35 mmol, 1.5 equiv), degassed PhCF_3_, 390 nm Kessil LED light, 2–16 h, r.t.

b Average yield of two syntheses.

c From the racemic starting material.

The photopromoted reaction of PFAAs with *p*-toluenesulfinamide was less satisfying, and (*S*)-**6** was only obtained in trace amounts together with other side products. Better results were obtained for the more reactive cyano-substituted PFAA yielding the product (*S*)-**7** in 42% yield. The poorer reactivity was ascribed to the scarce solubility of *p*-tolylsulfinamide compared to that of *tert*-butylsulfinamide. A secondary SA, racemic 1-(phenylsulfinyl)piperidine, was made to react with methyl 4-azidotetrafluorobenzoate to yield the target product *rac*-**8** in 42% yield.

In addition to the synthesis of perfluorinated SIAs, the generality of the nitrene addition was expanded through reactions with SOs to yield perfluorinated SOIs (Table 3).

**Table 3:**
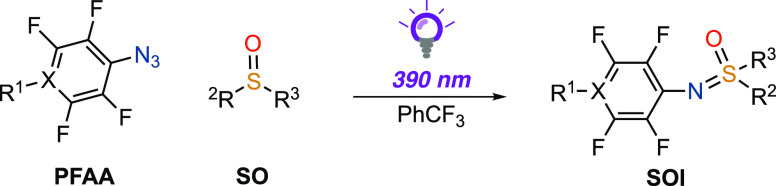

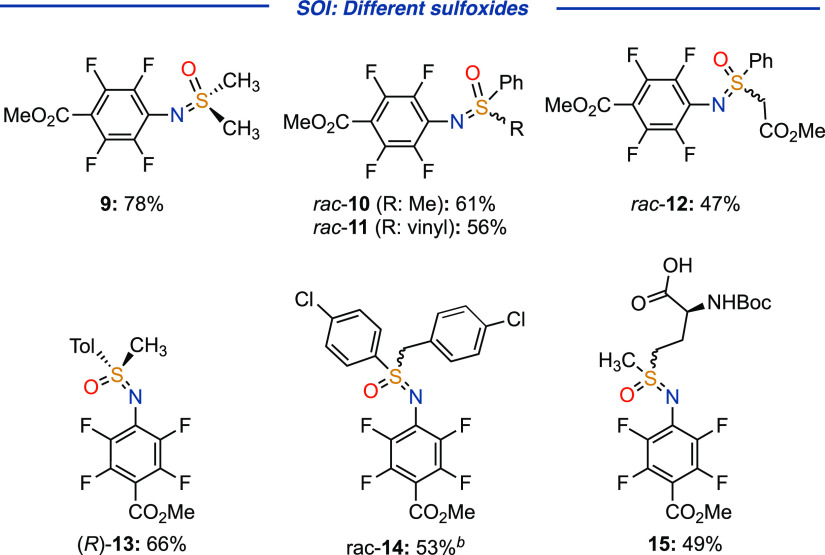
Synthesis of SOIs from PFAA and SOs^a^

a Reaction conditions: PFAA (0.3 mmol, 0.05 M), SO (0.45 mmol, 1.5 equiv), degassed PhCF_3_, 390 nm Kessil LED light, 1–4 h, r.t.

b EtOAc as the solvent.

The photopromoted PFAA-nitrenes readily reacted with SOs to form SOIs and did not react further under prolonged light irradiation. This differs from the reactivity in the work by Bolm and coworkers where they observed the light-promoted formation of nitrenes from SOIs.^55^ For example, DMSO reacted with the photogenerated nitrene to yield SOI **9** in high yield (78%) after only 2 h. A fast reaction was also observed for racemic methyl phenyl SO, yielding product **10** in good yield (61%). Racemic phenylvinyl SO led to the formation of product **11** (56%) without affecting the double bond. The lower yield was accompanied by an increased formation of the corresponding aniline derivative (methyl 4-amino-2,3,5,6-tetrafluorobenzoate), which was also observed in the reaction with racemic methyl 2-phenylsulfinylacetate, affording *rac*-**12** in 47% yield. An enantiomerically pure SO was also converted to (*R*)-**13** in a stereospecific addition of the PFAA-nitrene in 66% yield. Furthermore, the reaction was feasible with the racemic SO derived from the pesticide chlorbensid, but due to poor solubility in PhCF_3_, ethyl acetate was used as the solvent, yielding product *rac*-**14** in moderate yield (53%) after 2 h. Finally, the reaction was tested with methionine SO, derived from the oxidized form of the amino acid l-methionine, which is associated with aging when present in increased levels in tissues.^56,57^ The Boc-protected SO yielded the target product **15** after merely 1 h and was obtained in 49% yield again with an increased formation of the aniline derivative as the side product.

The use of enantiopure *tert*-butylsulfinamide is an established strategy to access valuable chiral amines. In the standard approach, the chiral auxiliary group is introduced via condensation with aldehydes or ketones, followed by stereoselective nucleophilic addition and chiral auxiliary removal to yield the enantioenriched amine.^58,59^ We hypothesized that the free NH_2_ group in enantiomerically pure SIAs could act as a chiral auxiliary via the reaction with carbonyl compounds to yield imines, which could subsequently be used in stereoselective addition reactions. Previously, SIAs were used in asymmetric reactions as ligands,^60,61^ organocatalysts,^62^ or nitrene-transfer agents,^63−71^ but there are no reports of SIAs as chiral auxiliaries.

**Scheme 1:**
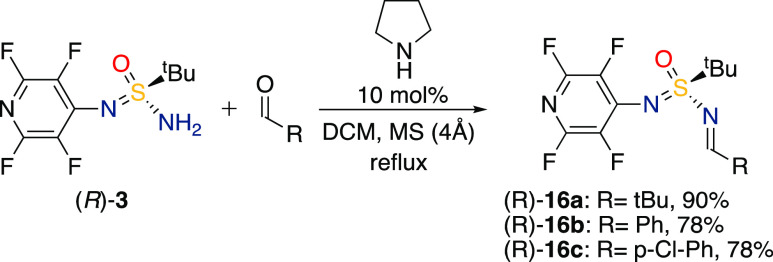
Synthesis of Imines from SIA and Aldehydes Reaction conditions: (*R*)-**3** (0.8 mmol, 0.1 M), aldehyde (2 equiv), pyrrolidine (0.1 equiv), CH_2_Cl_2_ (8 mL, dry), molecular sieves (4 Å), reflux, N_2_ atmosphere.

Indeed, the chiral pyridine-based (*R*)-**3** formed stable imines from pivaldehyde and aromatic benzaldehydes using reaction conditions reported by Cid and coworkers.^72^ The reactions proceeded to completion after 20–44 h at reflux in CH_2_Cl_2_, yielding the target imines in high to excellent yields (78–90%, Scheme 1).

Unfortunately, enolizable aldehydes, such as butyraldehyde, led to a complex reaction mixture with side products. The obtained imine derivatives **16a–c** were used to investigate the ability of SIAs to function as chiral auxiliaries in stereoselective carbonyl addition reactions with Grignard reagents.

**Scheme 2:**
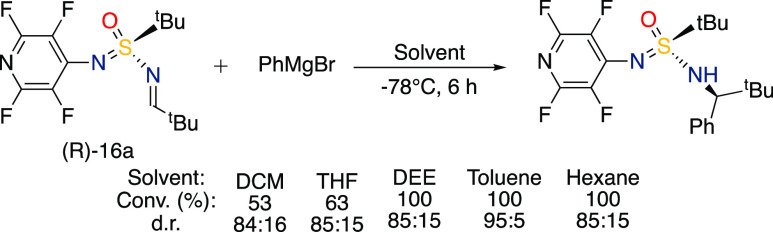
Solvent Screening for Grignard Addition to SIA Imines Reaction conditions: phenylmagnesium bromide (0.14 mmol, 2.5 equiv), imine (0.057 mmol, 1 equiv), solvent (0.5 mL, dry), N_2_ atmosphere, −78 °C.

Initially, the addition of phenylmagnesium bromide to imine (*R*)-**16a** was investigated in several different solvents (Scheme 2). After 6 h of the reaction at −78 °C, the results revealed that both CH_2_Cl_2_ and THF failed to give full conversion, while toluene provided full conversion and high stereoselectivity according to ^1^H NMR. Conducting the reaction in diethylether and hexane also gave full conversion of the starting material but with slightly lower diastereoselectivity.

With the optimal reaction conditions in hand, we investigated the scope of the Grignard addition to imines derived from SIA (*R*)-**3**. The imines were made to react with Grignard reagents at −78 °C for 6 h, and the reaction mixtures were allowed to reach room temperature overnight. The reaction was quenched and extracted, and the product yield was determined using ^1^H NMR with *tert*-butylmethyl ether as the internal standard.

Addition of aromatic Grignard regents (Table 4, entries 1–3) to the imine derived from pivaldehyde gave the addition product in high yields (86–98%) and with high diastereomeric ratios (up to 96:4) which are comparable to Grignard additions to *tert*-butyl sulfinyl imines.^73^ Methyl magnesium bromide yielded the product (86%) but with much lower selectivity compared to *tert*-butyl sulfinimines,^73^ while aliphatic isopropylmagnesium chloride gave only small amounts of the addition product together with the reduced product derived from a hydride transfer (Table 4, entries 4–5). The addition of aromatic Grignard reagents to the SIA imine derived from aromatic benzaldehyde provided products in high yields and diastereomeric ratios (Table 4, entries 6–10) that are comparable with the selectivities obtained with the *tert*-butyl sulfinyl imines.^74,75^ Methyl magnesium bromide gave low selectivity in toluene, while an improved selectivity (dr 84:16) was observed in CH_2_Cl_2_ (Table 4, entry 8).

**Table 4:**
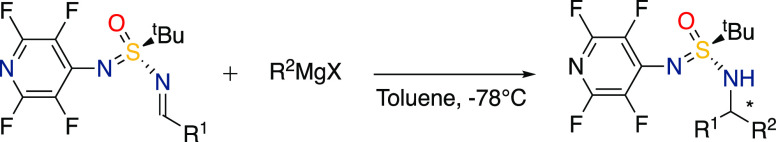
Scope of the Addition of Grignard Reagents to Imines Derived from SIAs^a^

entry	R_1_	R_2_	X	yield^b^	dr^c^
1	^*t*^Bu	Ph	Br	86	95:5
2	^*t*^Bu	3-methoxy-C_6_H_4_	Br	90	96:4
3	^*t*^Bu	4-chloro-C_6_H_4_	Br	98	93:7
4	^*t*^Bu	Me	Br	86	67:33
5	^*t*^Bu	^*i*^Pr	Cl		
6	Ph	3-methoxy-C_6_H_4_	Br	85	92:8
7	Ph	4-chloro-C_6_H_4_	Br	85	92:8
8	Ph	Me	Br	80	84:16^d^
9	4-chloro-C_6_H_4_	Ph	Br	86	84:16
10	4-chloro-C_6_H_4_	3-methoxy-C_6_H_4_	Br	90	94:6

a Reaction conditions: imine (0.05 mmol, 1 equiv), Grignard reagent (2.5 equiv), toluene (0.5 mL), −78 to r.t.

b The yield was determined by ^1^H NMR spectroscopy using *tert*-butyl methyl ether as the internal standard.

c Determined by ^1^H NMR spectroscopy or chiral HPLC.

d Reaction performed in CH_2_Cl_2_.

**Scheme 3:**
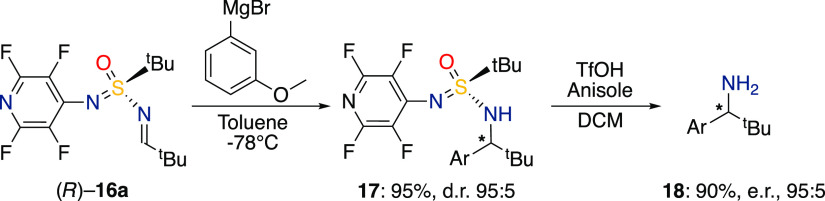
Larger Scale Synthesis and Removal of Chiral Auxiliary Reaction conditions: 3-methoxyphenylmagnesium bromide (0.75 mmol, 2.5 equiv), imine (0.3 mmol, 1.0 equiv), toluene (2.5 mL, dry), N_2_-atmosphere −78 °C to r.t. Compound **17** (0.09 mmol, 1 equiv), anisole (20 equiv), TfOH (9 equiv), CH_2_Cl_2_, 0 °C to r.t.

Finally, we performed the synthesis between imine **16a** and 3-methoxyphenylmagnesium bromide in a 0.3 mmol scale which gave product **17** in 95% yield and 95:5 diastereomeric ratio (Scheme 3).

The classical approach used to cleave the SA auxiliary involves acidic condition in protic solvents, typically HCl or trifluoroacetic acid in methanol.^76,77^ Unfortunately, those conditions did not work and a complex reaction mixture was obtained. Finally, the SIA chiral auxiliary was removed by treatment with triflic acid and anisole in CH_2_Cl_2_^78^ and the amine was obtained in 90% yield and 95:5 dr (Scheme 3).

## Conclusions

We have developed a photopromoted reaction between perfluorinated aromatic azides and SAs or SOs to obtain SIAs and SOIs, respectively. The fluoro substituents on the aromatic ring of the azides were critical for accessing synthetically useful nitrenes. The reaction proceeded via in situ generated perfluorinated nitrenes and stereospecific addition, enabling the formation of optically pure compounds. One of the chiral SIAs, derived from the perfluorinated pyridine azide, was condensed with aliphatic and aromatic aldehydes to yield enantiopure imine-derivatives in good to excellent yields. The use of the synthesized SIA was evaluated as a potential chiral auxiliary for the addition of Grignard reagents to the chiral SIA-derived imines at −78 °C in toluene. The investigation demonstrated that Grignard reagents were successfully added to the imines in high to excellent yields (up to 98%) and good to excellent diastereoselectivity (up to 96:4 dr). The use of SIA as a chiral auxiliary is to the best of our knowledge unprecedented, and we believe that these new types of SIAs find applications as novel scaffolds in asymmetric synthesis.

## Experimental Section

All reagents were obtained from commercial sources and used without further purification. The perfluorinated aromatic azides were synthesized according to the literature.^79^ All solvents were purified and dried according to standard methods prior to use, unless stated otherwise. Degassed solvents were obtained by bubbling the solvent with inert gas through a needle. Anhydrous dichloromethane was obtained by distillation over calcium hydride, and anhydrous diethyl ether, THF, and toluene were obtained from a Glass Contour solvent dispensing system. Heating of reaction mixtures was performed in oil baths, and experiments at lower temperatures (−78 °C) were carried out with dry ice/acetone baths. Thin-layer chromatography (TLC) was performed using 60 mesh silica gel plates visualized with short-wavelength UV light (254 nm). Silica gel 60 (200–300 mesh) was used for column chromatography. HPLC analyses were conducted using a UV detector (Shimadzu SPD-20A) and a chiral column (Kromasil 5-CelluCoat RP, 0.46 × 25 cm) using a flow of 1.0 mL/min of the eluent system hexane/*iso*-propanol. A Bruker Ascend 400 spectrometer (400 MHz) or Bruker Avance DMX 500 (500 MHz) spectrometer was used for the recording of ^1^H NMR spectra, ^13^C{1H} NMR spectra, and ^19^F NMR spectra. Proton chemical shifts are reported as δ values (ppm) relative to tetramethylsilane with residual undeuterated CHCl_3_ (δ 7.26), DMSO-*d*_6_ (δ 2.50), and methanol-*d*_4_ (δ 3.31) as internal standards. ^13^C{1H} chemical shifts are reported as δ values (ppm) relative to tetramethylsilane with CDCl_3_ (δ 77.16 ppm), DMSO-*d*_6_ (δ 39.52 ppm), or methanol-*d*_4_ (δ 49.0 ppm) as internal standards. Data for ^1^H NMR are reported as follows: chemical shift (δ, ppm) and multiplicity (s = singlet, d = doublet, t = triplet, q = quartet, m = multiplet or unresolved, br = broad singlet, and *J* = coupling constants in Hz, integration). High-resolution mass spectrometry (HRMS) measurements were performed on methanolic solutions of the compounds using a Bruker maXis impact II micrOTOF spectrometer [direct injection and electrospray ionization (ESI)]. The light-promoted reactions were run using a 390 nm light source (40 W, Kessil PR160, set to maximum intensity) at a distance of 3.0 cm from the reaction vessel. Experimental details, such as spectroscopic characterizations (^1^H, ^13^C{1H}, and ^19^F NMR), HPLC chromatograms, and HRMS, are given in the Supporting Information.

### General Procedure A for the Synthesis of SIAs

To an 8 mL vial equipped with a magnetic stir bar, the perfluorinated aromatic azido (PFAA) compound (1 equiv, 0.3 mmol, 0.05 M), SA (1.5 equiv, 0.45 mmol), and degassed α,α,α-trifluorotoluene (PhCF_3_) (6 mL) were added. At this point, the vial was evacuated and back filled with N_2_, and the vial was capped with a rubber septum. The reaction mixture was irradiated at 390 nm (40 W, Kessil PR160, set to maximum intensity, 3.0 cm from the reaction vessel) while stirring. After the completion of the reaction, the crude obtained upon solvent removal under reduced pressure was purified by flash column chromatography using either petroleum ether and ethyl acetate (PE/EtOAc) or petroleum ether, dichloromethane, and ethyl acetate (PE/DCM/EtOAc) as the eluent system to afford the pure product. All compounds were characterized via HRMS and ^1^H NMR, ^13^C{1H} NMR, and ^19^F NMR spectroscopies.

#### Methyl (*S*)-4-((Amino(*tert*-butyl)(oxo)-λ^6^-sulfaneylidene)amino)-2,3,5,6-tetrafluorobenzoate (*S*)-**1**

The compound was obtained according to general procedure A using azide **1a** (75 mg, 0.3 mmol, 1 equiv) and (*S*)-*tert*-butylsulfinamide (64 mg, 0.5 mmol, 1.7 equiv). The reaction was completed after 6 h of the reaction. The pure product was obtained after flash column chromatography (eluent: PE/DCM/EtOAc, 6:1:1 → 3:1:1) (rf: 0.25, eluent: 4:1:1) as a pale-yellow precipitate (72 mg, 70%). HPLC (Kromasil 5-CelluCoat RP, 0.46 cm × 25 cm, *n*-hexane/isopropanol = 90/10, flow rate = 1.0 mL/min, λ = 220 nm) *t*_R_ = 22.8 min (major), 41.4 min (minor). mp: 154–155 °C. ^1^H NMR (CDCl_3_, 400 MHz): δ 4.31 (br, 2H, NH_2_), 3.94 (s, 3H, OCH_3_), and 1.60 (s, 9H, *t*-Bu); ^13^C{^1^H} NMR (CDCl_3_, 100 MHz): δ 161.1, 146.0 (dm, *J* = 256 Hz), 142.9 (dm, *J* = 243 Hz), 127.4, 105.1, 62.1, 53.0, and 24.3; ^19^F NMR (CDCl_3_, 376 MHz): δ −141.1 (m, 2F) and −149.2 (m, 2F). HRMS (ESI-TOF) *m*/*z*: [M + Na]^+^ calcd for C_12_H_14_F_4_N_2_O_3_SNa, 365.0554; found, 365.0554. [α]_D_^20^ + 32 (*c* 0.5, CHCl_3_).

#### Methyl (*R*)-4-((Amino(*tert*-butyl)(oxo)-λ^6^-sulfaneylidene)amino)-2,3,5,6-tetrafluorobenzoate (*R*)-**1**

The compound was obtained according to general procedure A using azide **1a** (77 mg, 0.3 mmol, 1 equiv) and (*R*)-*tert*-butylsulfinamide (55 mg, 0.5 mmol, 1.4 equiv). The reaction was completed after 6 h of the reaction. The pure product was obtained after flash column chromatography (eluent: PE/DCM/EtOAc, 6:1:1 → 3:1:1) (rf: 0.25, eluent: 4:1:1) as a pale-yellow precipitate (68 mg, 65%). HPLC (Kromasil 5-CelluCoat RP, 0.46 cm × 25 cm, *n*-hexane/isopropanol = 90/10, flow rate = 1.0 mL/min, λ = 220 nm) *t*_R_ = 23.4 min (minor), 41.7 min (major). mp: 150–153 °C. ^1^H NMR (CDCl_3_, 400 MHz): δ 4.49 (br, 2H, NH_2_), 3.93 (s, 3H, OCH_3_), and 1.58 (s, 9H, *t*-Bu); ^13^C{^1^H} NMR (CDCl_3_, 100 MHz): δ 161.1, 145.9 (dm, *J* = 256 Hz), 142.9 (dm, *J* = 244 Hz), 127.6, 104.9, 62.1, 53.0, and 24.3; ^19^F NMR (CDCl_3_, 376 MHz): δ −141.3 (m, 2F), −149.2 (m, 2F). HRMS (ESI-TOF) *m*/*z*: [M + Na]^+^ calcd for C_12_H_14_F_4_N_2_O_3_SNa, 365.0554; found, 365.0555. [α]D20 – 32 (*c* 0.5, CHCl_3_).

#### (*S*)-*N*′-(4-Cyano-2,3,5,6-tetrafluorophenyl)-2-methylpropane-2-sulfonimidamide (*S*)-**2**

The compound was obtained according to general procedure A using azide **1b** (68 mg, 0.3 mmol, 1 equiv) and (*S*)-*tert*-butylsulfinamide (54 mg, 0.5 mmol, 1.5 equiv). The reaction was completed after 2 h of the reaction. The pure product was obtained after flash column chromatography (eluent: PE/DCM/EtOAc, 10:1:1 → 6:1:1) (rf: 0.15, eluent: 6:1:1) as an off-white precipitate (60 mg, 62%). mp: 126–127 °C. ^1^H NMR (CDCl_3_, 400 MHz): δ 4.46 (br, 2H, NH_2_), and 1.59 (s, 9H, *t*-Bu); ^13^C{^1^H} NMR (CDCl_3_, 125 MHz): δ 147.8 (dm, *J* = 259 Hz), 142.5 (dm, *J* = 246 Hz), 130.9, 108.6, 85.95, 62.6, and 24.2; ^19^F NMR (CDCl_3_, 376 MHz): δ −135.4 (m, 2F), −146.9 (m, 2F). HRMS (ESI-TOF) *m*/*z*: [M + Na]^+^ calcd for C_11_H_11_F_4_N_3_OSNa, 332.0452; found, 332.0451. [α]D27 + 68 (*c* 0.4, CHCl_3_).

#### (*S*)-2-Methyl-*N*′-(perfluoropyridin-4-yl)propane-2-sulfonimidamide (*S*)-**3**

The compound was obtained according to general procedure A using azide **1c** (62 mg, 0.3 mmol, 1 equiv) and (*S*)-*tert*-butylsulfinamide (56 mg, 0.5 mmol, 1.5 equiv). The reaction was completed after 16 h of the reaction. The pure product was obtained after flash column chromatography (eluent: PE/DCM/EtOAc, 8:1:1 → 4:1:1) as an off-white precipitate (58 mg, 64%). mp: 123–125 °C. ^1^H NMR (CDCl_3_, 400 MHz): δ 4.44 (br, 2H, NH_2_) and 1.60 (s, 9H, *t*-Bu); ^13^C{^1^H} NMR (CDCl_3_, 125 MHz): δ 144.1 (dm, *J* = 241 Hz), 137.6 (dm, *J* = 253 Hz), 136.1, 62.4, and 24.0; ^19^F NMR (CDCl_3_, 376 MHz): δ −93.2 (m, 2F) and −151.5 (m, 2F). HRMS (ESI-TOF) *m*/*z*: calcd for C_9_H_11_F_4_N_3_OS [M + Na]^+^, 308.0451; found, 308.0449. [α]_D_^30^ + 280 (*c* 0.3, acetonitrile).

#### (*R*)-2-Methyl-*N*′-(perfluoropyridin-4-yl)propane-2-sulfonimidamide (*R*)-**3**

The compound was obtained according to general procedure A using azide **1c** (58 mg, 0.3 mmol, 1 equiv) and (*R*)-*tert*-butylsulfinamide (51 mg, 0.5 mmol, 1.5 equiv). The reaction was completed after 16 h of the reaction. The pure product was obtained after flash column chromatography (eluent: PE/DCM/EtOAc, 6:1:1 → 3:1:1) (rf: 0.3, eluent: 4:1:1) as an off-white precipitate (56 mg, 65%). mp: 122–123 °C. ^1^H NMR (CDCl_3_, 400 MHz): δ 4.37 (br, 2H, NH_2_) and 1.60 (s, 9H, *t*-Bu); ^13^C{^1^H} NMR (CDCl_3_, 100 MHz): δ 144.2 (dm, *J* = 241 Hz), 137.5 (dm, *J* = 251 Hz), 62.6 and 24.2; ^19^F NMR (CDCl_3_, 376 MHz): δ −93.1 (m, 2F) and −151.5 (m, 2F). HRMS (ESI-TOF) *m*/*z*: [M + Na]^+^ calcd for C_9_H_11_F_4_N_3_OSNa, 308.0451; found, 308.0451. [α]_D_^30^ – 290 (*c* 0.4, acetonitrile).

#### (*S*)-2-Methyl-*N*′-(perfluorophenyl)propane-2-sulfonimidamide (*S*)-**4**

The compound was obtained according to general procedure A using azide **1d** (65 mg, 0.3 mmol, 1 equiv) and (*S*)-*tert*-butylsulfinamide (54 mg, 0.5 mmol, 1.5 equiv). The reaction was completed after 19 h of the reaction. The pure product was obtained after flash column chromatography (eluent: PE/DCM/EtOAc, 10:1:1 → 6:1:1) (rf: 0.28, eluent: 6:1:1) as an off-white precipitate (30 mg, 32%). mp: 101–103 °C. ^1^H NMR (CDCl_3_, 400 MHz): δ 4.27 (br, 2H, NH_2_) and 1.59 (s, 9H, *t*-Bu); ^13^C{^1^H} NMR (CDCl_3_, 125 MHz): δ 143.4 (dm, *J* = 243 Hz), 138.0 (dm, *J* = 257 Hz), 137.8 (dm, *J* = 246 Hz), 118.6, 61.5, and 24.2; ^19^F NMR (CDCl_3_, 376 MHz): δ −150.1 (m, 2F), −164.2 (m, 1F), and −164.5 (m, 2F). HRMS (ESI-TOF) *m*/*z*: [M + Na]^+^ calcd for C_10_H_11_F_5_N_2_OSNa, 325.0405; found, 325.0404. [α]_D_^27^ + 28 (*c* 0.3, CHCl_3_).

#### (*S*)-4-((Amino(*tert*-butyl)(oxo)-λ^6^-sulfaneylidene)amino)-2,3,5,6-tetrafluorobenzoic Acid (*S*)-**5**

The compound was obtained according to general procedure A using azide **1e** (68 mg, 0.3 mmol, 1 equiv) and (*S*)-*tert*-butylsulfinamide (57 mg, 0.5 mmol, 1.5 equiv). The reaction was completed after 10 h of the reaction. The pure product was obtained after flash column chromatography (eluent: 5% MeOH in CH_2_Cl_2_ + 0.5% formic acid) (rf: 0.19, eluent: 5% MeOH in CH_2_Cl_2_ + 0.5% formic acid) as a white precipitate (31 mg, 34%). mp: 74 °C. ^1^H NMR (DMSO-*d*_6_, 400 MHz): δ 6.88 (br, 2H, NH_2_) and 1.43 (s, 9H, *t*-Bu); ^13^C{^1^H} NMR (DMSO-*d*_6_, 125 MHz): δ 161.0, 144.9 (dm, *J* = 249 Hz), 141.6 (dm, *J* = 243 Hz), 129.1, 103.8, 60.6, and 23.8; ^19^F NMR (DMSO-*d*_6_, 376 MHz): δ −143.5 (m, 2F) and −148.8 (m, 2F). HRMS (ESI-TOF) *m*/*z*: [M + Na]^+^ calcd for C_11_H_12_F_4_N_2_O_3_SNa, 351.0397; found, 351.0399. [α]_D_^31^ – 4 (*c* 0.4, methanol).

#### (*S*)-*N*′-(4-Cyano-2,3,5,6-tetrafluorophenyl)-4-methylbenzenesulfonimidamide (S)-**7**

The compound was obtained according to general procedure A using azide **1b** (62 mg, 0.3 mmol, 1 equiv) and (*S*)-*p*-toluenesulfinamide (55 mg, 0.4 mmol, 1.3 equiv). The reaction was completed after 5 h of the reaction. The pure product was obtained after flash column chromatography (eluent: PE/DCM/EtOAc, 8:1:1 → 6:1:1) (rf: 0.12, eluent: 6:1:1) as an off-white precipitate (41 mg, 42%). mp: 175–177 °C. ^1^H NMR (DMSO-*d*_6_, 500 MHz): δ 7.85 (d, *J* = 8.3 Hz, 1H), 7.78 (br, 2H, NH_2_), 7.43 (d, *J* = 8.1 Hz, 1H), and 2.39 (s, 3H, CH_3_); ^13^C{^1^H} NMR (DMSO-*d*_6_, 125 MHz): δ 147.3 (dm *J* = 258 Hz), 143.0, 141.0 (dm *J* = 244 Hz), 140.4, 132.0, 129.6, 126.3, 109.0, 83.2, and 21.0; ^19^F NMR (DMSO-*d*_6_, 376 MHz): δ −134.9 (m, 2F) and −146.4 (m, 2F). HRMS (ESI-TOF) *m*/*z*: [M + Na]^+^ calcd for C_14_H_9_F_4_N_3_OSNa, 366.0295; found, 366.0296. [α]_D_^31^ – 49 (*c* 0.3, acetonitrile).

#### Methyl 2,3,5,6-Tetrafluoro-4-((oxo(phenyl)(piperidin-1-yl)-λ^6^-sulfaneylidene)amino)-benzoate *rac*-**8**

The compound was obtained according to general procedure A using azide **1a** (75 mg, 0.3 mmol, 1 equiv) and 1-(phenylsulfinyl)piperidine (94 mg, 0.45 mmol, 1.5 equiv). The reaction was completed after 6 h of the reaction. The pure product was obtained after flash column chromatography (eluent: PE/DCM/EtOAc, 20:1:1 → 10:1:1) as a white precipitate (51 mg, 42%). mp: 85–86 °C. ^1^H NMR (CDCl_3_, 400 MHz): δ 7.94 (m, 2H), 7.62 (m, 1H), 7.56 (m, 2H), 3.93 (s, 3H), 3.05 (m, 4H), 1.54 (m, 4H), and 1.40 (m, 2H); ^13^C{^1^H} NMR (CDCl_3_, 125 MHz): δ 161.1, 145.9 (dm, *J* = 255 Hz), 142.5 (dm, *J* = 245 Hz), 136.3, 133.1, 129.3, 128.1, 127.4, 104.5, 52.9, 47.6, 25.5, and 23.6; ^19^F NMR (CDCl_3_, 376 MHz): δ −141.2 (m, 2F) and −147.8 (m, 2F). HRMS (ESI-TOF) *m*/*z*: [M + H]^+^ calcd for C_19_H_19_F_4_N_2_O_3_S, 431,1047; found, 431.1049.

### General Procedure B for the Synthesis of SOIs

To an 8 mL vial equipped with a magnetic stir bar, PFAA compound (1 equiv, 0.3 mmol, 0.05 M), SO (1.5 equiv, 0.45 mmol), and degassed α,α,α-trifluorotoluene (PhCF_3_) (6 mL) were added. At this point, the vial was evacuated and back filled with N_2_, and the vial was capped with a rubber septum. The reaction mixture was irradiated at 390 nm (40 W, Kessil PR160, set to maximum intensity, 3.0 cm from the reaction vessel) while stirring. After the completion of the reaction, the crude obtained upon solvent removal under reduced pressure was purified by flash column chromatography using either petroleum ether and ethyl acetate (PE/EtOAc) or petroleum ether, dichloromethane, and ethyl acetate (PE/DCM/EtOAc) as the eluent system to afford the pure product. All compounds were characterized via HRMS and ^1^H NMR, ^13^C{1H} NMR, and ^19^F NMR spectroscopies.

#### Methyl 4-((Dimethyl(oxo)-λ^6^-sulfaneylidene)amino)-2,3,5,6-tetrafluorobenzoate **9**

The compound was obtained according to general procedure B using azide **1a** (75 mg, 0.3 mmol, 1 equiv) and DMSO (32 μL, 0.45 mmol, 1.5 equiv). The reaction was completed after 2 h of the reaction. The pure product was obtained after flash column chromatography (eluent: PE/EtOAc, 2:1 → 1:1) (rf: 0.3, eluent PE/EtOAc 1:1) as a white precipitate (70 mg, 78%). mp: 129–130 °C ^1^H NMR (CDCl_3_, 400 MHz): δ 3.94 (s, 3H, OCH_3_), and 3.29 (s, 6H, CH_3_); ^13^C{^1^H} NMR (CDCl_3_, 125 MHz): δ 160.9, 146–0 (dm, *J* = 256 Hz), 142.1 (dm, *J* = 243 Hz), 127.6, 104.7, 53.0, and 44.8; ^19^F NMR (CDCl_3_, 376 MHz): δ −140.8 (m, 2F) and −149.5 (m, 2F). HRMS (ESI-TOF) *m*/*z*: [M + H]^+^ calcd for C_10_H_10_F_4_NO_3_S, 300.0312; found, 300.0312.

#### Methyl 2,3,5,6-Tetrafluoro-4-((methyl(oxo)(phenyl)-λ^6^-sulfaneylidene)amino)benzoate *rac*-**10**

The compound was obtained according to general procedure B using azide **1a** (64 mg, 0.26 mmol, 1 equiv) and phenyl vinyl SO (51 mg, 0.36 mmol, 1.4 equiv). The reaction was completed after 4 h of the reaction. The pure product was obtained after flash column chromatography (eluent: PE/DCM/EtOAc, 10:1:1 → 6:1:1) (rf: 0.36, eluent: 6:1:1) as a white precipitate (57 mg, 61%). mp: 140–142 °C. ^1^H NMR (CDCl_3_, 400 MHz): δ 8.03–7.93 (m, 2H), 7.72–7.64 (m, 1H), 7.63–7.54 (m, 2H), 3.90 (s, 3H), and 3.36 (s, 3H); ^13^C{^1^H} NMR (CDCl_3_, 125 MHz): δ 161.0, 145.7 (dm, *J* = 255.4 Hz), 141.7 (dm, *J* = 244.6 Hz), 139.6, 134.1, 129.9, 128.0, 127.7, 104.2, 52.9, and 47.3; ^19^F NMR (CDCl_3_, 376 MHz): δ −141.0 (m, 2F) and −148.6 (m, 2F). HRMS (ESI-TOF) *m*/*z*: [M + H]^+^ calcd for C_15_H_12_F_4_NO_3_S, 362.0468; found, 362.0466.

#### Methyl 2,3,5,6-Tetrafluoro-4-((methyl(oxo)(vinyl)-λ^6^-sulfaneylidene)amino)benzoate *rac*-**11**

The compound was obtained according to general procedure B using azide **1a** (75 mg, 0.3 mmol, 1 equiv) and phenyl vinyl SO (*rac*) (46 μL, 0.42 mmol, 1.4 equiv). The reaction was completed after 4 h of the reaction. The pure product was obtained after flash column chromatography (eluent: PE/DCM/EtOAc, 10:1:1 → 6:1:1) (rf: 0.4, eluent: 6:1:1) as an off-white precipitate (63 mg, 56%). mp: 106–107 °C. ^1^H NMR (CDCl_3_, 400 MHz): δ 8.06–7.91 (m, 2H), 7.72–7.63 (m, 1H), 7.60–7.53 (m, 2H), 6.74 (dd, *J* = 16.2, 9.4 Hz, 1H), 6.56 (d, *J* = 16.5 Hz, 1H), 6.14 (d, *J* = 9.3 Hz, 1H), and 3.91 (s, 3H); ^13^C{^1^H} NMR (CDCl_3_, 125 MHz): δ 161.0, 145.7 (dm, *J* = 255.5 Hz), 142.0 (dm, *J* = 244.8 Hz), 138.6, 138.3, 134.1, 129.8, 129.4, 128.4, 127.8, 104.6, and 52.9; ^19^F NMR (CDCl_3_, 376 MHz): δ −141.0 (m, 2F) and −148.1 (m, 2F). HRMS (ESI-TOF) *m*/*z*: [M + H]^+^ calcd for C_16_H_12_F_4_NO_3_S, 374.0468; found, 374.0467.

#### Methyl 2,3,5,6-Tetrafluoro-4-(((2-methoxy-2-oxoethyl)(oxo)(phenyl)-λ^6^-sulfaneylidene)-amino)benzoate *rac*-**12**

The compound was obtained according to general procedure B using azide **1a** (74 mg, 0.3 mmol, 1 equiv) and methyl-phenylsulfinylacetate (*rac*) (91 mg, 0.45 mmol, 1.5 equiv). The reaction was completed after 2 h of the reaction. The pure product was obtained after flash column chromatography (eluent: PE/DCM/EtOAc, 10:1:1 → 6:1:1) (rf: 0.14, eluent: 6:1:1) as an off-white precipitate (58 mg, 47%). mp: 97–98 °C. ^1^H NMR (CDCl_3_, 500 MHz): δ 8.05–7.96 (m, 2H), 7.83–7.67 (m, 1H), 7.66–7.58 (m, 2H), 4.38 (s, 2H), 3.92 (s, 3H), and 3.70 (s, 3H); ^13^C{^1^H} NMR (CDCl_3_, 125 MHz): δ 162.4, 160.9, 145.9 (dm, *J* = 255.8 Hz), 141.8 (dm, *J* = 244.9 Hz), 137.9, 134.7, 129.8, 128.8, 127.2, 104.8, 62.8, 53.3, and 53.0; ^19^F NMR (CDCl_3_, 376 MHz): δ −140.8 (m, 2F) and −148.3 (m, 2F). HRMS (ESI-TOF) *m*/*z*: [M + Na]^+^ calcd for C_17_H_13_F_4_NO_5_SNa, 442.0344; found, 442.0341.

#### Methyl (*R*)-2,3,5,6-Tetrafluoro-4-((methyl(oxo)(*p*-tolyl)-λ^6^-sulfaneylidene)amino)benzoate (*R*)-**13**

The compound was obtained according to general procedure B using azide **1a** (74 mg, 0.3 mmol, 1 equiv) and (*R*)-methyl *p*-tolyl SO (64 mg, 0.4 mmol, 1.4 equiv). The reaction was completed after 2 h of the reaction. The pure product was obtained after flash column chromatography (eluent: PE/DCM/EtOAc, 6:1:1) (rf: 0.44, eluent: 4:1:1) as a white precipitate (74 mg, 66%). mp: 86–87 °C. ^1^H NMR (CDCl_3_, 400 MHz): δ 7.83 (d, *J* = 8.2 Hz, 2H), 7.37 (d, *J* = 8.2 Hz, 2H), 3.90 (s, 3H), 3.34 (s, 3H), and 2.44 (s, 3H); ^13^C{^1^H} NMR (CDCl_3_, 125 MHz): δ 161.0, 145.9 (dm, *J* = 255.3 Hz), 145.2, 141.9 (dm, *J* = 244.7 Hz), 136.4, 130.6, 128.3, 127.7, 104.1, 52.9, 47.4, and 21.7; ^19^F NMR (CDCl_3_, 376 MHz): δ −141.1 (m, 2F) and −148.1 (m, 2F). HRMS (ESI-TOF) *m*/*z*: [M + H]^+^ calcd for C_16_H_14_F_4_NO_3_S, 376.0625; found, 376.0626. [α]_D_^27^ – 113 (*c* 0.4, CHCl_3_).

#### Methyl 4-(((4-Chlorobenzyl)(4-chlorophenyl)(oxo)-λ^6^-sulfaneylidene)amino)-2,3,5,6-tetrafluorobenzoate *rac*-**14**

The compound was obtained according to general procedure B using azide **1a** (56 mg, 0.2 mmol, 1 equiv) and chlorbensid SO (85 mg, 0.3 mmol, 1.3 equiv). The reaction was run in ethyl acetate (deg) and was completed after 2 h of the reaction. The pure product was obtained after flash column chromatography (eluent: PE/DCM, 1:1 → 1:2) (rf: 0.15, eluent: PE/DCM, 1:1) as a colorless solid (60 mg, 53%). mp: 109–110 °C. ^1^H NMR (CDCl_3_, 500 MHz): δ 7.61–7.49 (m, 2H), 7.48–7.41 (m, 2H), 7.33–7.22 (m, 2H), 7.16–7.05 (m, 2H), 4.79–4.34 (m, 2H), and 3.90 (s, 3H); ^13^C{^1^H} NMR (CDCl_3_, 125 MHz): δ 160.9, 145.9 (dm, *J* = 255.7 Hz), 141.7 (dm, *J* = 244.6 Hz), 141.18, 136.0, 135.4, 132.8, 130.2, 129.9, 129.1, 127.7, 125.8, 104.3, 64.6, and 52.9; ^19^F NMR (CDCl_3_, 376 MHz): δ −140.8 (m, 2F) and −148.4 (m, 2F). HRMS (ESI-TOF) *m*/*z*: [M + Na]^+^ calcd for C_21_H_13_Cl_2_F_4_NO_3_SNa, 527.9822; found, 527.9823.

#### (2*S*)-2-((*tert*-Butoxycarbonyl)amino)-4-(*S*-methyl-*N*-(2,3,5,6-tetrafluoro-4-(methoxycarbonyl)-phenyl)sulfonimidoyl)butanoic Acid **15**

The compound was obtained according to general procedure B using azide **1a** (75 mg, 0.3 mmol, 1 equiv) and l-methionine SO *N*-Boc protected (120 mg, 0.45 mmol, 1.5 equiv). The reaction was completed after 1 h of the reaction. The pure product was obtained after flash column chromatography (eluent: 2.5% MeOH in DCM + 0.25% formic acid → 5.0% MeOH in DCM + 0.25% formic acid) (rf: 0.25, eluent: 5.0% MeOH in DCM + 0.5% formic acid) as a pale-yellow precipitate (72 mg, 49%). mp: 111–112 °C. ^1^H NMR (CDCl_3_, 500 MHz): δ 9.97 (br, 1H, CO_2_H), 7.05–5.52 (br, 1H, NH), 4.45–4.40 (s, 1H, CH), 3.92 (s, 3H, OCH_3_), 3.57–3.66 (m, 2H, CH_2_), 3.21 (s, 3H, *S*-CH_3_), 2.56–2.33 (m, 2H, CH_2_), and 1.50–1.42 (s, 9H, *t*-Bu); ^13^C{^1^H} NMR (CDCl_3_, 125 MHz): δ 174.1, 161.0, 156.9, 155.9, 145.9 (dm, *J* = 256 Hz), 142.0 (dm, *J* = 240 Hz), 127.5, 104.6, 83.21, 81.1, 53.3, 53.0, 52.0, 42.5, 28.3, and 25.9; ^19^F NMR (CDCl_3_, 376 MHz): δ −140.7 (m, 2F) and −149.2 (m, 2F). HRMS (ESI-TOF) *m*/*z*: [M + Na]^+^ calcd for C_18_H_22_F_4_N_2_O_7_SNa, 509.0976; found, 509.0973.

### General Procedure C for the Condensation Reaction

To a dry round-bottom flask equipped with a magnetic stir bar, a reflux condenser and 4 Å molecular sieves (MSs), SIA (1 equiv, 0.8 mmol, 0.1 M), aldehyde (2 equiv), pyrrolidine (0.08 mmol, 0.1 equiv), and anhydrous CH_2_Cl_2_ (8 mL) were added. The reaction was refluxed under an inert atmosphere (N_2_). After the completion of the reaction, the crude obtained upon solvent removal under reduced pressure was purified by flash column chromatography using petroleum ether, dichloromethane, and ethyl acetate (PE/EtOAc) as the eluent system to afford the pure product. All compounds were characterized via HRMS and ^1^H NMR, ^13^C{1H} NMR, and ^19^F NMR spectroscopies.

#### (*S*,*E*)-*N*-(2,2-Dimethylpropylidene)-2-methyl-*N*′-(perfluoropyridin-4-yl)propane-2-sulfonimidamide (*R*)-**16a**

The compound was obtained according to general procedure C using compound (*R*)-**3** (1 equiv) and pivaldehyde (2.0 equiv). The reaction was completed after 40 h of the reaction. The pure product was obtained after flash column chromatography (eluent: PE/EtOAc 20:1) as a white precipitate (125 mg, 90%). mp: 88 °C. ^1^H NMR (CDCl_3_, 500 MHz): δ 8.50 (s, 1H, imine), 1.54 (s, 9H, *t*-Bu), and 1.42 (s, 9H, *t*-Bu); ^13^C{^1^H} NMR (CDCl_3_, 125 MHz): δ 189.4, 144.3 (dm, *J* = 240 Hz), 137.1 (dm, *J* = 252 Hz), 136.7, 62.0, 38.9, 26.1, and 23.8; ^19^F NMR (CDCl_3_, 376 MHz): δ −93.8 (m, 2F) and −152.0 (m, 2F). HRMS (ESI-TOF) *m*/*z*: [M + Na]^+^ calcd for C_14_H_19_F_4_N_3_OSNa, 376.1077; found, 376.1078. [α]_D_^31^ – 64 (*c* 0.2, CHCl_3_).

#### (*S*,*E*)-*N*-Benzylidene-2-methyl-*N*′-(perfluoropyridin-4-yl)propane-2-sulfonimidamide (*R*)-**16b**

The compound was obtained according to general procedure C using compound (*R*)-**3** (1 equiv) and benzaldehyde (2.0 equiv). The reaction was completed after 40 h of the reaction. The pure product was obtained after flash column chromatography (eluent: PE/EtOAc 20:1) as a white crystal (60 mg, 78%). mp: 68–70 °C. ^1^H NMR (CDCl_3_, 500 MHz): δ 9.06 (s, 1H, imine), 7.99 (m, 2H), 7.69 (m, 1H), 7.55 (m, 2H), and 1.61 (s, 9H, *t*-Bu); ^13^C{^1^H} NMR (CDCl_3_, 125 MHz): δ 174.6, 144.20 (dm, *J* = 240 Hz), 137.1 (dm, *J* = 252 Hz), 136.7, 135.7, 132.2, 131.6, 129.5, 62.5, and 23.9; ^19^F NMR (CDCl_3_, 376 MHz): δ −93.9 (m, 2F) and −152.0 (m, 2F). HRMS (ESI-TOF) *m*/*z*: [M + Na]^+^ calcd for C_16_H_15_F_4_N_3_OSNa, 396.0764; found, 396.0768. [α]_D_^31^ – 404 (*c* 0.3, CHCl_3_).

#### (*S*,*E*)-*N*-(4-Chlorobenzylidene)-2-methyl-*N*′-(perfluoropyridin-4-yl)propane-2-sulfonimidamide (*R*)-**16c**

The compound was obtained according to general procedure C using compound (*R*)-**3** (1 equiv) and 4-chloro benzaldehyde (2.0 equiv). The reaction was completed after 40 h of the reaction. The pure product was obtained after flash column chromatography (eluent: PE/EtOAc 20:1) as white crystals (254 mg, 78%). mp: 99–100 °C. ^1^H NMR (CDCl_3_, 500 MHz): δ 9.02 (s, 1H, imine), 7.93 (d, *J* = 8.5, 2H), 7.54 (d, *J* = 8.5, 2H), and 1.60 (s, 9H, *t*-Bu); ^13^C{^1^H} NMR (CDCl_3_, 125 MHz): δ 173.1, 144.1 (dm, *J* = 241 Hz), 142.4, 137.1 (dm, *J* = 252 Hz), 136.5, 132.7, 130.7, 130.1, 62.6, and 24.0; ^19^F NMR (CDCl_3_, 376 MHz): δ −93.77 (m, 2F) and −151.89 (m, 2F). HRMS (ESI-TOF) *m*/*z*: [M + Na]^+^ calcd for C_16_H_14_ClF_4_N_3_OSNa, 430.0375; found, 430.0376. [α]_D_^31^ – 194 (*c* 0.2, CHCl_3_).

### General Procedure D for the Solvent Screening of Grignard Addition Reactions

To a dry Biotage microwave vial equipped with a magnetic stir bar, a 0.5 mL solution of SIA-imine (1 equiv, 0.05 mmol, 0.1 M) was added. The solution was allowed to reach −78 °C in an acetone/dry ice bath, and 47 μL of a solution (3.0 M in Et_2_O) of phenyl magnesium bromine was added drop-wise. The reaction mixture was stirred for 6 h. The crude reaction mixture was sampled, quenched with sat. aq. sol. of NH_4_Cl, and analyzed via ^1^H NMR to determine the conversion and the dr.

### General Procedure E for Grignard Addition Reactions

To a dry Biotage microwave vial equipped with a magnetic stir bar, a 0.5 mL solution of SIA-imine (1 equiv, 0.05 mmol, 0.1 M) was added. The solution was allowed to reach −78 °C in an acetone/dry ice bath and the Grignard reagent (0.125 mmol, 2.5 equiv) was added dropwise to the solution. The reaction mixture was stirred at −78 °C for 6 h and then let reach r.t. overnight. The crude reaction mixture was quenched with sat. aq. sol. of NH_4_Cl (2 mL) and extracted with EtOAc (4 × 1 mL). The organic phases were combined, dried over Na_2_SO_4_, and filtered, and the solvent was removed via rotary evaporation in vacuo. The yield of the reaction was obtained via ^1^H NMR using *tert*-butyl methyl ether as the internal standard. The dr was obtained via ^1^H NMR analysis.

#### (*S*)-*N*-(1-(3-Methoxyphenyl)-2,2-dimethylpropyl)-2-methyl-*N*′-(perfluoropyridin-4-yl)propane-2-sulfonimidamide **17**

The compound was obtained according to general procedure E using imine (*R*)-**16a** (1 equiv, 0.3 mmol, 100 mg) and a 1.0 M solution of 3-methoxyphenylmagnesium bromide in THF (2.5 equiv). The crude reaction mixture was quenched with sat. aq. sol. of NH_4_Cl (10 mL) and extracted with EtOAc (4 × 8 mL). The organic phases were combined, washed with H_2_O, dried over Na_2_SO_4_, and filtered, and the solvent was removed via rotary evaporation in vacuo. The pure product was obtained without further purification as colorless powder (124 mg, 95% yield, 95:5 dr). HPLC (Kromasil 5-CelluCoat RP, 0.46 cm × 25 cm, *n*-hexane/isopropanol = 98/2, flow rate = 1.0 mL/min, λ = 220 nm) *t*_R_ = 14.5 min (major), 22.1 min (minor). mp: 152–153 °C. ^1^H NMR (500 MHz, CDCl_3_): δ 7.06 (t, *J* = 7.9 Hz, 1H), 6.66 (m, 1H), 6.59 (m, 1H), 6.49 (m, 1H), 4.21 (d, *J* = 9.9 Hz, 1H, NH), 4.13 (d, *J* = 9.8 Hz, 1H, CH), 3.73 (s, 3Hm OCH_3_), 1.52 (s, 9H, *t*-Bu), and 0.94 (s, 9H, *t*-Bu); ^13^C{^1^H} NMR (125 MHz, CDCl_3_): δ 159.1, 143.8 (dm, *J* = 243 Hz), 142.6, 137.9 (dm, *J* = 253 Hz), 136.1, 128.8, 120.2, 114.4, 111.5, 67.6, 64.1, 55.1, 35.8, 27.5, and 24.6. ^19^F NMR (CDCl_3_, 376 MHz): δ −93.6 (m, 2F) and −151.2 (m, 2F). HRMS (ESI-TOF) *m*/*z*: [M + Na]^+^ calcd for C_21_H_27_F_4_N_3_O_2_SNa, 484.1653; found, 484.1654. [α]_D_^31^ – 20 (*c* 0.2, CHCl_3_).

#### 1-(3-Methoxyphenyl)-2,2-dimethylpropan-1-amine **18**

Compound **17** (1 equiv, 0.09 mmol, 40 mg) and anisole (20 equiv) were introduced into a round-bottom flask, equipped with a magnetic stirrer, containing 8 mL of dichloromethane. The reaction mixture was cooled down to 0–5 °C (ice bath), and a 4 mL solution of triflic acid in dichloromethane (0.2 M) was added dropwise. After the addition, the reaction was let reach room temperature. After the completion of the reaction (2 h), the crude mixture was quenched with aqueous NaOH (2 M, 10 mL) and extracted with dichloromethane (3 × 10 mL). The reunited organic phase was dried over Na_2_SO_4_ and filtered, and the solvent was removed via rotary evaporation in vacuo. The pure product was obtained via preparative-TLC (eluent: 5% MeOH in DCM) (rf: 0.2, eluent: 5% MeOH in DCM) as a colorless liquid (15 mg, 90%, 95:5 dr). HPLC (ReproSil Chiral-NR, 0.46 cm × 25 cm, *n*-hexane/isopropanol = 70/30, flow rate = 1.0 mL/min, λ = 220 nm) *t*_R_ = 6.5 min (minor), 8.4 min (major). mp: 152–153 °C. ^1^H NMR (500 MHz, CDCl_3_): δ 7.20 (m, 1H), 6.85 (m, 2H), 6.78 (m, 1H), 3.80 (s, 3H, OCH_3_), 2.94 (br, 2H, NH_2_), and 0.92 (s, 9H, *t*-Bu); ^13^C{^1^H} NMR (125 MHz, CDCl_3_): δ 159.2, 144.8, 128.6, 121.0, 114.3, 112.2, 65.4, 55.3, 35.1, and 26.7. HRMS (ESI-TOF) *m*/*z*: [M + H]^+^ calcd for C_12_H_20_NO, 194.1539; found, 194.1541. [α]_D_^30^ – 2.4 (*c* 0.5, methanol).

## Supporting Information Available

Supporting Information includes spectroscopic characterizations (^1^H, ^13^C, and ^19^F NMR), HRMS, and HPLC chromatograms (PDF)

## References

[ref1] Nandi G. C.; Arvidsson P. I. Sulfonimidamides: Synthesis and Applications in Preparative Organic Chemistry. Adv. Synth. Catal. 2018, 360, 2976–3001. 10.1002/adsc.201800273.

[ref2] Chinthakindi P. K.; Naicker T.; Thota N.; Govender T.; Kruger H. G.; Arvidsson P. I. Sulfonimidamides in Medicinal and Agricultural Chemistry. Angew. Chem., Int. Ed. 2017, 56, 4100–4109. 10.1002/anie.201610456. 27958674

[ref3] Sehgelmeble F.; Janson J.; Ray C.; Rosqvist S.; Gustavsson S.; Nilsson L. I.; Minidis A.; Holenz J.; Rotticci D.; Lundkvist J.; Arvidsson P. I. Sulfonimidamides as Sulfonamides Bioisosteres: Rational Evaluation through Synthetic, in Vitro, and in Vivo Studies with γ-Secretase Inhibitors. ChemMedChem 2012, 7, 396–399. 10.1002/cmdc.201200014. 22307979

[ref4] Izzo F.; Schäfer M.; Lienau P.; Ganzer U.; Stockman R.; Lücking U. Exploration of Novel Chemical Space: Synthesis and in vitro Evaluation of N-Functionalized Tertiary Sulfonimidamides. Chem.—Eur. J. 2018, 24, 9295–9304. 10.1002/chem.201801557. 29726583 PMC6055826

[ref5] Lücking U. Neglected sulfur(vi) pharmacophores in drug discovery: exploration of novel chemical space by the interplay of drug design and method development. Org. Chem. Front. 2019, 6, 1319–1324. 10.1039/c8qo01233d.

[ref6] Lücking U. Sulfoximines: A Neglected Opportunity in Medicinal Chemistry. Angew. Chem., Int. Ed. 2013, 52, 9399–9408. 10.1002/anie.201302209. 23934828

[ref7] Sirvent J. A.; Lücking U. Novel Pieces for the Emerging Picture of Sulfoximines in Drug Discovery: Synthesis and Evaluation of Sulfoximine Analogues of Marketed Drugs and Advanced Clinical Candidates. ChemMedChem 2017, 12, 487–501. 10.1002/cmdc.201700044. 28221724 PMC5485063

[ref8] Min A.; Im S.-A.; Jang H.; Kim S.; Lee M.; Kim D. K.; Yang Y.; Kim H.-J.; Lee K.-H.; Kim J. W.; Kim T.-Y.; Oh D.-Y.; Brown J.; Lau A.; O’Connor M. J.; Bang Y.-J. AZD6738, A Novel Oral Inhibitor of ATR, Induces Synthetic Lethality with ATM Deficiency in Gastric Cancer Cells. Mol. Cancer Ther. 2017, 16, 566–577. 10.1158/1535-7163.mct-16-0378. 28138034

[ref9] Foote K. M.; Nissink J. W. M.; McGuire T.; Turner P.; Guichard S.; Yates J. W. T.; Lau A.; Blades K.; Heathcote D.; Odedra R.; Wilkinson G.; Wilson Z.; Wood C. M.; Jewsbury P. J. Discovery and Characterization of AZD6738, a Potent Inhibitor of Ataxia Telangiectasia Mutated and Rad3 Related (ATR) Kinase with Application as an Anticancer Agent. J. Med. Chem. 2018, 61, 9889–9907. 10.1021/acs.jmedchem.8b01187. 30346772

[ref10] Vendetti F. P.; Lau A.; Schamus S.; Conrads T. P.; O’Connor M. J.; Bakkenist C. J. The orally active and bioavailable ATR kinase inhibitor AZD6738 potentiates the anti-tumor effects of cisplatin to resolve ATM-deficient non-small cell lung cancer in vivo. Oncotarget 2015, 6, 44289–44305. 10.18632/oncotarget.6247. 26517239 PMC4792557

[ref11] Lücking U.; Jautelat R.; Krüger M.; Brumby T.; Lienau P.; Schäfer M.; Briem H.; Schulze J.; Hillisch A.; Reichel A.; Wengner A. M.; Siemeister G. The Lab Oddity Prevails: Discovery of Pan-CDK Inhibitor (R)-S-Cyclopropyl-S-(4-{[4-{[(1R,2R)-2-hydroxy-1-methylpropyl]oxy}-5-(trifluoromethyl)pyrimidin-2-yl]amino}phenyl)sulfoximide (BAY1000394)for the Treatment of Cancer. ChemMedChem 2013, 8, 1067–1085. 10.1002/cmdc.201300096. 23671017

[ref12] Lücking U.; Scholz A.; Lienau P.; Siemeister G.; Kosemund D.; Bohlmann R.; Briem H.; Terebesi I.; Meyer K.; et al. Identification of Atuveciclib (BAY1143572),the First Highly Selective ,Clinical PTEFb /CDK9Inhibitor for the Treatment of Cancer. ChemMedChem 2017, 12, 1776–1793. 10.1002/cmdc.201700447. 28961375 PMC5698704

[ref13] Wojaczyńska E.; Wojaczyński J. Modern Stereoselective Synthesis of Chiral Sulfinyl Compounds. Chem. Rev. 2020, 120, 4578–4611. 10.1021/acs.chemrev.0c00002. 32347719 PMC7588045

[ref14] Takei H.; Itaru W.; Teruaki M. The Preparation of Iminosulfonic Acid Derivatives by Means of Sulfinamides and N-Bromosuccinimide. Bull. Chem. Soc. Jpn. 1965, 38, 1989–1993. 10.1246/bcsj.38.1989.

[ref15] Jonsson E. U.; Bacon C. C.; Johnson C. R. Chemistry of sulfoxides and related compounds. XXXII. Preparation of sulfonimidoyl chlorides by chlorination of sulfinamides. J. Am. Chem. Soc. 1971, 93, 5306–5308. 10.1021/ja00749a084.

[ref16] Chen Y.; Gibson J. A convenient synthetic route to sulfonimidamides from sulfonamides. RSC Adv. 2015, 5, 4171–4174. 10.1039/c4ra14056g.

[ref17] Davies T. Q.; Hall A.; Willis M. C. One-Pot, Three-Component Sulfonimidamide Synthesis Exploiting the Sulfinylamine Reagent N-Sulfinyltritylamine, TrNSO. Angew. Chem., Int. Ed. 2017, 56, 14937–14941. 10.1002/anie.201708590. 28929561

[ref18] Liu F.; Wang H.; Li S.; Bare G. A. L.; Chen X.; Wang C.; Moses J. E.; Wu P.; Sharpless K. B. Biocompatible SuFEx Click Chemistry: Thionyl Tetrafluoride (SOF4)-Derived Connective Hubs for Bioconjugation to DNA and Proteins. Angew. Chem., Int. Ed. 2019, 58, 8029–8033. 10.1002/anie.201902489. PMC6546515 30998840

[ref19] Greed S.; Briggs E. L.; Idiris F. I. M.; White A. J. P.; Lücking U.; Bull J. A. Synthesis of Highly Enantioenriched Sulfonimidoyl Fluorides and Sulfonimidamides by Stereospecific Sulfur–Fluorine Exchange (SuFEx) Reaction. Chem.—Eur. J. 2020, 26, 12533–12538. 10.1002/chem.202002265. 32428384 PMC7590120

[ref20] Yu H.; Li Z.; Bolm C. Copper-Catalyzed Transsulfinamidation of Sulfinamides as a Key Step in the Preparation of Sulfonamides and Sulfonimidamides. Angew. Chem., Int. Ed. 2018, 57, 15602–15605. 10.1002/anie.201810548. 30290042

[ref21] Wen J.; Cheng H.; Dong S.; Bolm C. Copper-Catalyzed S–C/S–N Bond Interconversions. Chem.—Eur. J. 2016, 22, 5547–5550. 10.1002/chem.201600661. 26892735

[ref22] Izzo F.; Schäfer M.; Stockman R.; Lücking U. A New, Practical One-Pot Synthesis of Unprotected Sulfonimidamides by Transfer of Electrophilic NH to Sulfinamides. Chem.—Eur. J. 2017, 23, 15189–15193. 10.1002/chem.201703272. 28833686 PMC5698725

[ref23] Zheng W.; Chen X.; Chen F.; He Z.; Zeng Q. Syntheses and Transformations of Sulfoximines. Chem. Rec. 2020, 21, 396–416. 10.1002/tcr.202000134. 33369096

[ref24] Bach T.; Körber C. The Preparation of N-tert-Butyloxycarbonyl-(Boc-)Protected Sulfoximines and Sulfimines by an Iron(II)-Mediated Nitrene Transfer from BocN3 to Sulfoxides and Sulfides. Eur. J. Org. Chem. 1999, 1999, 1033–1039. 10.1002/(sici)1099-0690(199905)1999:5<1033::aid-ejoc1033>3.0.co;2-1.

[ref25] Okamura H.; Bolm C. Rhodium-Catalyzed Imination of Sulfoxides and Sulfides:Efficient Preparation of N -UnsubstitutedSulfoximines and Sulfilimines. Org. Lett. 2004, 6, 1305–1307. 10.1021/ol049715n. 15070323

[ref26] Cho G. Y.; Bolm C. Silver-Catalyzed Imination of Sulfoxides and Sulfides. Org. Lett. 2005, 7, 4983–4985. 10.1021/ol0519442. 16235938

[ref27] Mancheño O. G.; Bolm C. Iron-Catalyzed Imination of Sulfoxides and Sulfides. Org. Lett. 2006, 8, 2349–2352. 10.1021/ol060640s. 16706523

[ref28] Mancheño O. G.; Dallimore J.; Plant A.; Bolm C. Iron(II) Triflate as an Efficient Catalyst for the Imination of Sulfoxides. Org. Lett. 2009, 11, 2429–2432. 10.1021/ol900660x. 19473047

[ref29] Ochiai M.; Naito M.; Miyamoto K.; Hayashi S.; Nakanishi W. Imination of Sulfides and Sulfoxides with Sulfonylimino-λ3-Bromane under Mild, Metal-Free Conditions. Chem.—Eur. J. 2010, 16, 8713–8718. 10.1002/chem.201000759. 20572186

[ref30] Zenzola M.; Doran R.; Degennaro L.; Luisi R.; Bull J. A. Transfer of Electrophilic NH Using Convenient Sources of Ammonia: Direct Synthesis of NH Sulfoximines from Sulfoxides. Angew. Chem., Int. Ed. 2016, 55, 7203–7207. 10.1002/anie.201602320. PMC5074267 27126053

[ref31] Bull J.; Luisi R.; Degennaro L. Straightforward Strategies for the Preparation of NH-Sulfoximines: A Serendipitous Story. Synlett 2017, 28, 2525–2538. 10.1055/s-0036-1590874.

[ref32] Degennaro L.; Tota A.; De Angelis S.; Andresini M.; Cardellicchio C.; Capozzi M. A.; Romanazzi G.; Luisi R. A Convenient, Mild, and Green Synthesis of NH-Sulfoximines in Flow Reactors. Eur. J. Org. Chem. 2017, 2017, 6486–6490. 10.1002/ejoc.201700850.

[ref33] Sun Y.; Cramer N. Enantioselective Synthesis of Chiral-at-Sulfur 1,2-Benzothiazines by CpxRhIII-Catalyzed C–H Functionalization of Sulfoximines. Angew. Chem., Int. Ed. 2018, 57, 15539–15543. 10.1002/anie.201810887. 30300950

[ref34] Tang Y.; Miller S. J. Catalytic Enantioselective Synthesis of Pyridyl Sulfoximines. J. Am. Chem. Soc. 2021, 143, 9230–9235. 10.1021/jacs.1c04431. 34124892 PMC8262370

[ref35] Aota Y.; Kano T.; Maruoka K. Asymmetric Synthesis of Chiral Sulfoximines through the S-Alkylation of Sulfinamides. Angew. Chem., Int. Ed. 2019, 58, 17661–17665. 10.1002/anie.201911021. 31568618

[ref36] Müller K.; Faeh C.; Diederich F. Fluorine in Pharmaceuticals: Looking Beyond Intuition. Science 2007, 317, 1881–1886. 10.1126/science.1131943. 17901324

[ref37] Wang J.; Sánchez-Roselló M.; Aceña J. L.; del Pozo C.; Sorochinsky A. E.; Fustero S.; Soloshonok V. A.; Liu H. Fluorine in Pharmaceutical Industry: Fluorine-Containing Drugs Introduced to the Market in the Last Decade (2001–2011). Chem. Rev. 2014, 114, 2432–2506. 10.1021/cr4002879. 24299176

[ref38] Böhm H.-J.; Banner D.; Bendels S.; Kansy M.; Kuhn B.; Müller K.; Obst-Sander U.; Stahl M. Fluorine in Medicinal Chemistry. ChemBioChem 2004, 5, 637–643. 10.1002/cbic.200301023. 15122635

[ref39] Shen X.; Hu J. Fluorinated Sulfoximines: Preparation, Reactions and Applications. Eur. J. Org. Chem. 2014, 2014, 4437–4451. 10.1002/ejoc.201402086.

[ref40] Bizet V.; Kowalczyk R.; Bolm C. Fluorinated sulfoximines: syntheses, properties and applications. Chem. Soc. Rev. 2014, 43, 2426–2438. 10.1039/c3cs60427f. 24549291

[ref41] Barthelemy A.-L.; Magnier E. Recent trends in perfluorinated sulfoximines. C. R. Chim. 2018, 21, 711–722. 10.1016/j.crci.2018.01.004.

[ref42] Yagupolskii L. M. Aromatic compounds with new fluorine-containing substituents. J. Fluorine Chem. 1985, 29, 6 10.1016/s0022-1139(00)83243-1.

[ref43] Kirsch P.; Lenges M.; Kühne D.; Wanczek K.-P. Synthesis and Structural Characterization of Highly Fluorinated Sulfimides and Sulfoximides as Functional Building Blocks for Materials Science. Eur. J. Org. Chem. 2005, 2005, 797–802. 10.1002/ejoc.200400702.

[ref44] Bohnen C.; Bolm C. N-Trifluoromethylthiolated Sulfoximines. Org. Lett. 2015, 17, 3011–3013. 10.1021/acs.orglett.5b01384. 26029817

[ref45] Nishimura N.; Norman M. H.; Liu L.; Yang K. C.; Ashton K. S.; Bartberger M. D.; Chmait S.; Chen J.; Cupples R.; et al. Small Molecule Disruptors of the Glucokinase–Glucokinase Regulatory Protein Interaction: 3. Structure–Activity Relationships within the Aryl Carbinol Region of the N-Arylsulfonamido-N′-arylpiperazine Series. J. Med. Chem. 2014, 57, 3094–3116. 10.1021/jm5000497. 24611879

[ref46] Teng F.; Cheng J.; Bolm C. Silver-Mediated N-Trifluoromethylation of Sulfoximines. Org. Lett. 2015, 17, 3166–3169. 10.1021/acs.orglett.5b01537. 26057854

[ref47] Miyasaka M.; Hirano K.; Satoh T.; Kowalczyk R.; Bolm C.; Miura M. Copper-Catalyzed Direct Sulfoximination of Azoles and Polyfluoroarenes under Ambient Conditions. Org. Lett. 2011, 13, 359–361. 10.1021/ol102844q. 21174416

[ref48] Schumacher C.; Fergen H.; Puttreddy R.; Truong K.-N.; Rinesch T.; Rissanen K.; Bolm C. N-(2,3,5,6-Tetrafluoropyridyl)sulfoximines: synthesis, X-ray crystallography, and halogen bonding. Org. Chem. Front. 2020, 7, 3896–3906. 10.1039/d0qo01139h.

[ref49] Gritsan N. P.; Platz M. S. Kinetics, spectroscopy, and computational chemistry of arylnitrenes. Chem. Rev. 2006, 106, 3844–3867. 10.1021/cr040055+. 16967923

[ref50] Banks R. E.; Sparkes G. R. Studies in azide chemistry. Part V. Synthesis of 4-azido-2, 3, 5, 6-tetrafluoro-, 4-azido-3-chloro-2, 5, 6-trifluoro-, and 4-azido-3, 5-dichloro-2, 6-difluoro-pyridine, and some thermal reactions of the tetrafluoro-compound. J. Chem. Soc., Perkin Trans. 1 1972, 2964–2970. 10.1039/p19720002964.

[ref51] Young M. J. T.; Platz M. S. Polyfluorinated aryl azides as photoaffinity labelling reagents; the room temperature CH insertion reactions of singlet pentafluorophenyl nitrene with alkanes. Tetrahedron Lett. 1989, 30, 2199–2202. 10.1016/s0040-4039(00)99647-3.

[ref52] Gritsan N. P.; Gudmundsdóttir A. D.; Tigelaar D.; Zhu Z.; Karney W. L.; Hadad C. M.; Platz M. S. A laser flash photolysis and quantum chemical study of the fluorinated derivatives of singlet phenylnitrene. J. Am. Chem. Soc. 2001, 123, 1951–1962. 10.1021/ja9944305. 11456816

[ref53] Blomkvist B.; Dinér P. HBF4·DEE-catalyzed formation of sulfinyl imines: Synthesis and mechanistic studies. Tetrahedron Lett. 2018, 59, 1249–1253. 10.1016/j.tetlet.2018.02.051.

[ref54] Blomkvist B.; Dinér P. Mild and Rapid Aniline/HBF4•DEE-Catalysed Formation of Sulfinyl Imines. ChemistrySelect 2019, 4, 7431–7436. 10.1002/slct.201901218.

[ref55] Isor A.; Hommelsheim R.; Cone G. W.; Frings M.; Petroff Ii J. T.; Bolm C.; McCulla R. D. Photochemistry of N-Phenyl Dibenzothiophene Sulfoximine †. Photochem. Photobiol. 2021, 10.1111/php.13456. 34022069

[ref56] Stadtman E. R.; Van Remmen H.; Richardson A.; Wehr N. B.; Levine R. L. Methionine oxidation and aging. Biochim. Biophys. Acta 2005, 1703, 135–140. 10.1016/j.bbapap.2004.08.010. 15680221

[ref57] Tarrago L.; Péterfi Z.; Lee B. C.; Michel T.; Gladyshev V. N. Monitoring methionine sulfoxide with stereospecific mechanism-based fluorescent sensors. Nat. Chem. Biol. 2015, 11, 332–338. 10.1038/nchembio.1787. 25799144 PMC4402147

[ref58] Liu G.; Cogan D. A.; Ellman J. A. Catalytic Asymmetric Synthesis of tert-Butanesulfinamide. Application to the Asymmetric Synthesis of Amines. J. Am. Chem. Soc. 1997, 119, 9913–9914. 10.1021/ja972012z.

[ref59] Robak M. T.; Herbage M. A.; Ellman J. A. Synthesis and Applications of tert-Butanesulfinamide. Chem. Rev. 2010, 110, 3600–3740. 10.1021/cr900382t. 20420386

[ref60] Patureau F. W.; Worch C.; Siegler M. A.; Spek A. L.; Bolm C.; Reek J. N. H. SIAPhos: Phosphorylated Sulfonimidamides and their Use in Iridium-Catalyzed Asymmetric Hydrogenations of Sterically Hindered Cyclic Enamides. Adv. Synth. Catal. 2012, 354, 59–64. 10.1002/adsc.201100692.

[ref61] Steurer M.; Bolm C. Synthesis of amino-functionalized sulfonimidamides and their application in the enantioselective Henry reaction. J. Org. Chem. 2010, 75, 3301–3310. 10.1021/jo100326x. 20345140

[ref62] Bolm C.; Worch C. Use of prolyl sulfonimidamides in solvent-free organocatalytic asymmetric aldol reactions. Synlett 2009, 2009, 2425–2428. 10.1055/s-0029-1217727.

[ref63] Di Chenna P. H.; Robert-Peillard F.; Dauban P.; Dodd R. H. Sulfonimidamides: efficient chiral iminoiodane precursors for diastereoselective copper-catalyzed aziridination of olefins. Org. Lett. 2004, 6, 4503–4505. 10.1021/ol048167a. 15548061

[ref64] Leca D.; Toussaint A.; Mareau C.; Fensterbank L.; Lacôte E.; Malacria M. Efficient copper-mediated reactions of nitrenes derived from sulfonimidamides. Org. Lett. 2004, 6, 3573–3575. 10.1021/ol0485520. 15387551

[ref65] Fruit C.; Robert-Peillard F.; Bernardinelli G.; Müller P.; Dodd R. H.; Dauban P. Diastereoselective rhodium-catalyzed nitrene transfer starting from chiral sulfonimidamide-derived iminoiodanes. Tetrahedron: Asymmetry 2005, 16, 3484–3487. 10.1016/j.tetasy.2005.07.005.

[ref66] Liang C.; Robert-Peillard F.; Fruit C.; Müller P.; Dodd R. H.; Dauban P. Efficient Diastereoselective Intermolecular Rhodium-Catalyzed C–H Amination. Angew. Chem., Int. Ed. 2006, 45, 4641–4644. 10.1002/anie.200601248. 16789029

[ref67] Collet F.; Dodd R. H.; Dauban P. Stereoselective rhodium-catalyzed imination of sulfides. Org. Lett. 2008, 10, 5473–5476. 10.1021/ol802295b. 18986154

[ref68] Liang C.; Collet F.; Robert-Peillard F.; Müller P.; Dodd R. H.; Dauban P. Toward a synthetically useful stereoselective C– H amination of hydrocarbons. J. Am. Chem. Soc. 2008, 130, 343–350. 10.1021/ja076519d. 18072775

[ref69] Robert-Peillard F.; Di Chenna P. H.; Liang C.; Lescot C.; Collet F.; Dodd R. H.; Dauban P. Catalytic stereoselective alkene aziridination with sulfonimidamides. Tetrahedron: Asymmetry 2010, 21, 1447–1457. 10.1016/j.tetasy.2010.03.032.

[ref70] Lescot C.; Darses B.; Collet F.; Retailleau P.; Dauban P. Intermolecular C–H amination of complex molecules: Insights into the factors governing the selectivity. J. Org. Chem. 2012, 77, 7232–7240. 10.1021/jo301563j. 22892031

[ref71] Beltrán Á.; Lescot C.; Díaz-Requejo M. M.; Pérez P. J.; Dauban P. Catalytic C–H amination of alkanes with sulfonimidamides: silver (I)-scorpionates vs. dirhodium (II) carboxylates. Tetrahedron 2013, 69, 4488–4492. 10.1016/j.tet.2013.02.005.

[ref72] Morales S.; Guijarro F. G.; García Ruano J. L.; Cid M. B. A general aminocatalytic method for the synthesis of aldimines. J. Am. Chem. Soc. 2014, 136, 1082–1089. 10.1021/ja4111418. 24359453

[ref73] Cogan D. A.; Liu G.; Ellman J. Asymmetric synthesis of chiral amines by highly diastereoselective 1,2-additions of organometallic reagents to N-tert-butanesulfinyl imines. Tetrahedron 1999, 55, 8883–8904. 10.1016/s0040-4020(99)00451-2.

[ref74] Pflum D. A.; Krishnamurthy D.; Han Z.; Wald S. A.; Senanayake C. H. Asymmetric synthesis of cetirizine dihydrochloride. Tetrahedron Lett. 2002, 43, 923–926. 10.1016/s0040-4039(01)02294-8.

[ref75] Plobeck N.; Powell D. Asymmetric synthesis of diarylmethylamines by diastereoselective addition of organometallic reagents to chiral N-tert-butanesulfinimines: switchover of diastereofacial selectivity. Tetrahedron: Asymmetry 2002, 13, 303–310. 10.1016/s0957-4166(02)00099-x.

[ref76] Cogan D. A.; Ellman J. A. Asymmetric Synthesis of α,α-Dibranched Amines by the Trimethylaluminum-Mediated 1,2-Addition of Organolithiums to tert-Butanesulfinyl Ketimines. J. Am. Chem. Soc. 1999, 121, 268–269. 10.1021/ja983217q.

[ref77] Shatskiy A.; Axelsson A.; Stepanova E. V.; Liu J.-Q.; Temerdashev A. Z.; Kore B. P.; Blomkvist B.; Gardner J. M.; Dinér P.; Kärkäs M. D. Stereoselective synthesis of unnatural α-amino acid derivatives through photoredox catalysis. Chem. Sci. 2021, 12, 5430–5437. 10.1039/d1sc00658d. 34168785 PMC8179686

[ref78] Sun P.; Weinreb S. M.; Shang M. tert-Butylsulfonyl (Bus), a New Protecting Group for Amines. J. Org. Chem. 1997, 62, 8604–8608. 10.1021/jo971455i. 11672017

[ref79] Xie S.; Lopez S. A.; Ramström O.; Yan M.; Houk K. N. 1,3-Dipolar Cycloaddition Reactivities of Perfluorinated Aryl Azides with Enamines and Strained Dipolarophiles. J. Am. Chem. Soc. 2015, 137, 2958–2966. 10.1021/ja511457g. 25553488 PMC4351169

