# Supplementary material for: Accessing Perfluoroaryl Sulfonimidamides and Sulfoximines via Photogenerated Perfluoroaryl Nitrenes: Synthesis and Application as a Chiral Auxiliary
Source: J Org Chem. 2021 Nov 12;86(23):17119–28. doi: 10.1021/acs.joc.1c02241 (PMC8650101; doi:10.1021/acs.joc.1c02241)

# Supporting information

## Accessing Perfluoroaryl Sulfonimidamides and Sulfoximines via Photogenerated Perfluoroaryl Nitrenes: Synthesis and Application as a Chiral Auxiliary

Giampiero Proietti,‡ Julius Kuzmin,‡ Azamat Z. Temerdashev† and Peter Dinér*‡

‡ Division of Organic Chemistry, Department of Chemistry, KTH-Royal Institute of Technology, Teknikringen 30, 10044 Stockholm (Sweden)

E-mail: diner@kth.se

† Department of Analytical Chemistry, Kuban State University, Stavropolskaya St. 149, 350040 Krasnodar (Russia)

## Table of contents

NMR Spectra synthesized starting material S2-S7

NMR Spectra isolated products S8-S39

NMR spectra and HPLC chromatograms for the Grignard additions screening S39-S43

HRMS spectra S44-S64

## NMR Spectra synthesized starting material

### Compound **1a**

$^{1}H$ NMR ($CDCl_3$, 400 MHz) **1a**

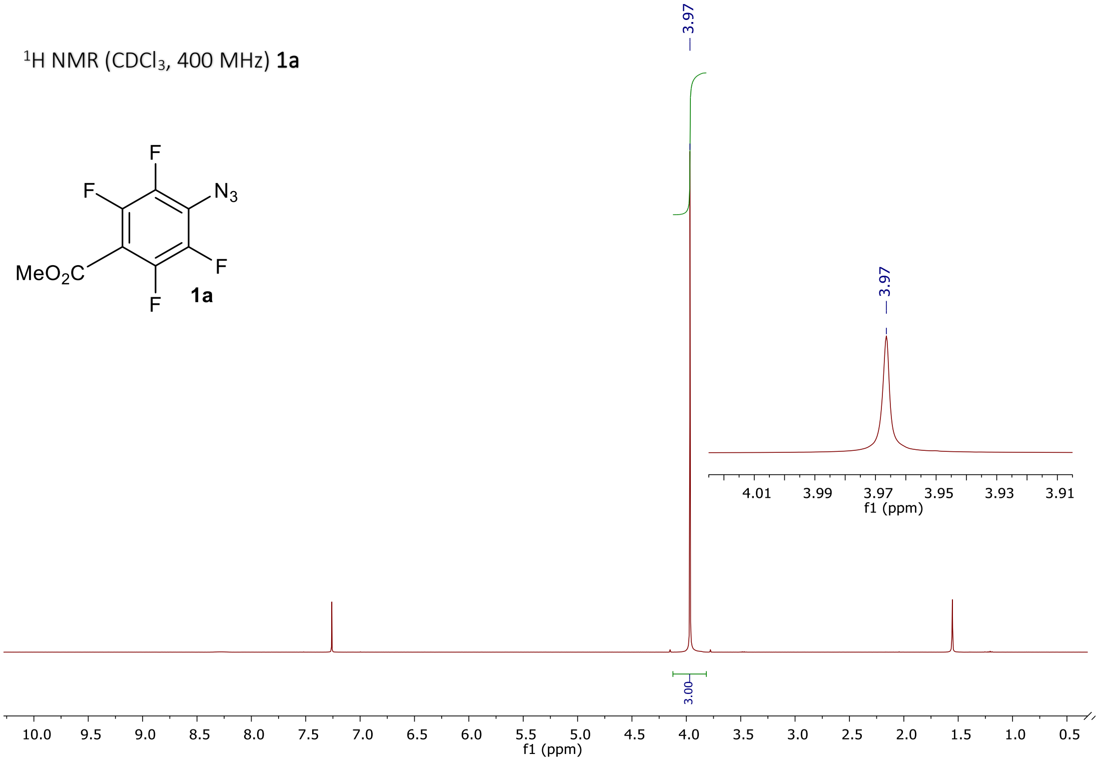


$^{13}C\{^{1}H\}$ NMR ($CDCl_3$, 100 MHz) **1a**

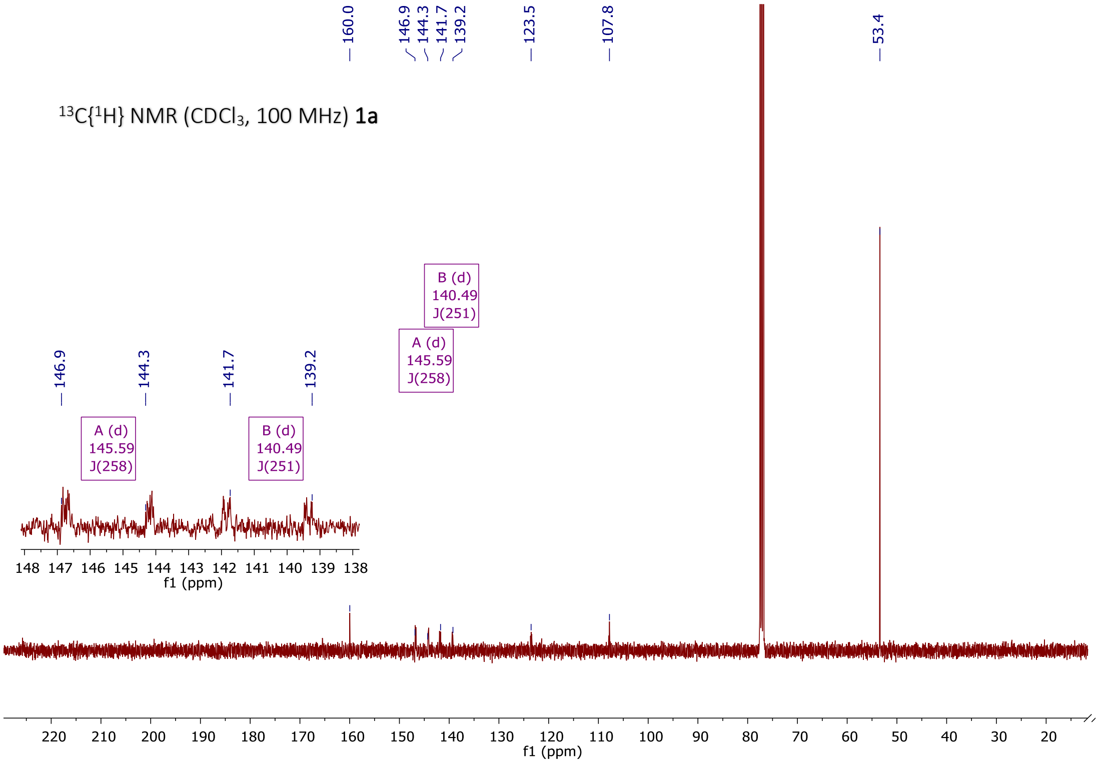

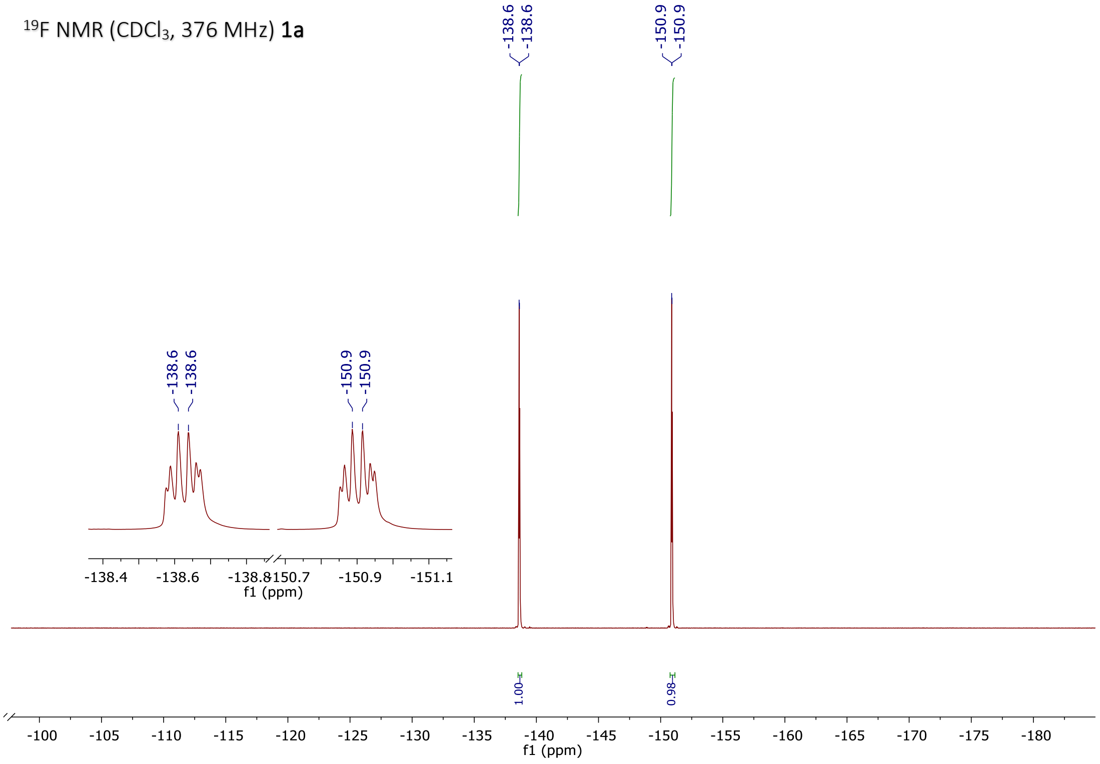

¹⁹F NMR (CDCl₃, 376 MHz) 1a
-138.6
-138.6
-150.9
-150.9
1.00
0.98
f1 (ppm)


## Compound **1b**

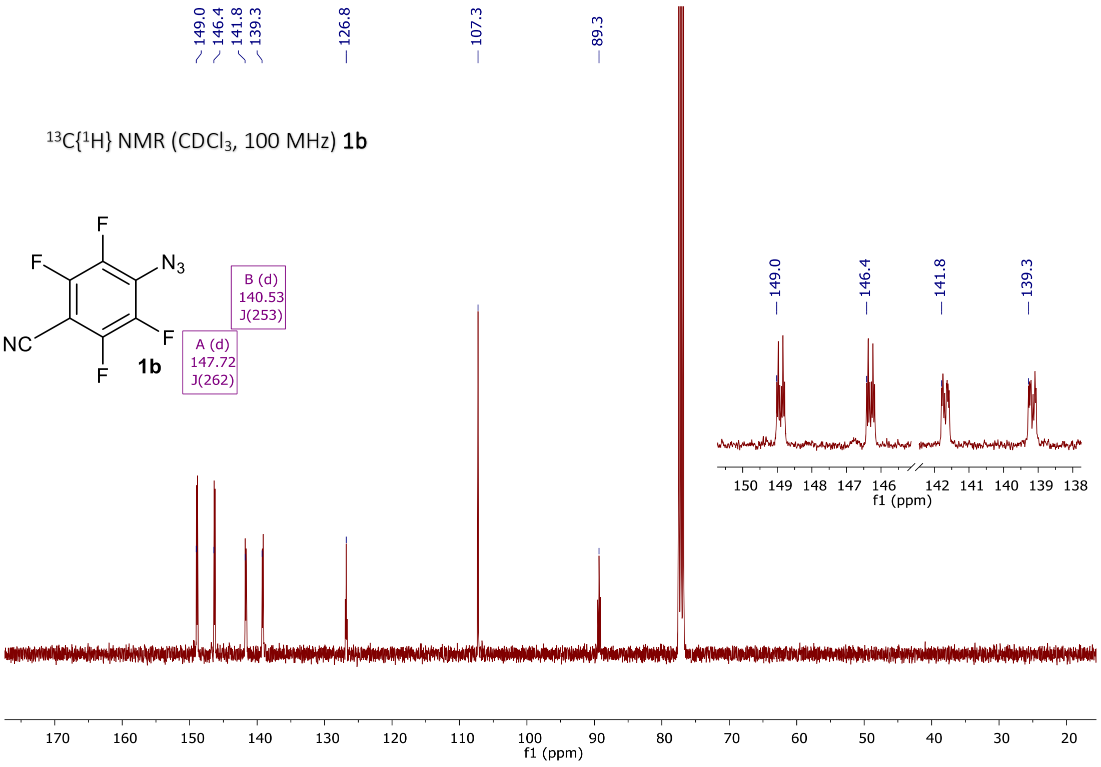

149.0
146.4
141.8
139.3
126.8
107.3
89.3
¹³C{¹H} NMR (CDCl₃, 100 MHz) 1b
F
F
N₃
NC
F
F
1b
B (d)
140.53
J(253)
A (d)
147.72
J(262)
f1 (ppm)

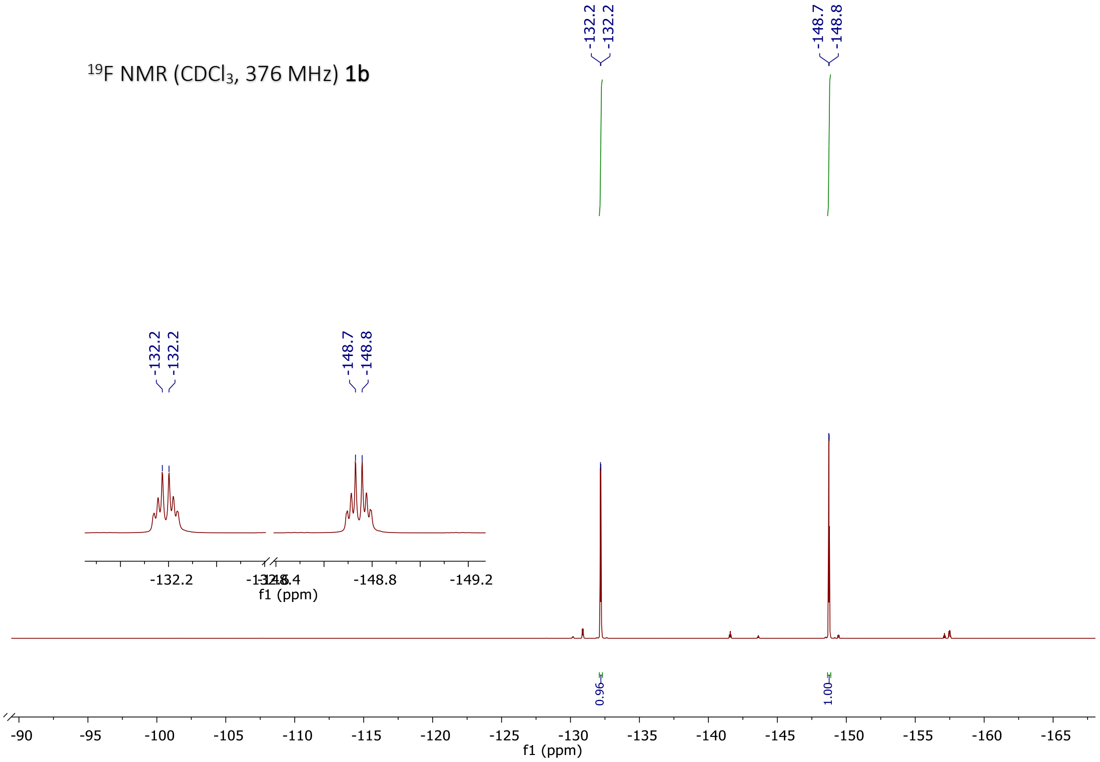

$^{19}$F NMR (CDCl$_3$, 376 MHz) **1b**
-132.2
-132.2
-148.7
-148.8
-132.2
-148.8
-149.2
f1 (ppm)
0.96
1.00
-90 -95 -100 -105 -110 -115 -120 -125 -130 -135 -140 -145 -150 -155 -160 -165
f1 (ppm)


## Compound **1c**

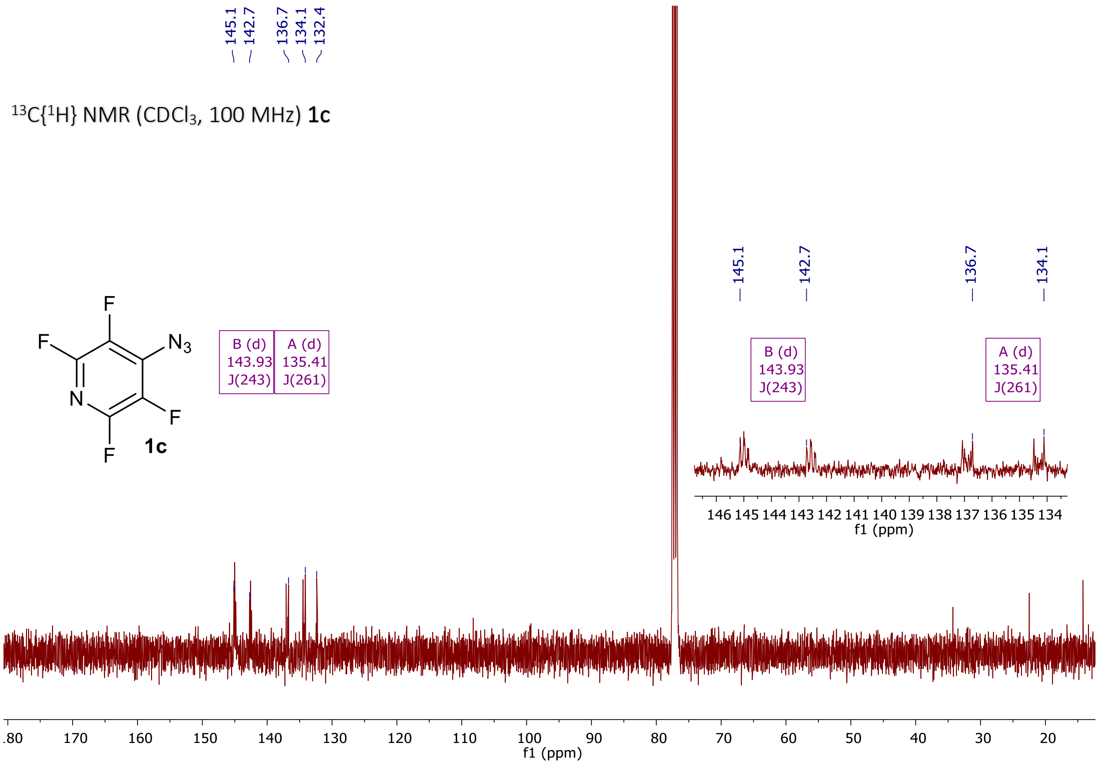

145.1
142.7
136.7
134.1
132.4
$^{13}$C{$^1$H} NMR (CDCl$_3$, 100 MHz) **1c**
F
F
N$_3$
N
F
F
1c
B (d)
143.93
J(243)
A (d)
135.41
J(261)
145.1
142.7
136.7
134.1
146 145 144 143 142 141 140 139 138 137 136 135 134
f1 (ppm)
180 170 160 150 140 130 120 110 100 90 80 70 60 50 40 30 20
f1 (ppm)

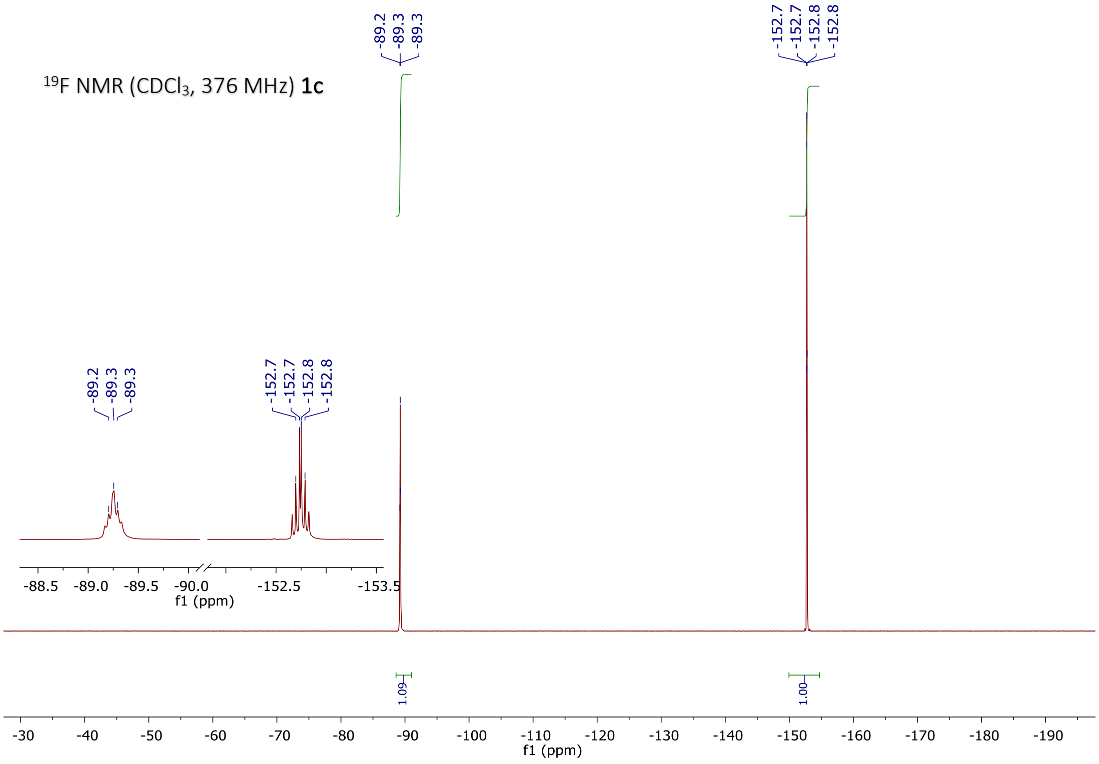

-89.2
-89.3
-89.3
-152.7
-152.7
-152.8
-152.8
$^{19}$F NMR ($CDCl_3$, 376 MHz) **1c**
-88.5 -89.0 -89.5 -90.0 -152.5 -153.5
f1 (ppm)
1.09
1.00
-30 -40 -50 -60 -70 -80 -90 -100 -110 -120 -130 -140 -150 -160 -170 -180 -190
f1 (ppm)


## Compound **1d**

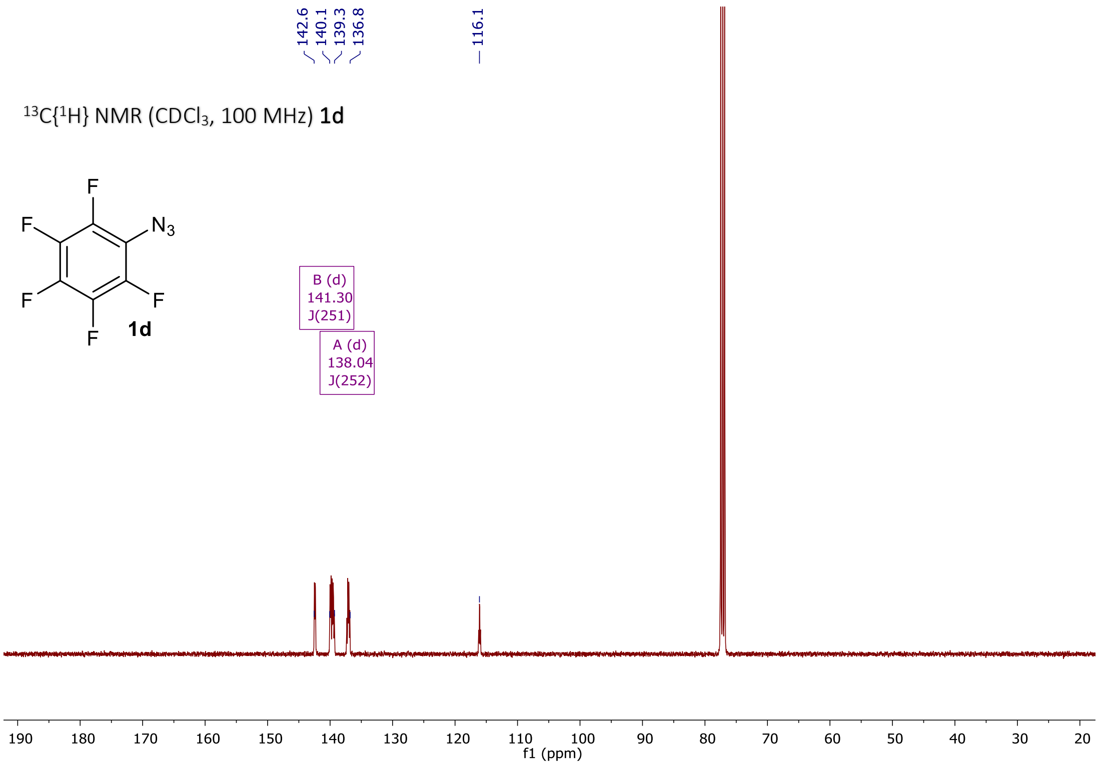

142.6
140.1
139.3
136.8
116.1
$^{13}$C{$^1$H} NMR ($CDCl_3$, 100 MHz) **1d**
F
F
N3
F
F
F
1d
B (d)
141.30
J(251)
A (d)
138.04
J(252)
190 180 170 160 150 140 130 120 110 100 90 80 70 60 50 40 30 20
f1 (ppm)

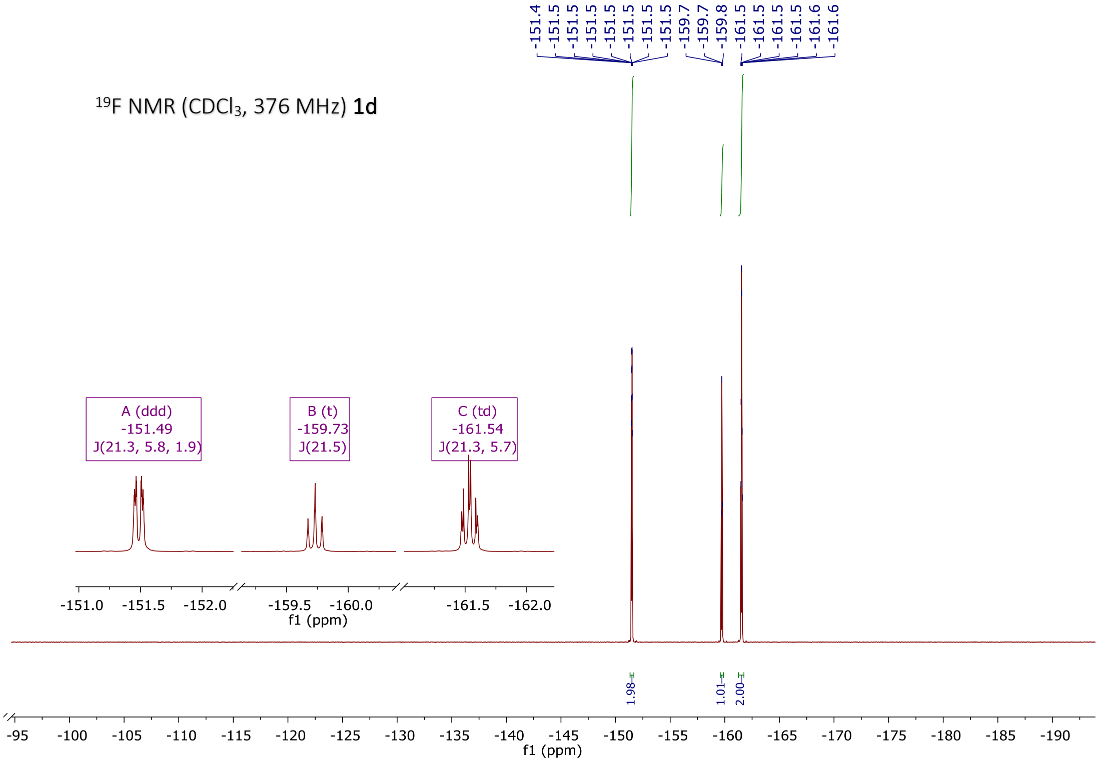

-151.4
-151.5
-151.5
-151.5
-151.5
-151.5
-151.5
-151.5
-159.7
-159.7
-159.8
-161.5
-161.5
-161.5
-161.5
-161.6
-161.6
$^{19}F$ NMR ($CDCl_3$, 376 MHz) **1d**
A (ddd) -151.49 J(21.3, 5.8, 1.9)
B (t) -159.73 J(21.5)
C (td) -161.54 J(21.3, 5.7)
1.98
1.01
2.00
f1 (ppm)


Compound **1e**

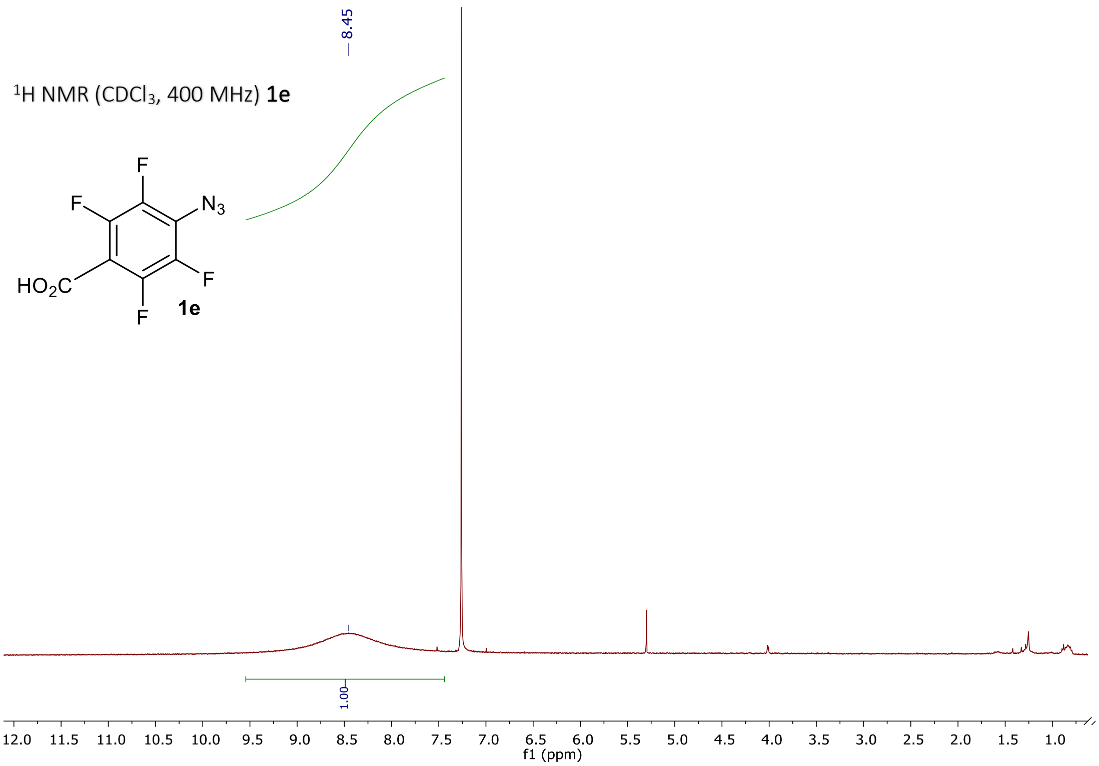

8.45
$^{1}H$ NMR ($CDCl_3$, 400 MHz) **1e**
F
F
$N_3$
$HO_2C$
F
F
1e
1.00
f1 (ppm)

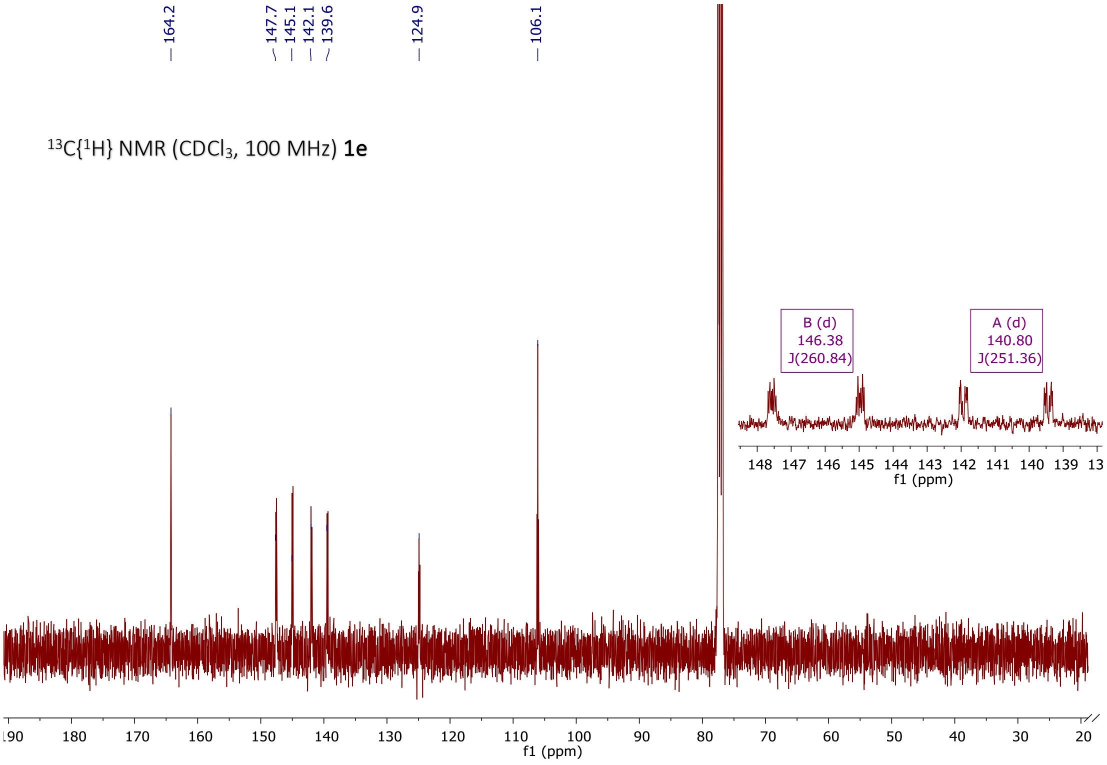


$^{13}C\{^{1}H\}$ NMR ($CDCl_3$, 100 MHz) **1e**

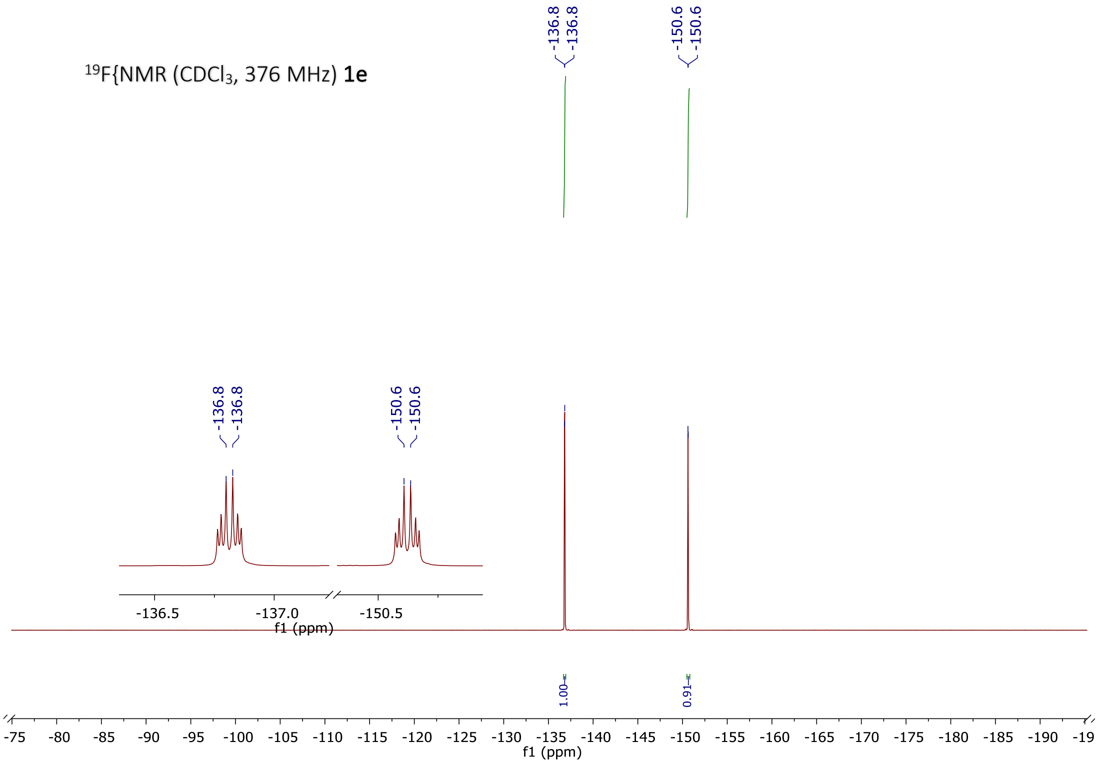


$^{19}F$\{NMR ($CDCl_3$, 376 MHz) **1e**

## 1. NMR Spectra isolated products

Compound (*S*)-**1**

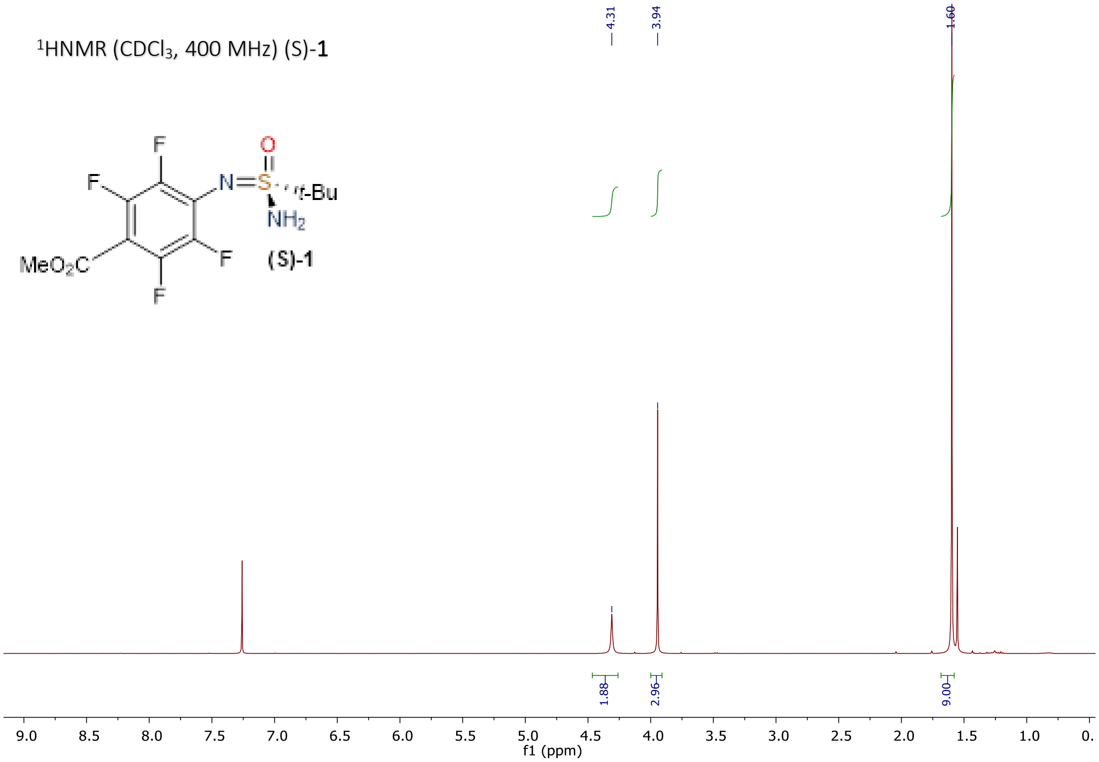


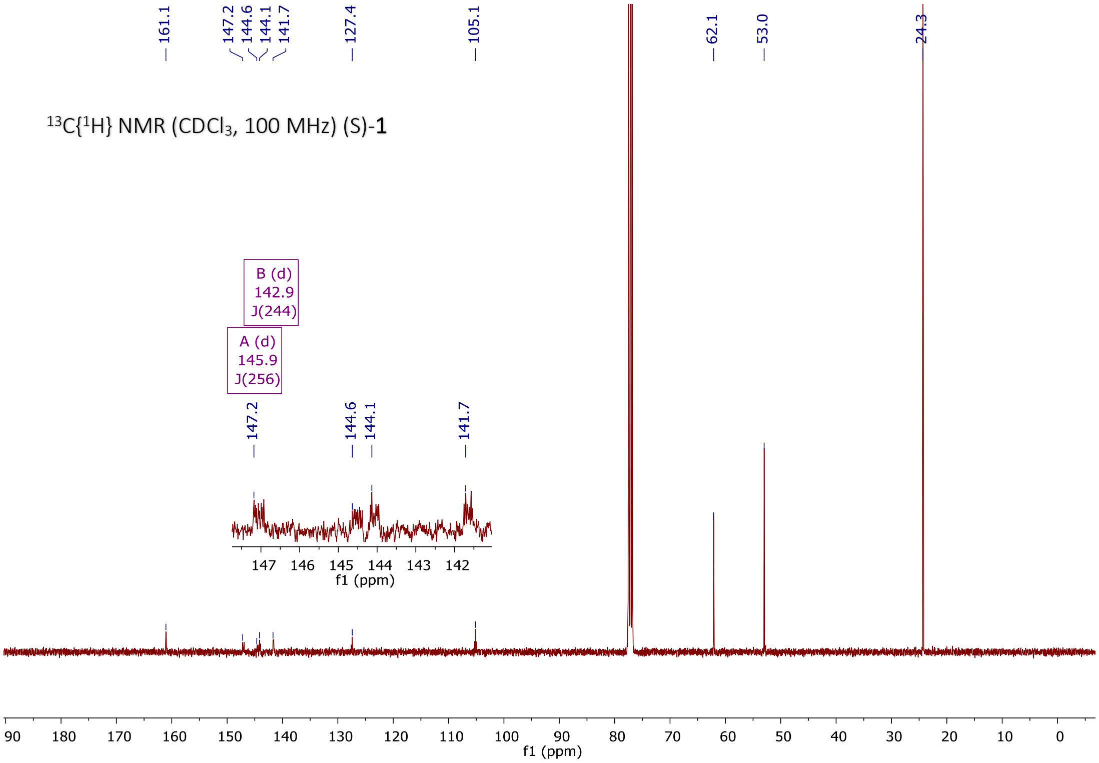

$^{19}$F NMR ($CDCl_3$, 376 MHz) (S)-**1**

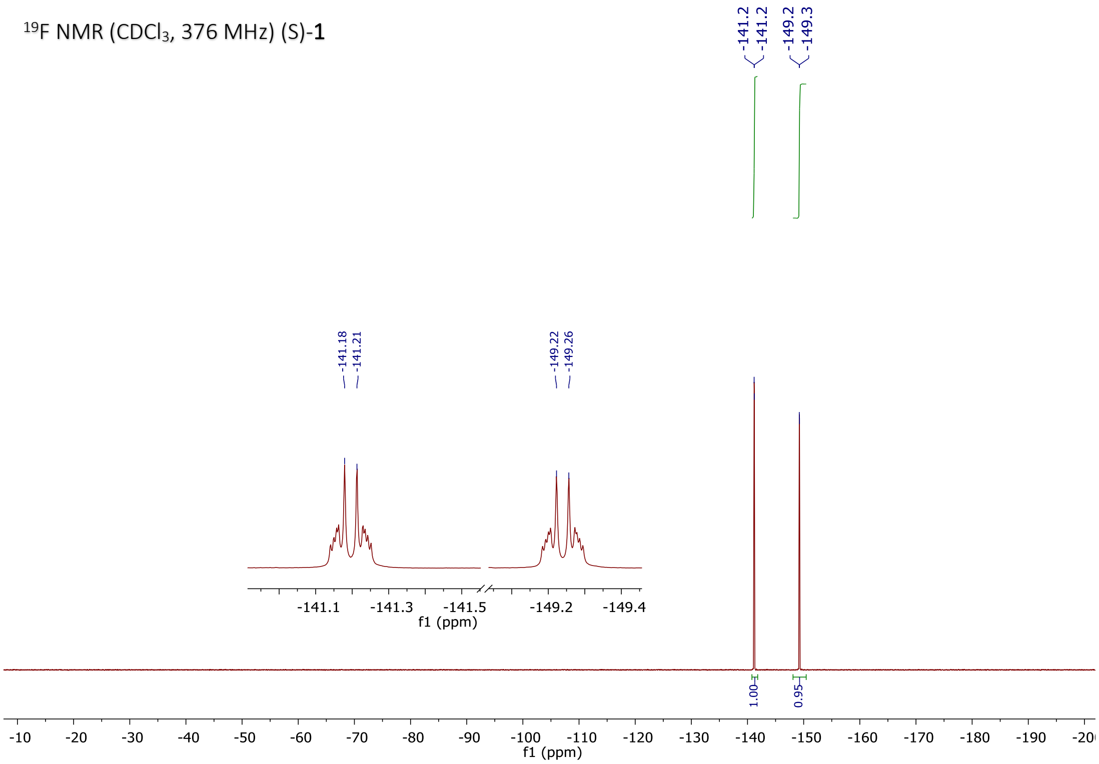


Compound (*R*)**-1**

$^{1}$HNMR ($CDCl_3$, 400 MHz) (R)-**1**

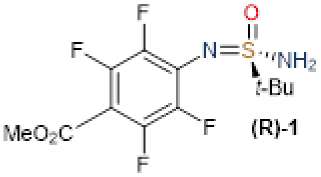


(R)-1

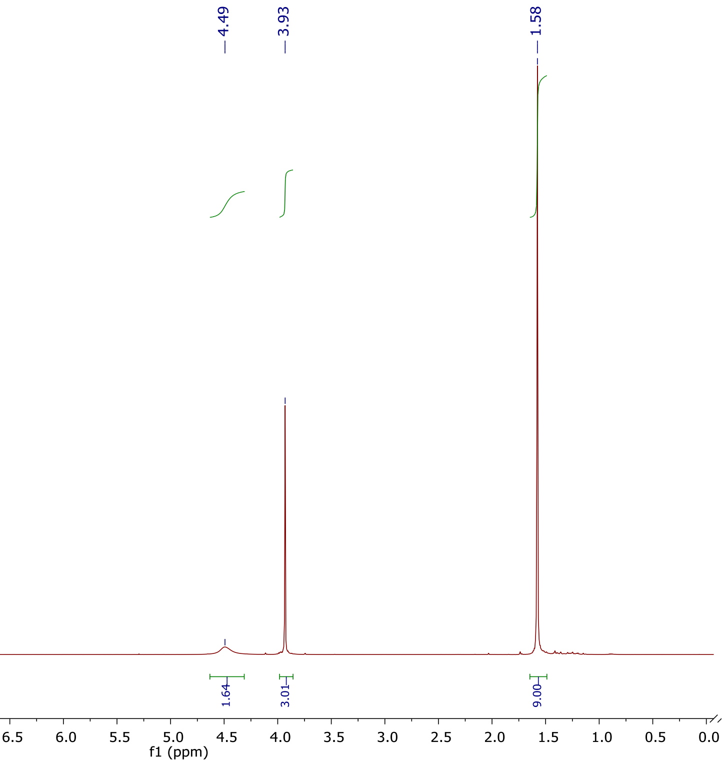

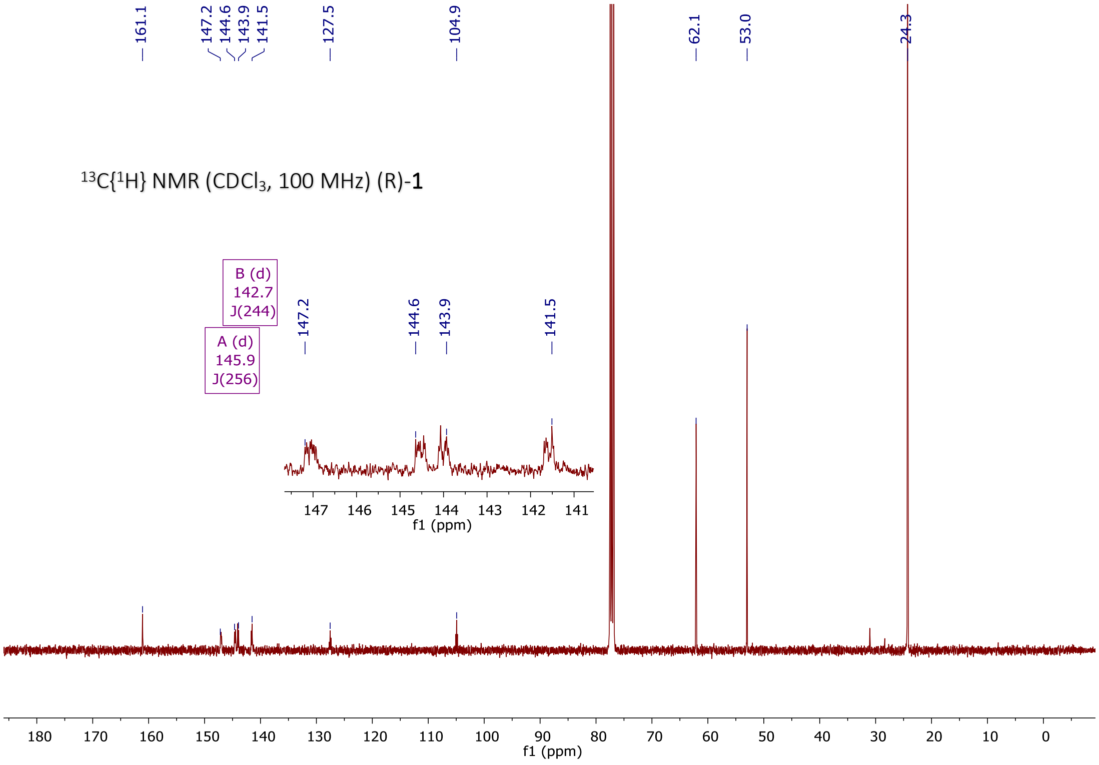
161.1
147.2
144.6
143.9
141.5
127.5
104.9
62.1
53.0
24.3
$^{13}C\{^{1}H\}$ NMR ($CDCl_3$, 100 MHz) (R)-**1**
B (d)
142.7
J(244)
A (d)
145.9
J(256)
147.2
144.6
143.9
141.5
147 146 145 144 143 142 141
f1 (ppm)
180 170 160 150 140 130 120 110 100 90 80 70 60 50 40 30 20 10 0
f1 (ppm)

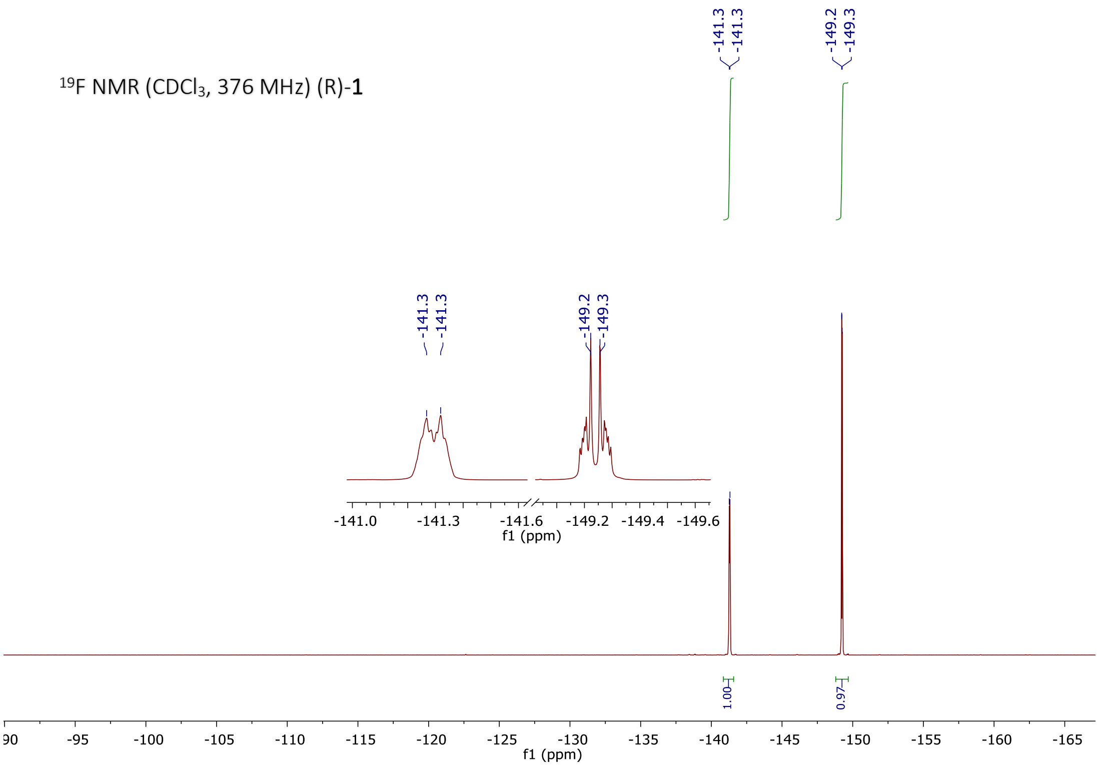
-141.3
-141.3
-149.2
-149.3
$^{19}F$ NMR ($CDCl_3$, 376 MHz) (R)-**1**
-141.3
-141.3
-149.2
-149.3
-141.0 -141.3 -141.6 -149.2 -149.4 -149.6
f1 (ppm)
1.00
0.97
-90 -95 -100 -105 -110 -115 -120 -125 -130 -135 -140 -145 -150 -155 -160 -165
f1 (ppm)

Compound (*S*)-**2**

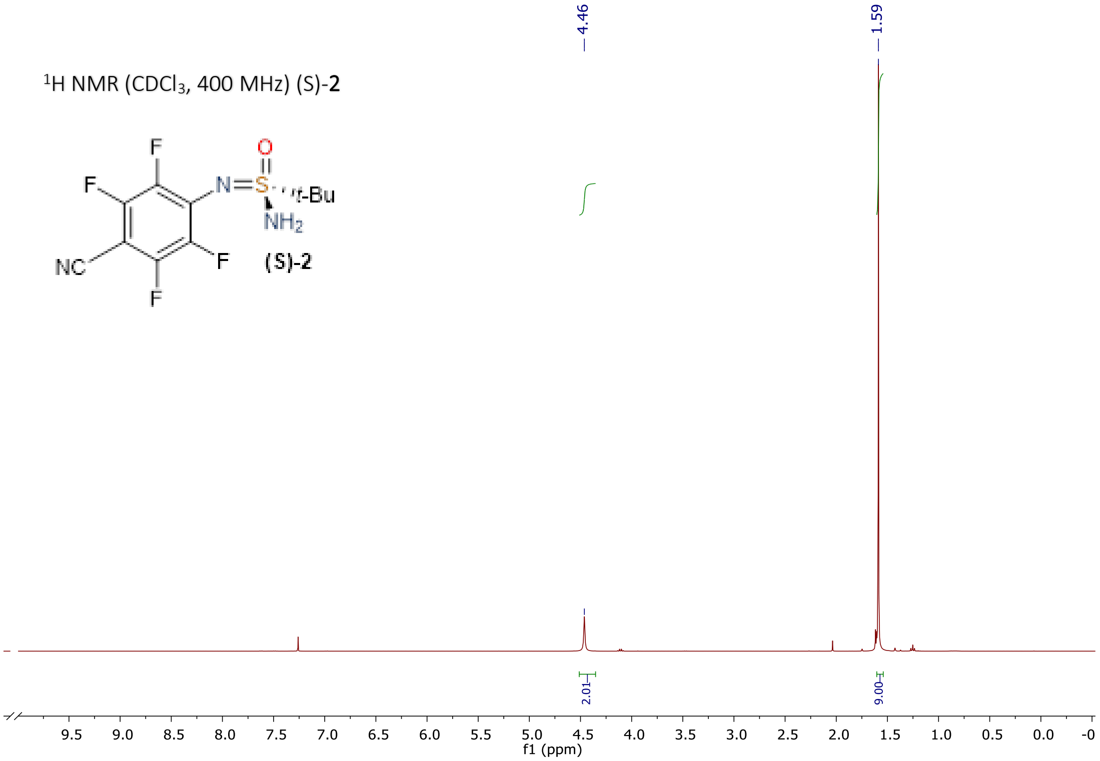

$^{1}H$ NMR ($CDCl_3$, 400 MHz) (S)-**2**
(S)-2
4.46
1.59
2.01
9.00
f1 (ppm)


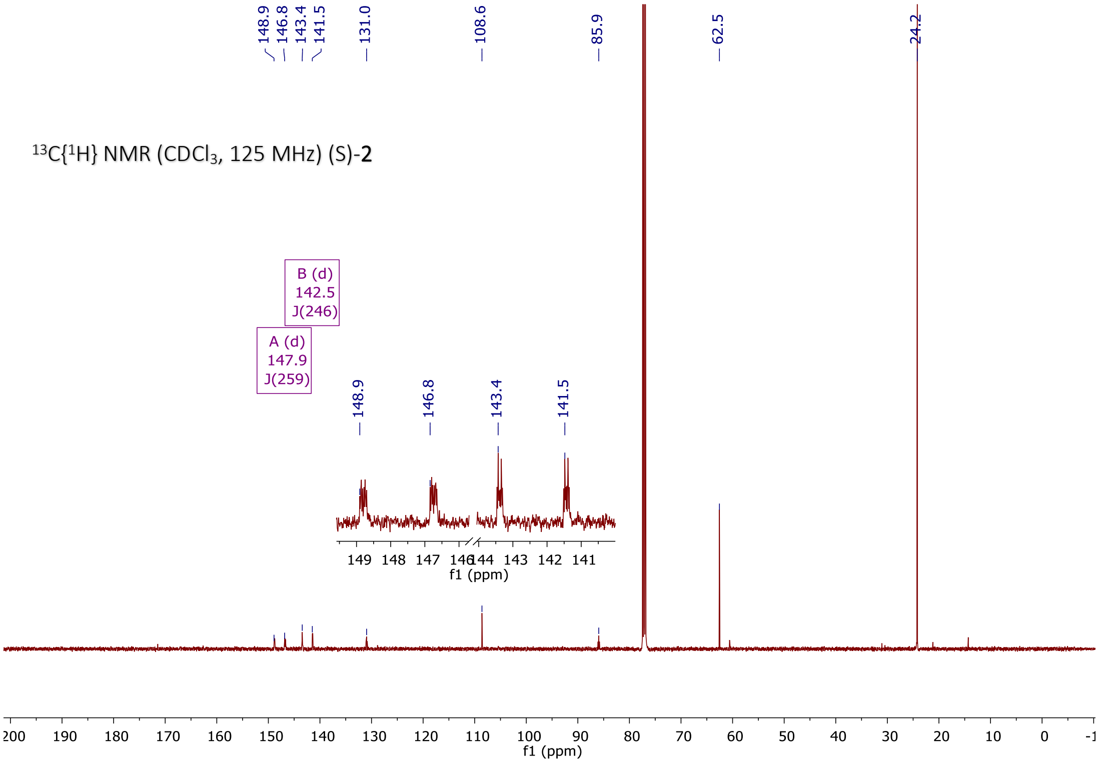

$^{13}C\{^{1}H\}$ NMR ($CDCl_3$, 125 MHz) (S)-**2**
148.9
146.8
143.4
141.5
131.0
108.6
85.9
62.5
24.2
B (d)
142.5
J(246)
A (d)
147.9
J(259)
f1 (ppm)

$^{19}$F NMR ($CDCl_3$, 376 MHz) (S)-**2**

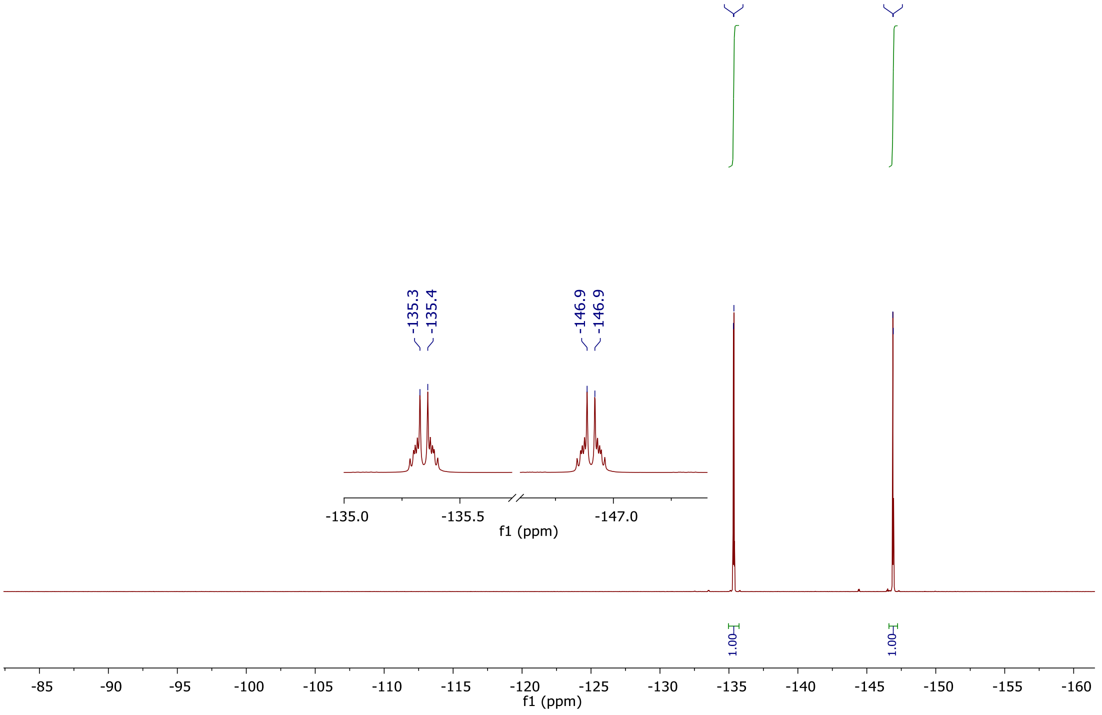


## Compound (*S*)-**3**

$^{1}$H NMR ($CDCl_3$, 400 MHz) (S)-**3**

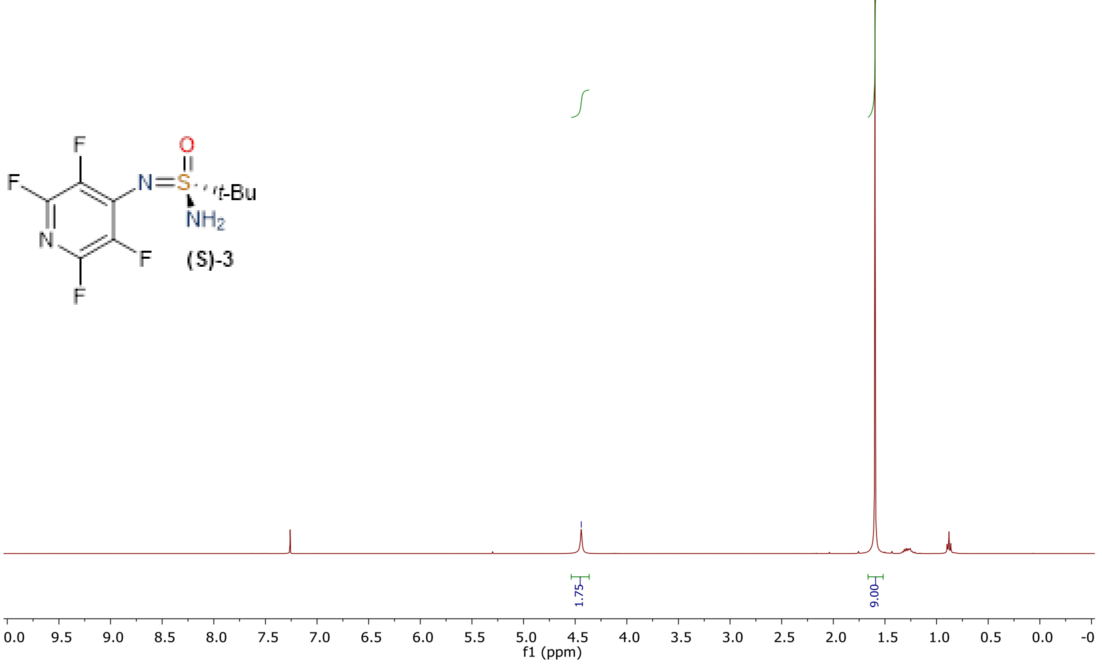

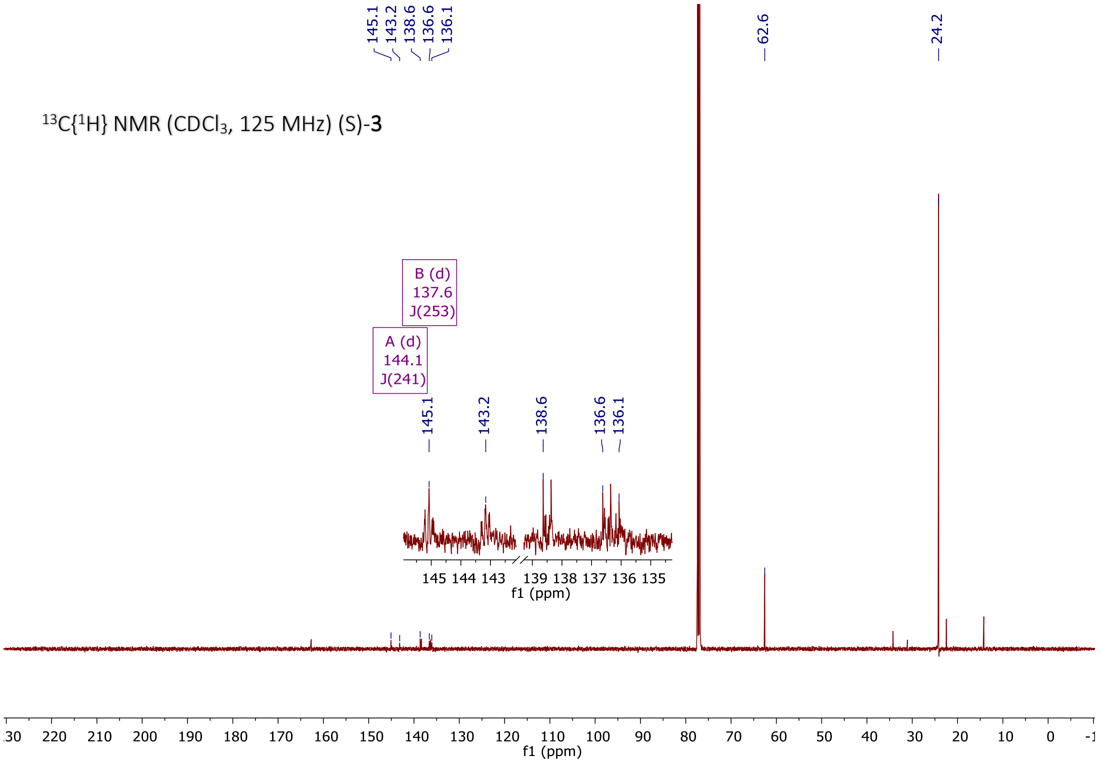


$^{13}C\{^{1}H\}$ NMR ($CDCl_3$, 125 MHz) (S)-**3**

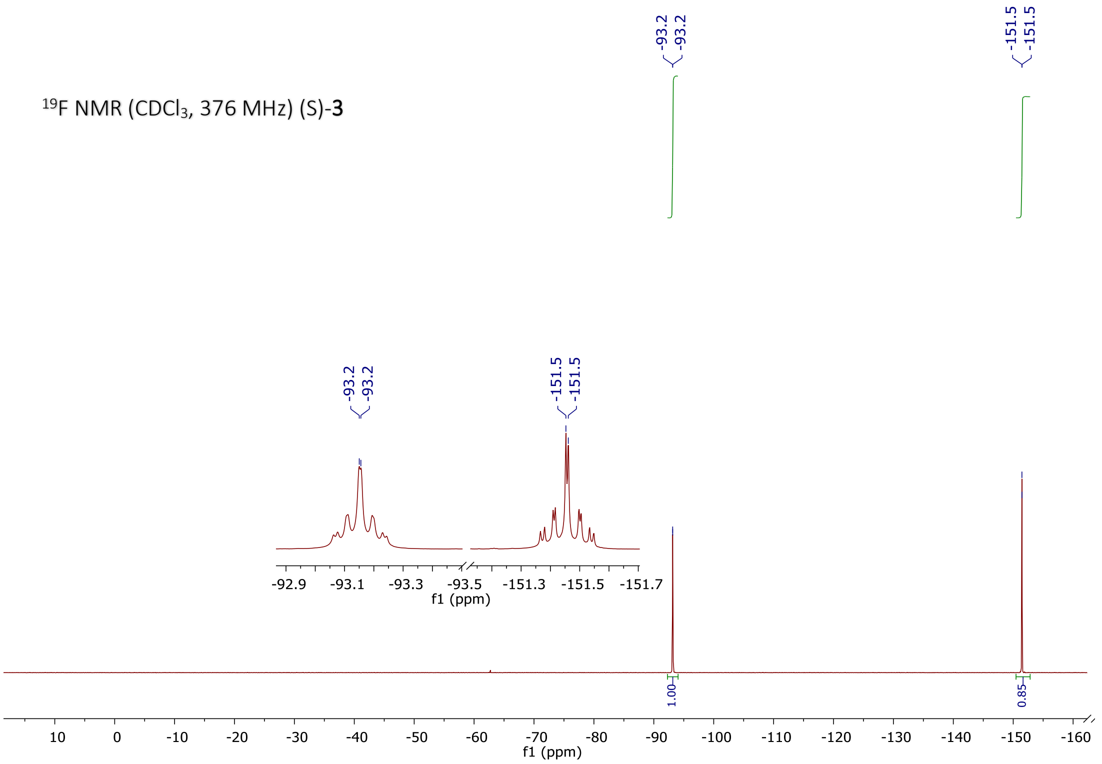


$^{19}F$ NMR ($CDCl_3$, 376 MHz) (S)-**3**

Compound *(R)-3*

$^{1}$H NMR ($CDCl_3$, 400 MHz) (R)-**3**

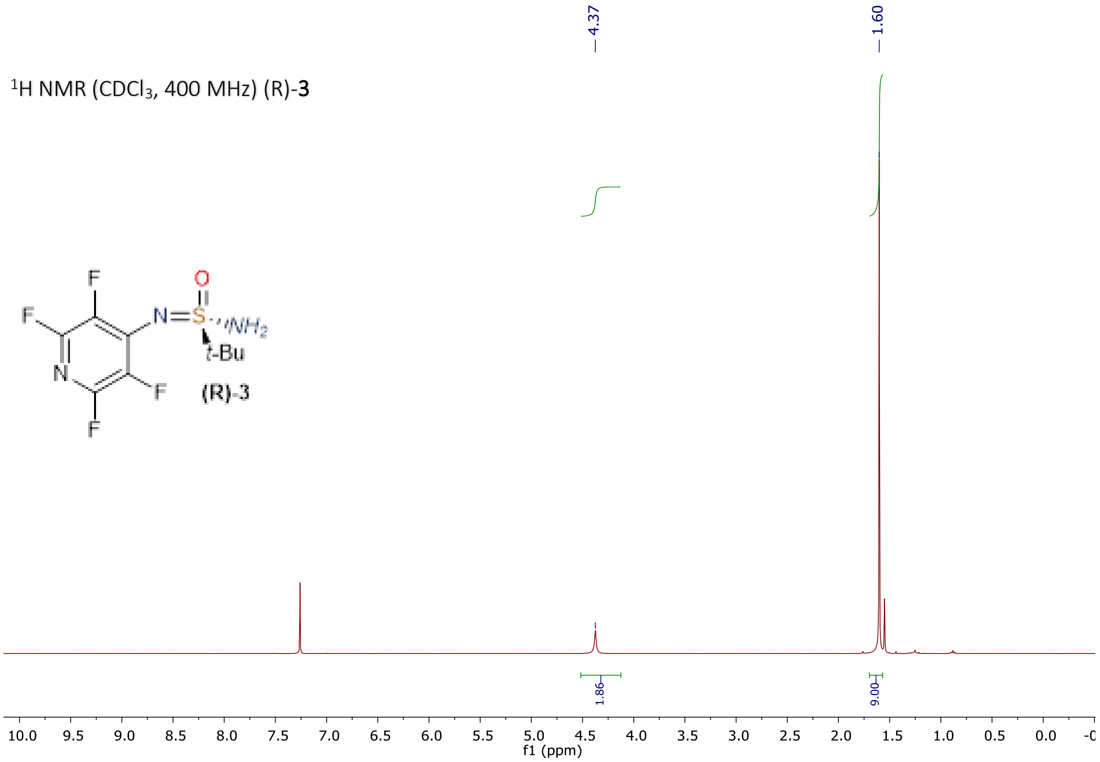


$^{13}$C{$^{1}$H} NMR ($CDCl_3$, 100 MHz) (R)-**3**

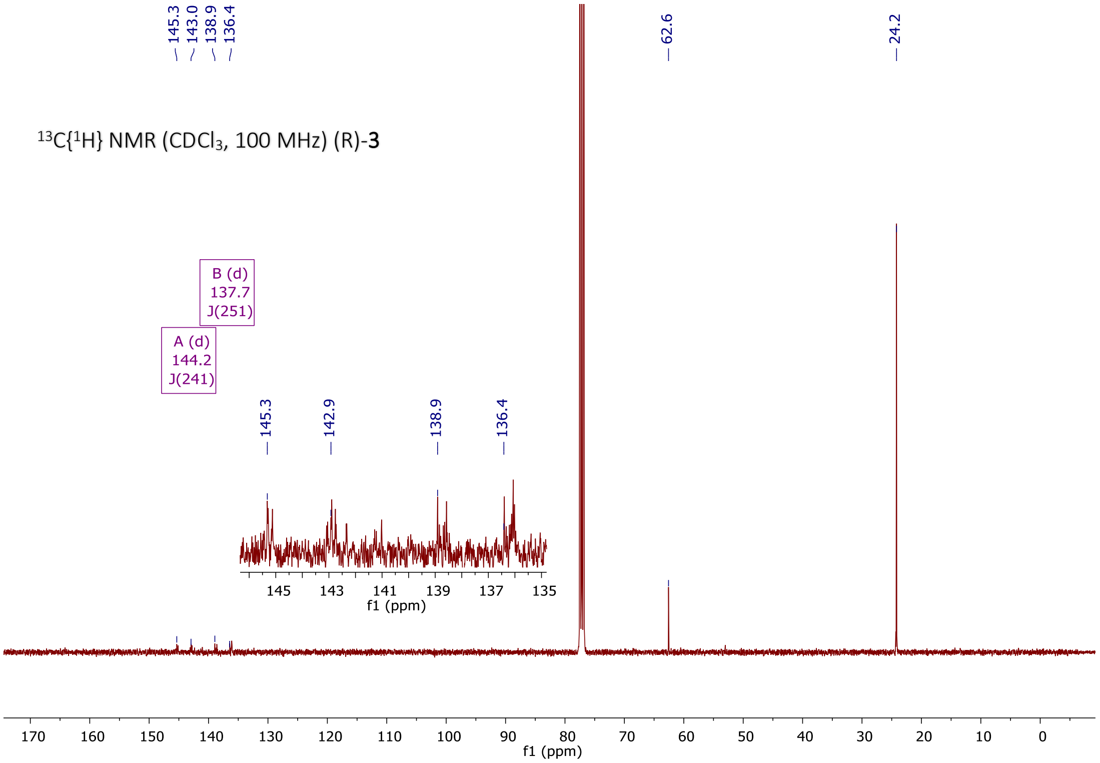

$^{19}F$ NMR ($CDCl_3$, 376 MHz) (R)-**3**

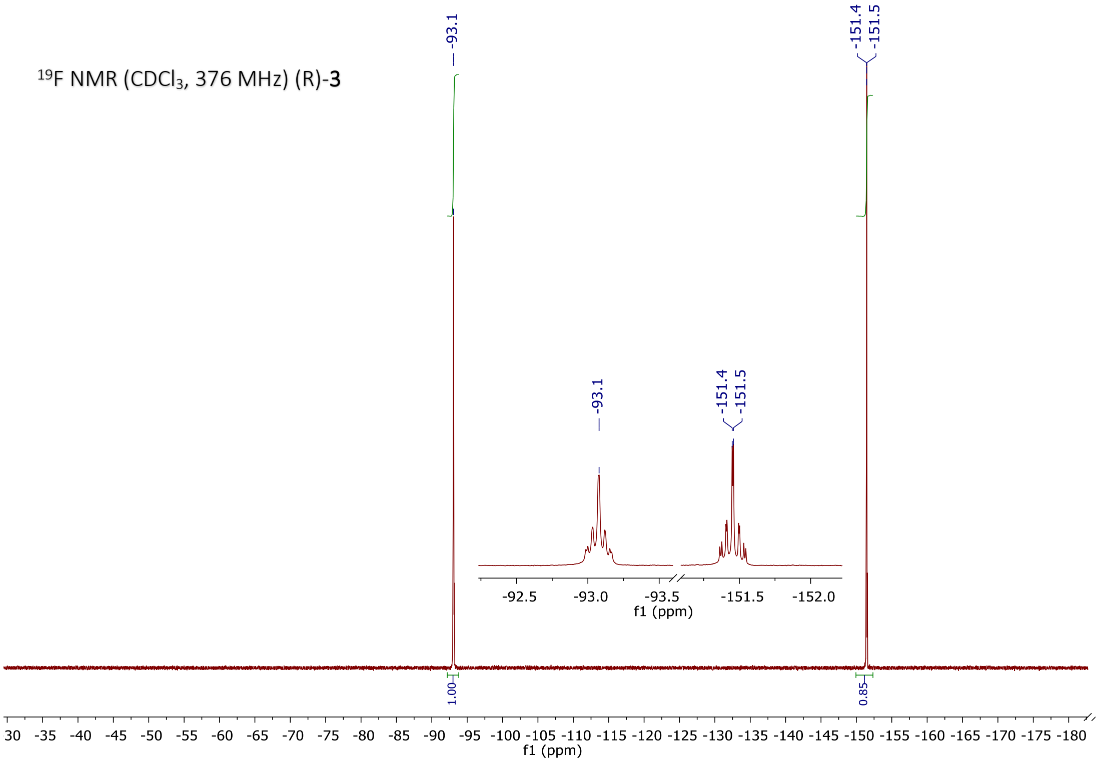


## Compound (*S*)-**4**

$^{1}H$ NMR ($CDCl_3$, 400 MHz) (S)-**4**

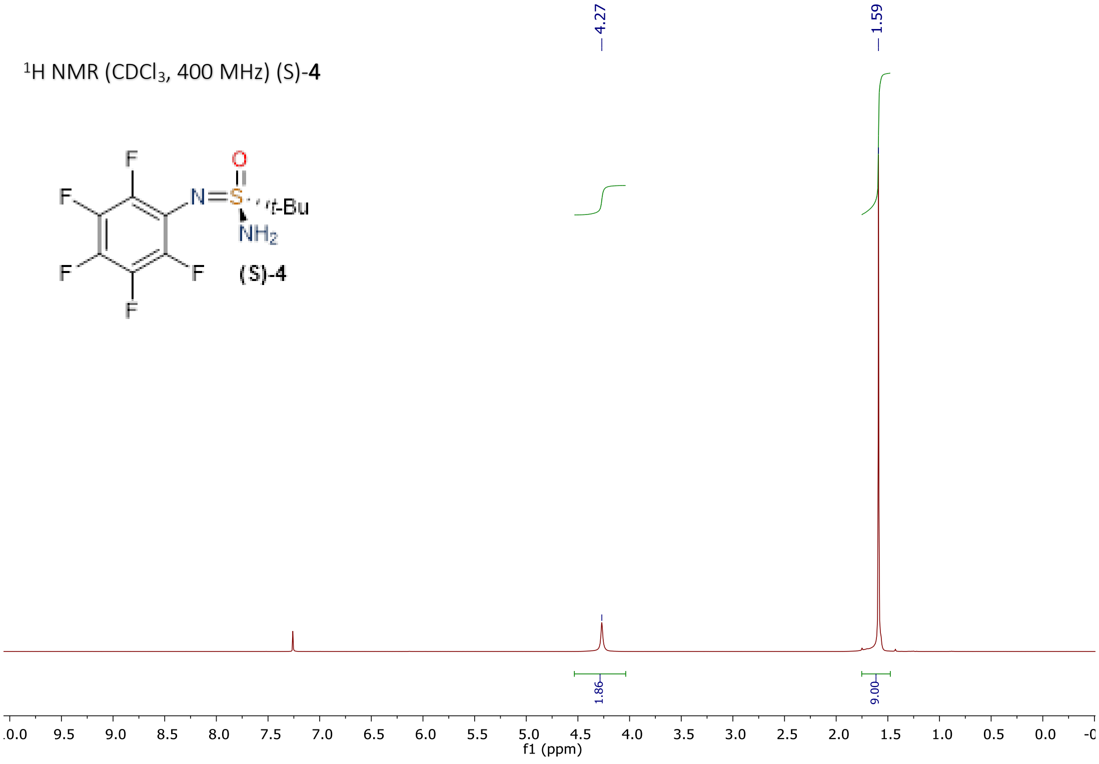

$^{13}C\{^1H\}$ NMR ($CDCl_3$, 125 MHz) (S)-**4**

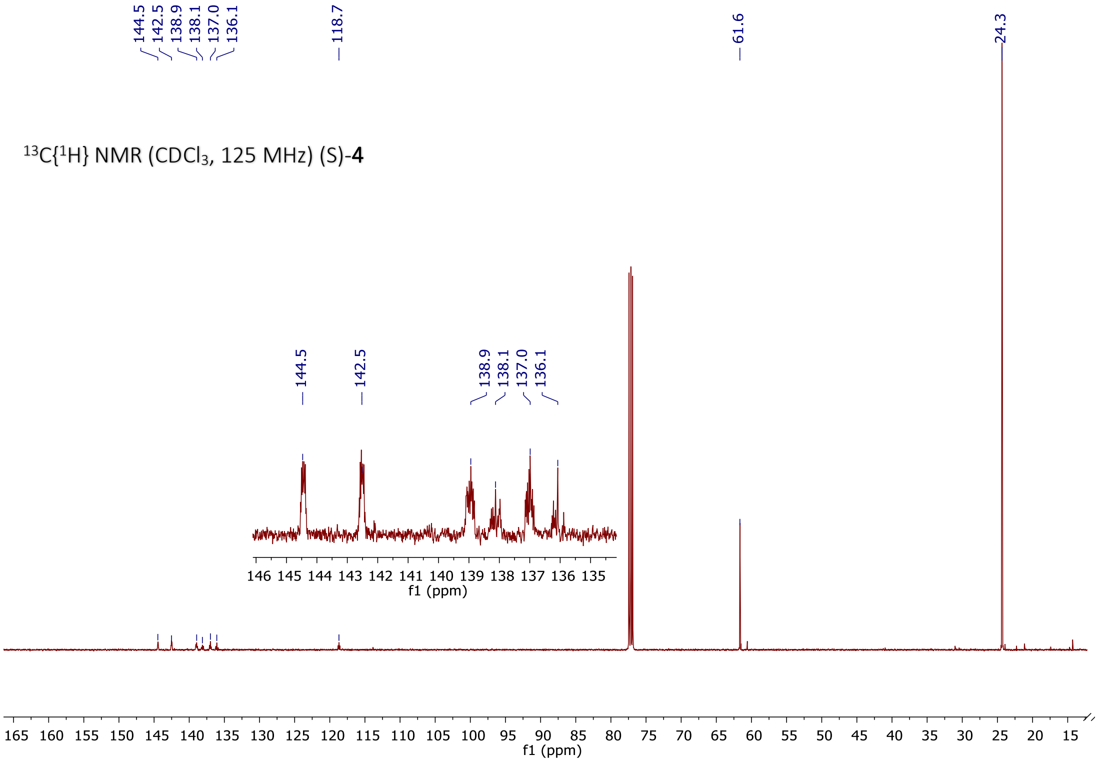


$^{19}F$ NMR ($CDCl_3$, 376 MHz) (S)-**4**

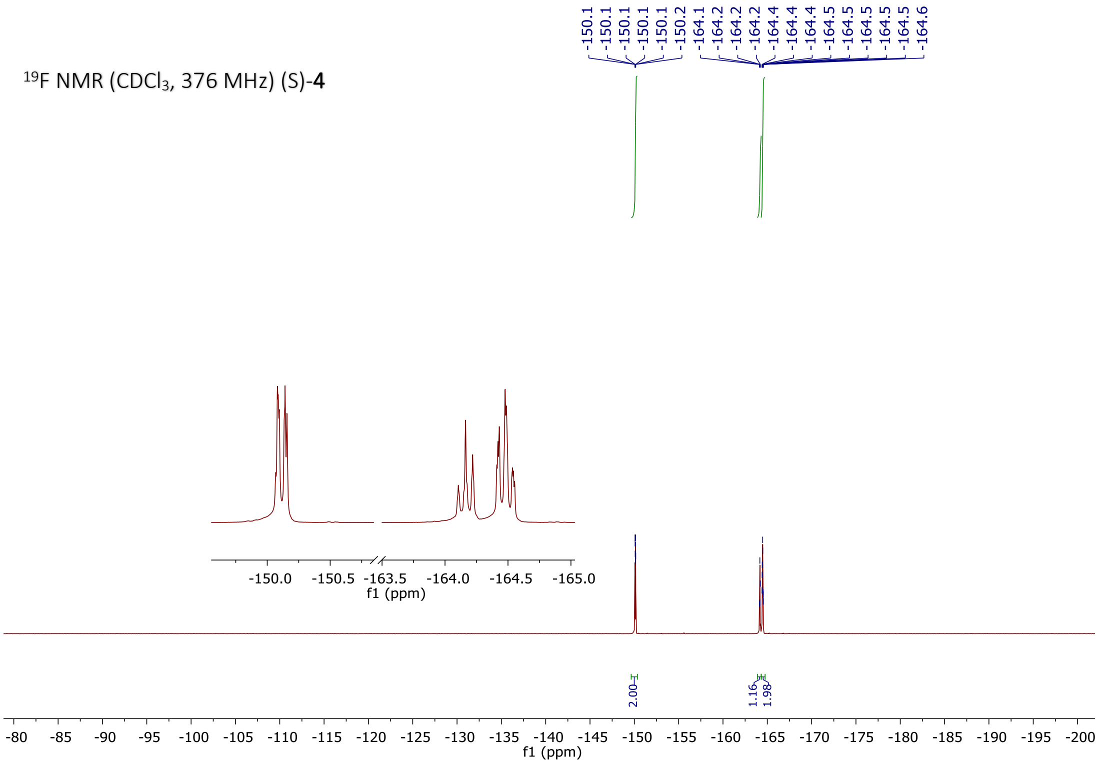

## Compound (*S*)-**5**

$^{1}$H NMR (DMSO-d$_6$, 400 MHz) (S)-**5**

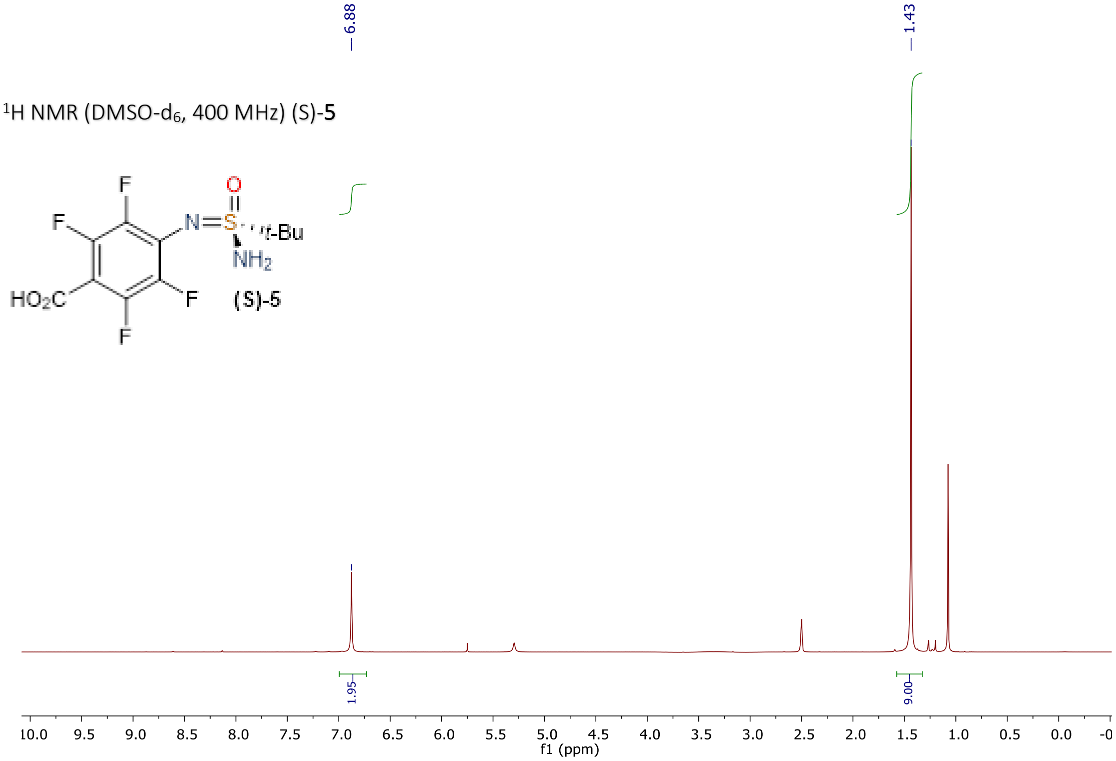


$^{13}$C{$^{1}$H} NMR (DMSO-d$_6$, 125 MHz) (S)-**5**

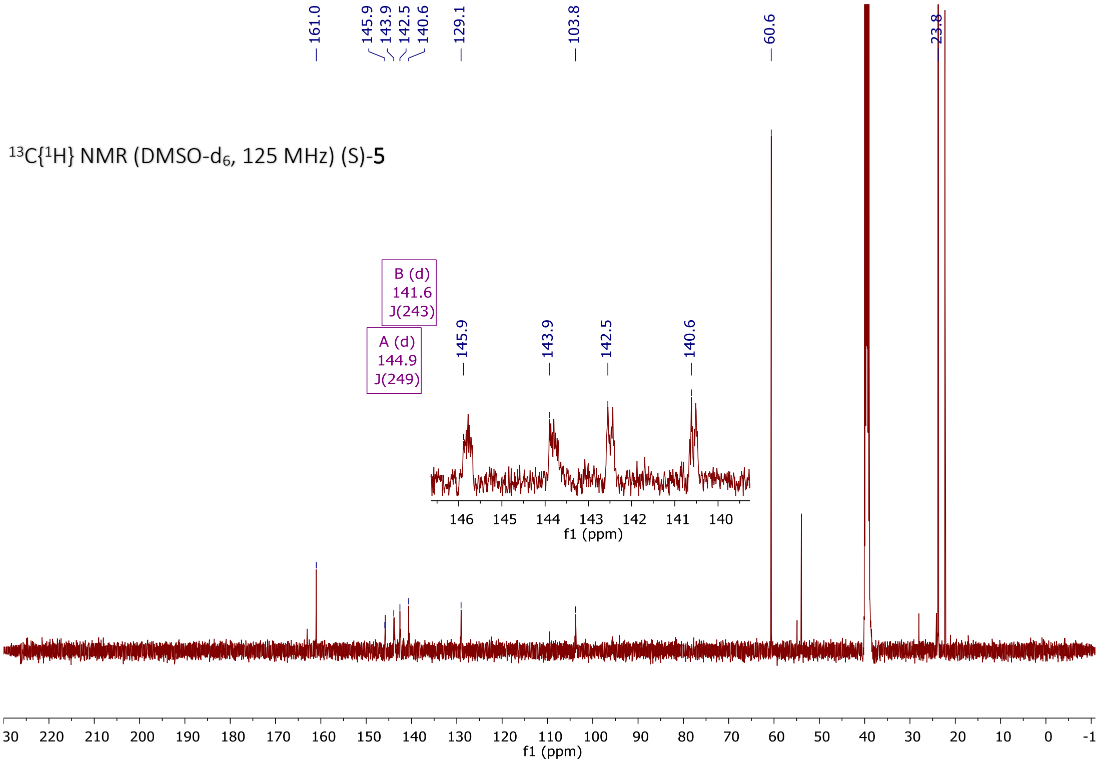

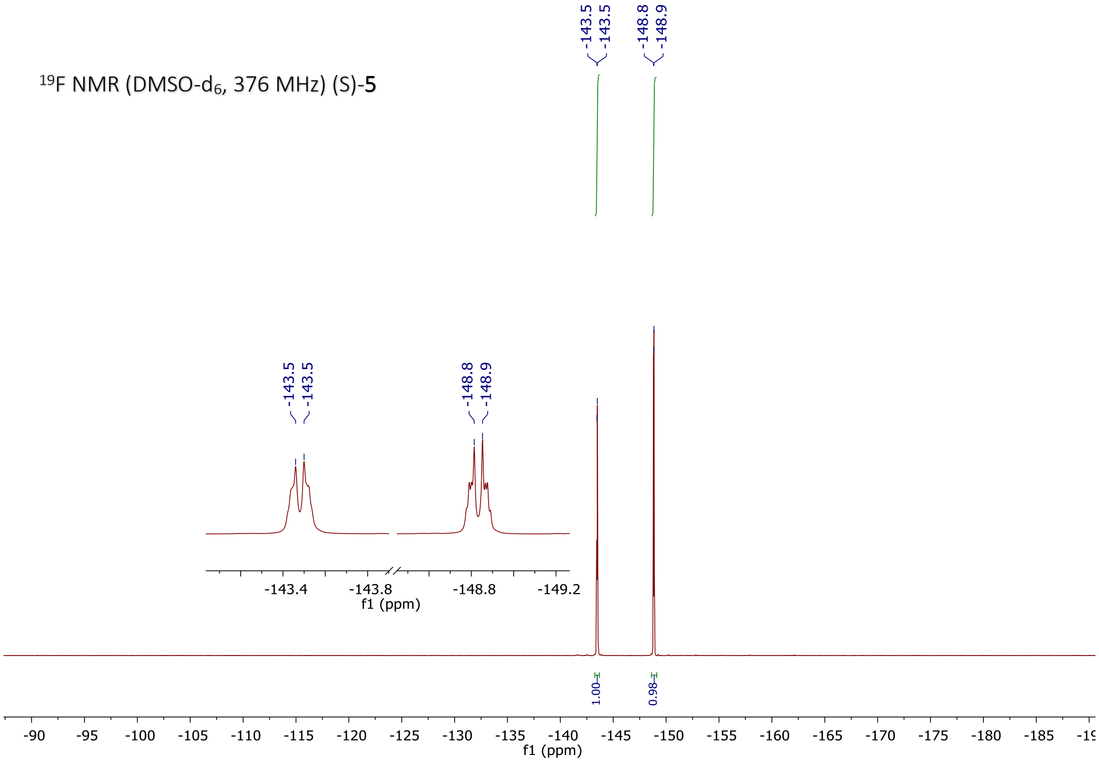

$^{19}$F NMR (DMSO-d$_6$, 376 MHz) (S)-**5**
-143.5
-143.5
-148.8
-148.9
1.00
0.98
-143.4
-143.8
-148.8
-149.2
f1 (ppm)


## Compound (*S*)-**7**

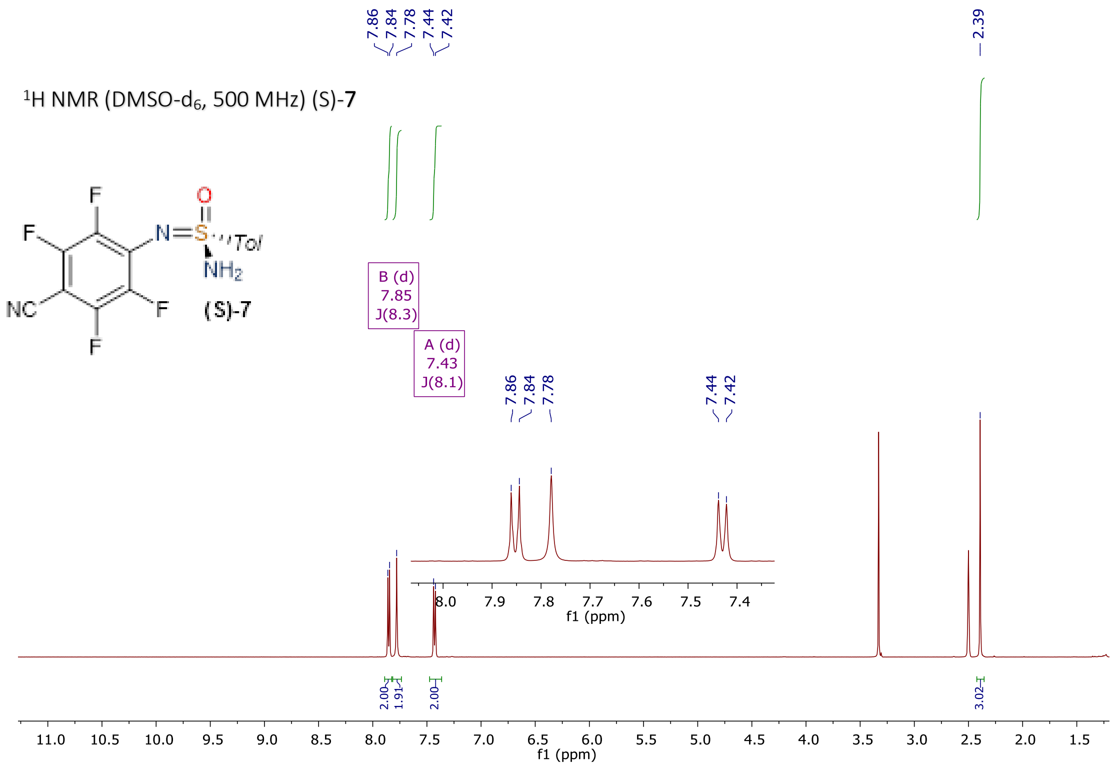

$^1$H NMR (DMSO-d$_6$, 500 MHz) (S)-**7**
(S)-7
7.86
7.84
7.78
7.44
7.42
2.39
B (d)
7.85
J(8.3)
A (d)
7.43
J(8.1)
2.00
1.91
2.00
3.02
f1 (ppm)

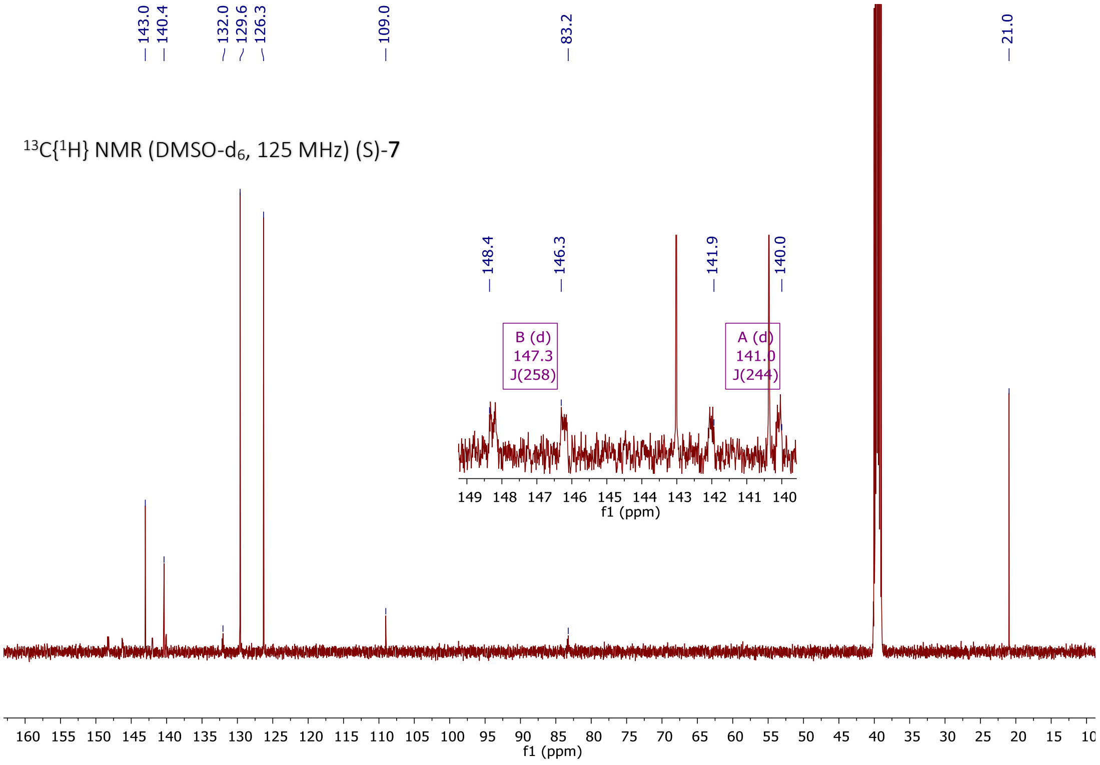

$^{13}C\{^1H\}$ NMR (DMSO-d$_6$, 125 MHz) (S)-**7**
143.0
140.4
132.0
129.6
126.3
109.0
83.2
21.0
148.4
146.3
141.9
140.0
B (d)
147.3
J(258)
A (d)
141.0
J(244)
149 148 147 146 145 144 143 142 141 140
f1 (ppm)
160 155 150 145 140 135 130 125 120 115 110 105 100 95 90 85 80 75 70 65 60 55 50 45 40 35 30 25 20 15 10
f1 (ppm)


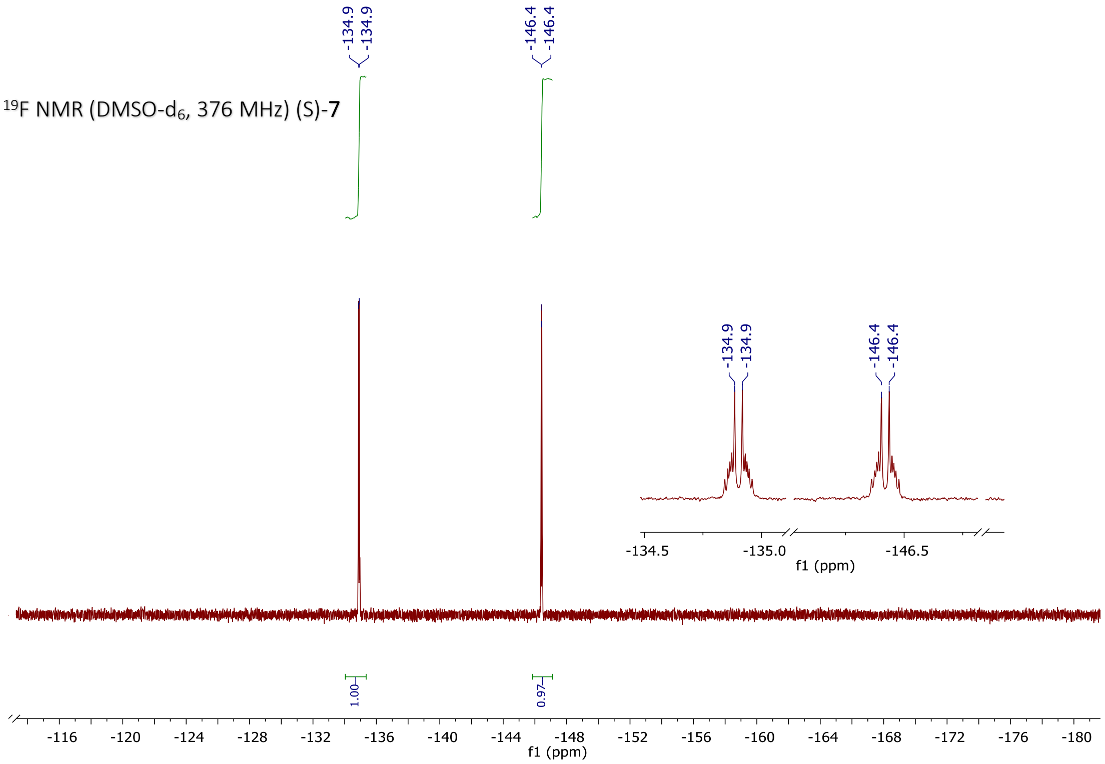

$^{19}F$ NMR (DMSO-d$_6$, 376 MHz) (S)-**7**
-134.9
-134.9
-146.4
-146.4
-134.5
-135.0
-146.5
f1 (ppm)
1.00
0.97
-116 -120 -124 -128 -132 -136 -140 -144 -148 -152 -156 -160 -164 -168 -172 -176 -180
f1 (ppm)

## Compound *Rac*-**8**

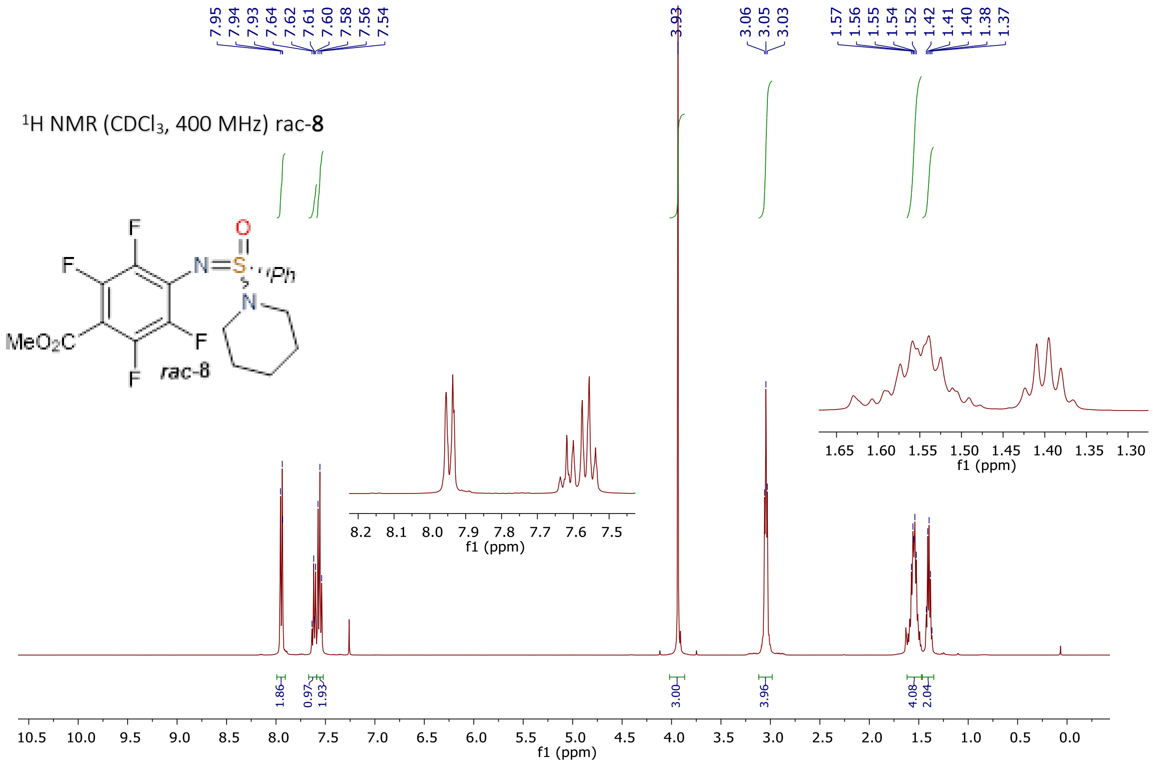


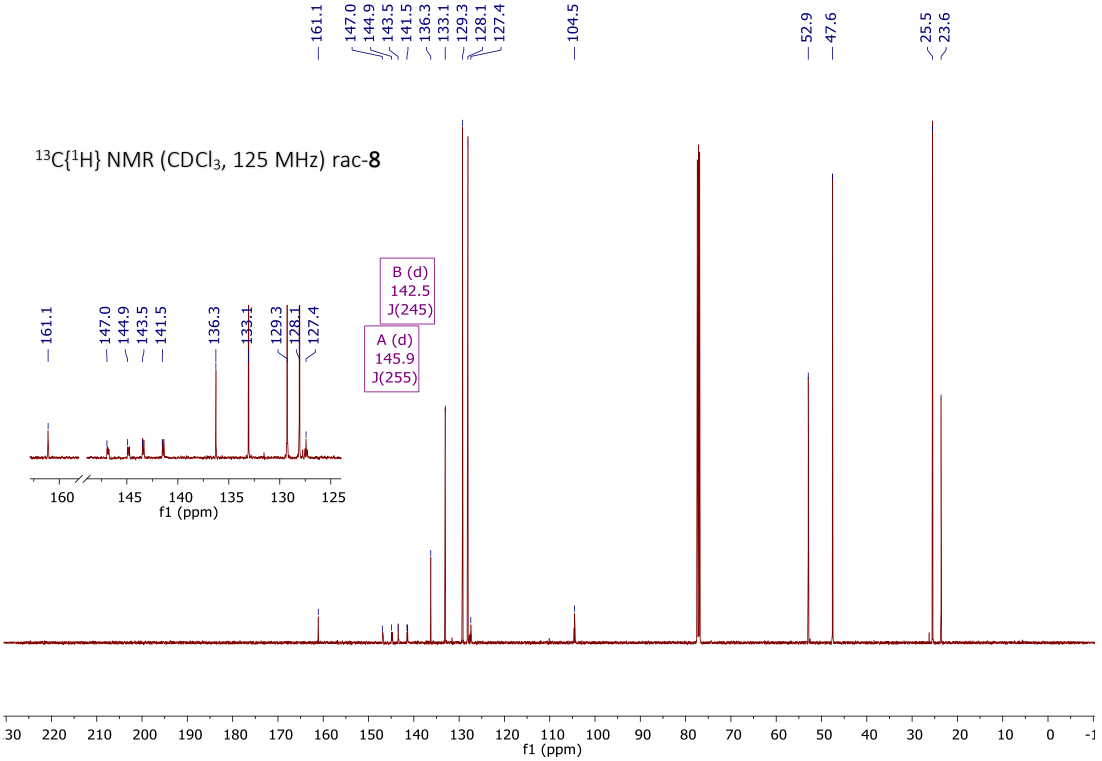

$^{19}F$ NMR ($CDCl_3$, 376 MHz) rac-**8**

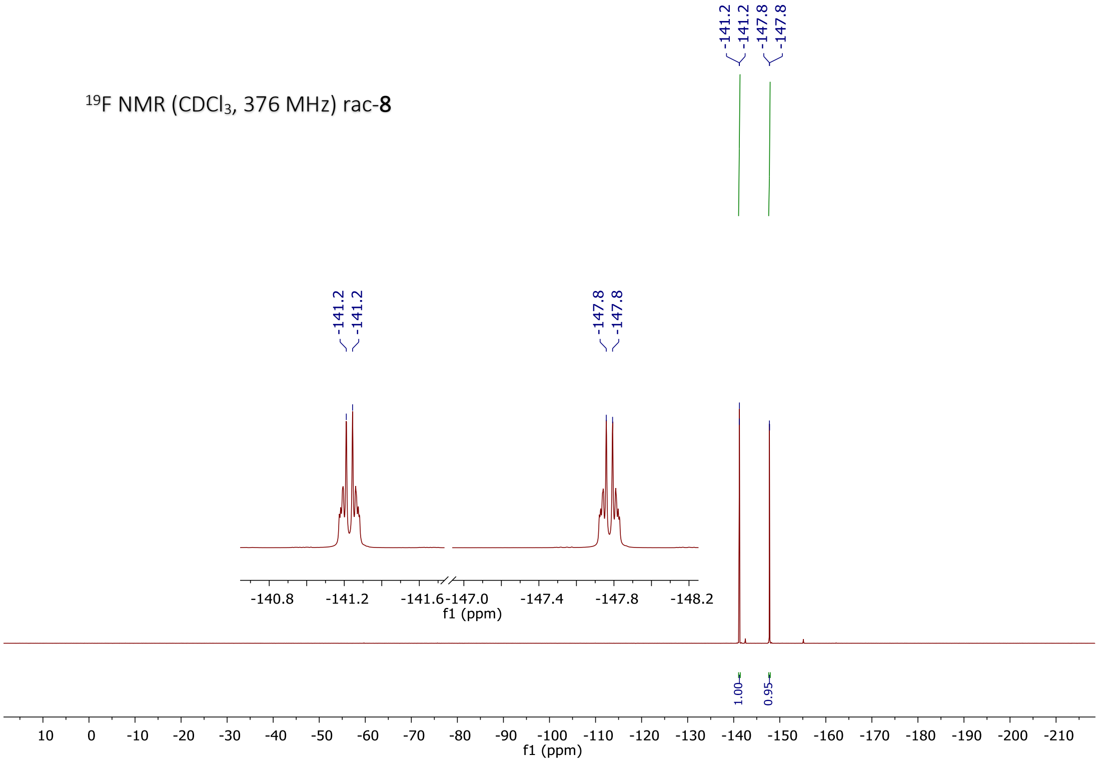


## Compound **9**

$^{1}H$ NMR ($CDCl_3$, 400 MHz) **9**

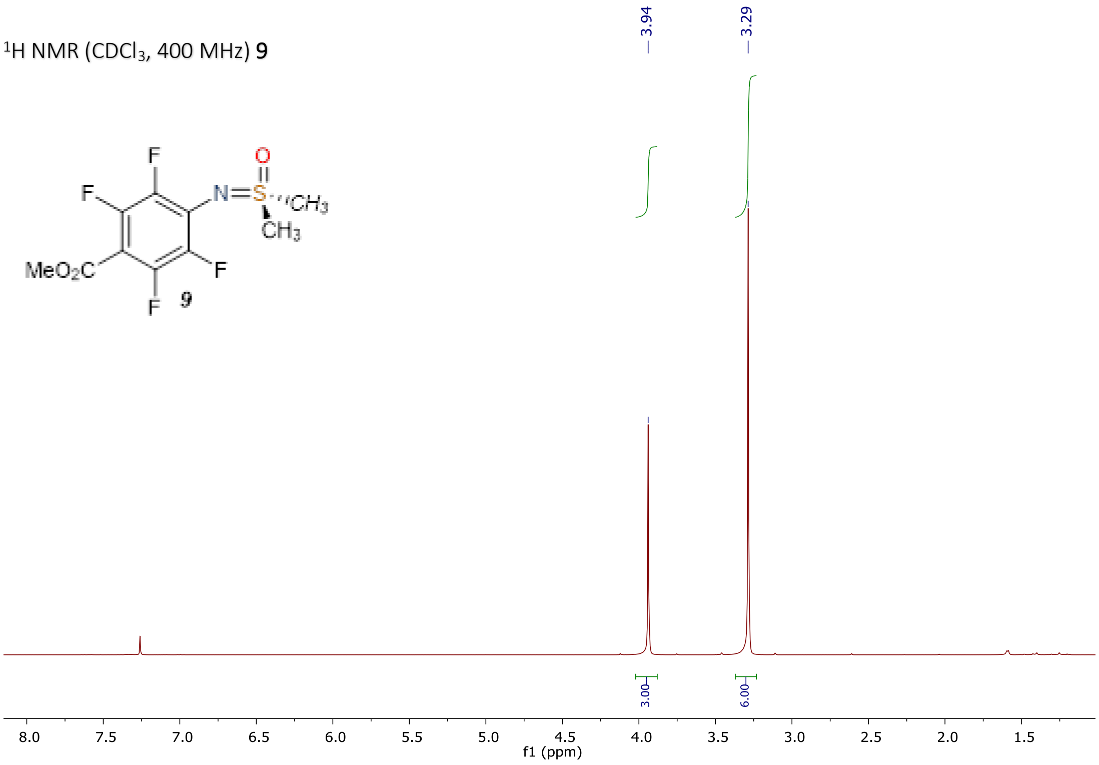

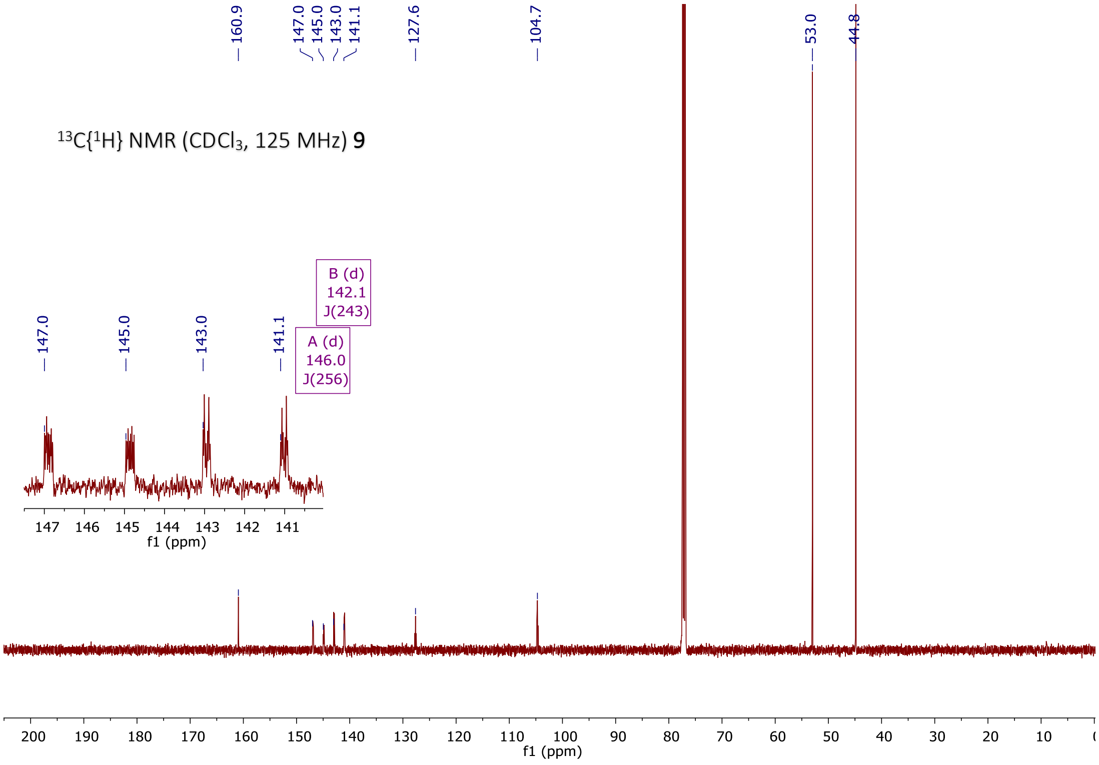

160.9
147.0
145.0
143.0
141.1
127.6
104.7
53.0
44.8
$^{13}C\{^1H\}$ NMR ($CDCl_3$, 125 MHz) 9
B (d)
142.1
J(243)
A (d)
146.0
J(256)
f1 (ppm)


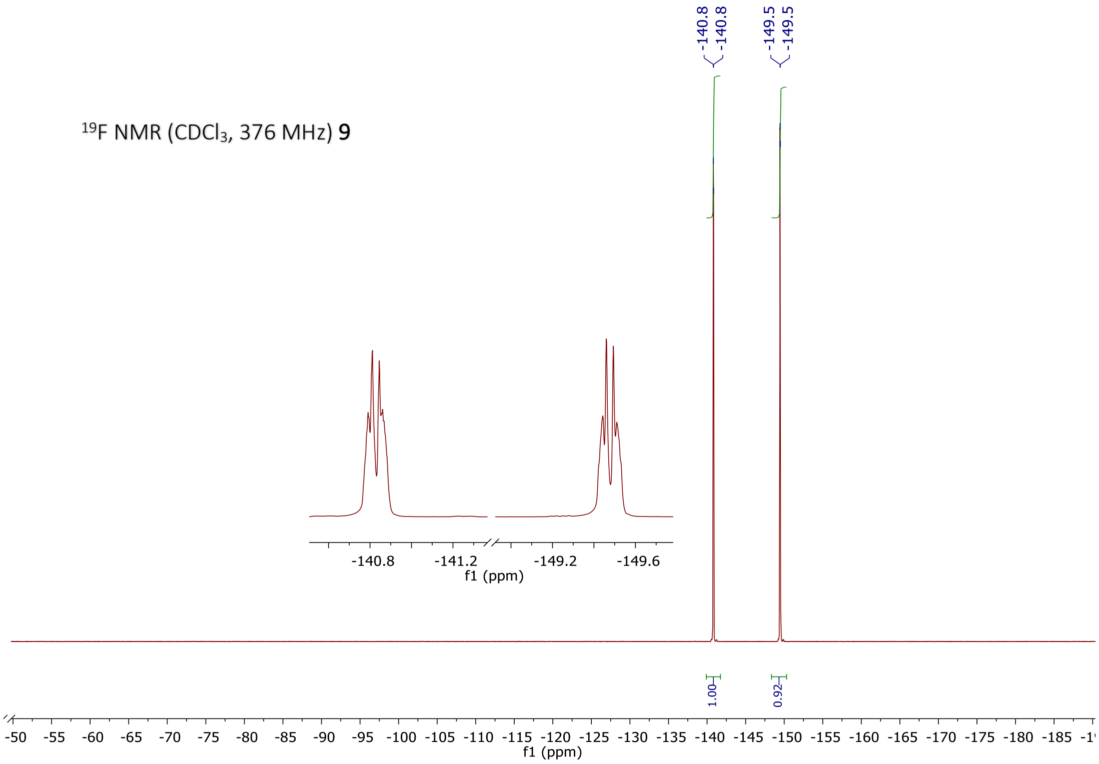

-140.8
-140.8
-149.5
-149.5
$^{19}F$ NMR ($CDCl_3$, 376 MHz) 9
-140.8
-141.2
-149.2
-149.6
f1 (ppm)
1.00
0.92
f1 (ppm)

Compound *rac*-**10**

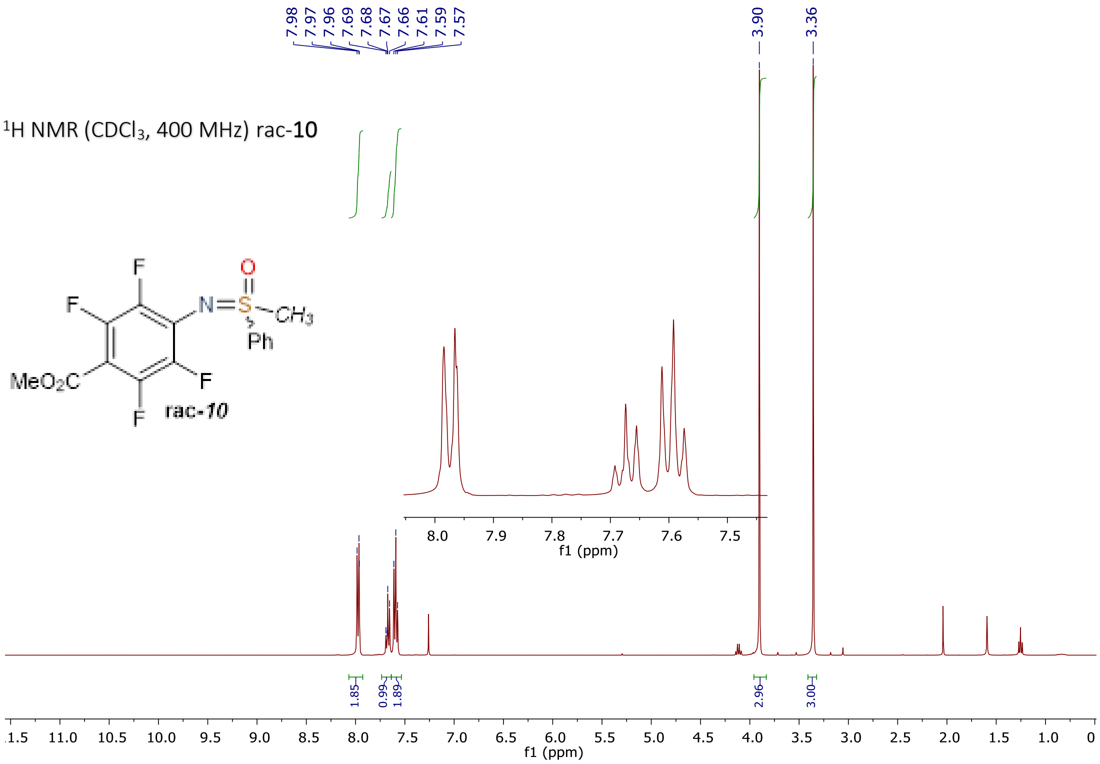


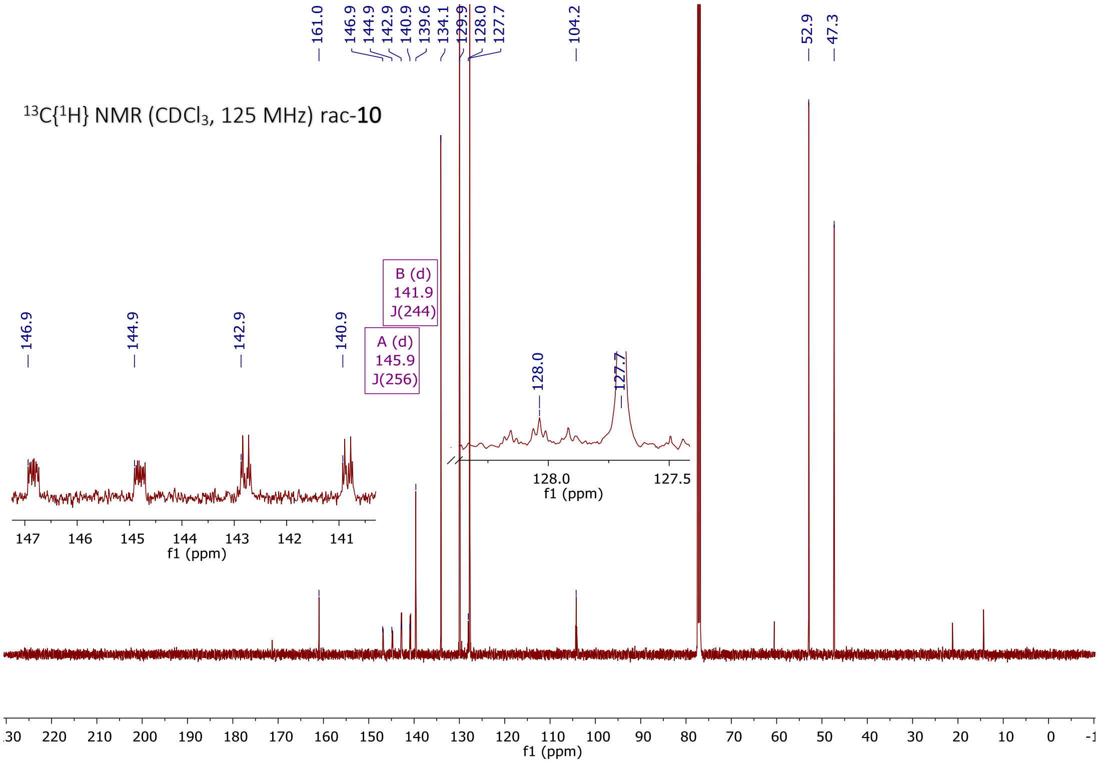

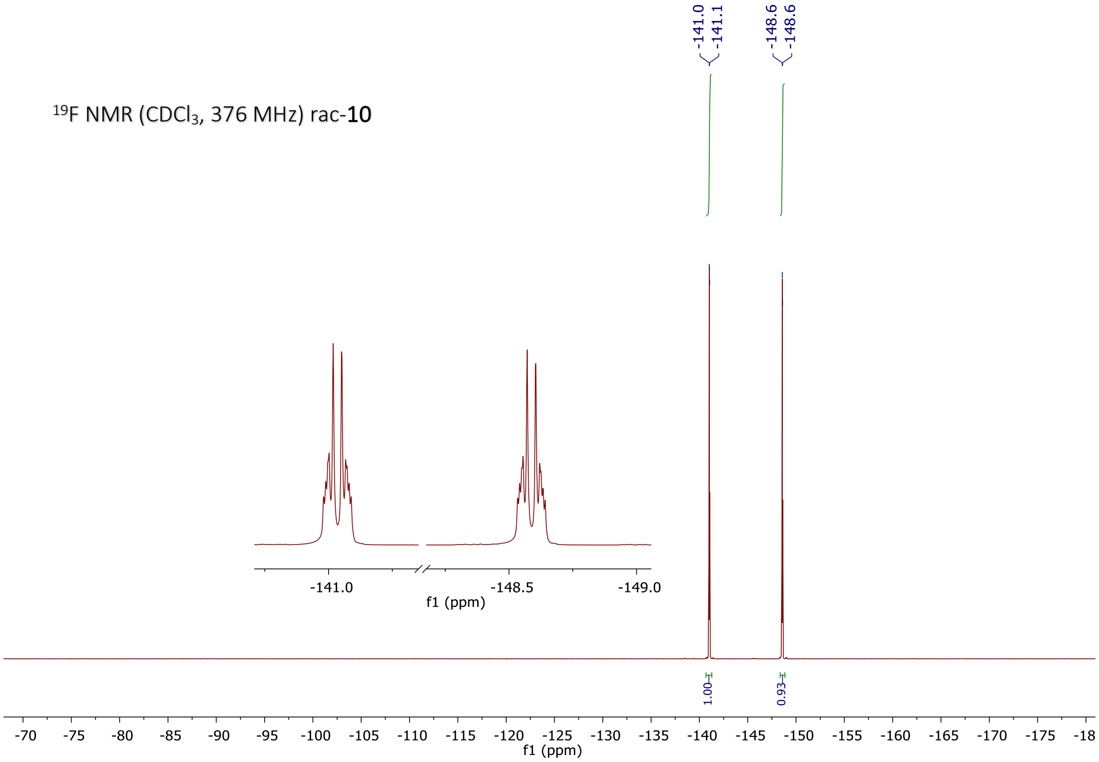


Compound *rac*-**11**

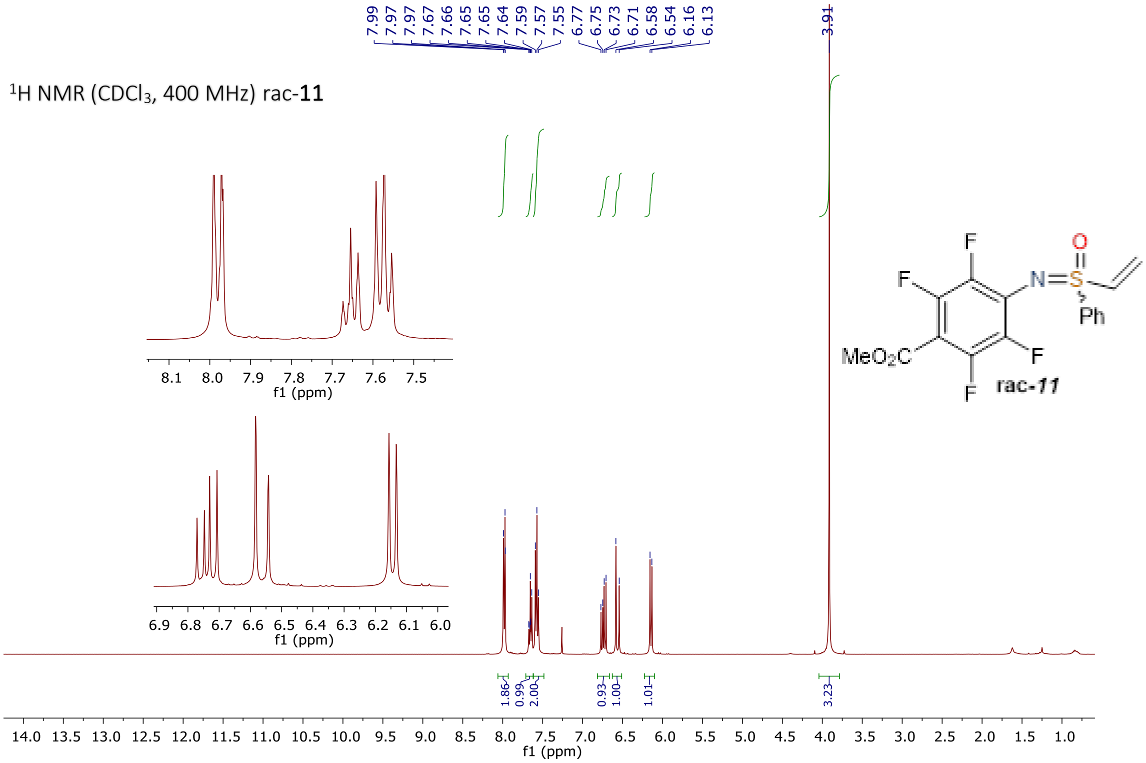

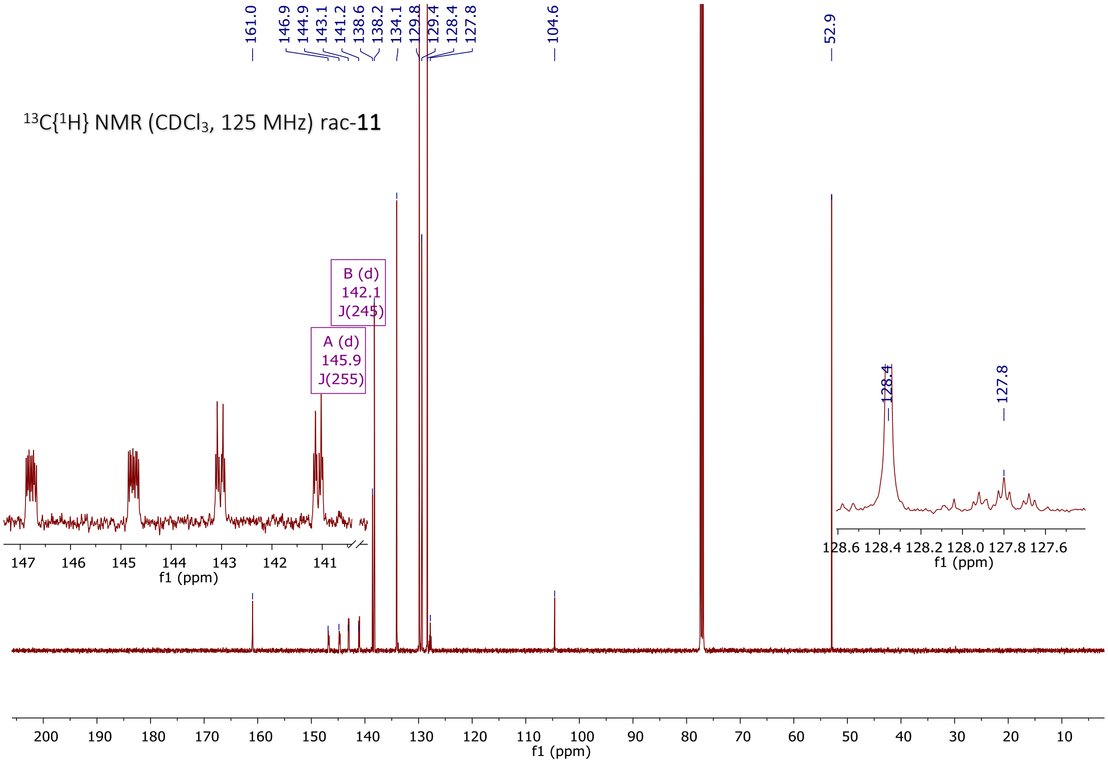

161.0
146.9
144.9
143.1
141.2
138.6
138.2
134.1
129.8
129.4
128.4
127.8
104.6
52.9
$^{13}C\{^1H\}$ NMR ($CDCl_3$, 125 MHz) rac-**11**
B (d)
142.1
J(245)
A (d)
145.9
J(255)
f1 (ppm)


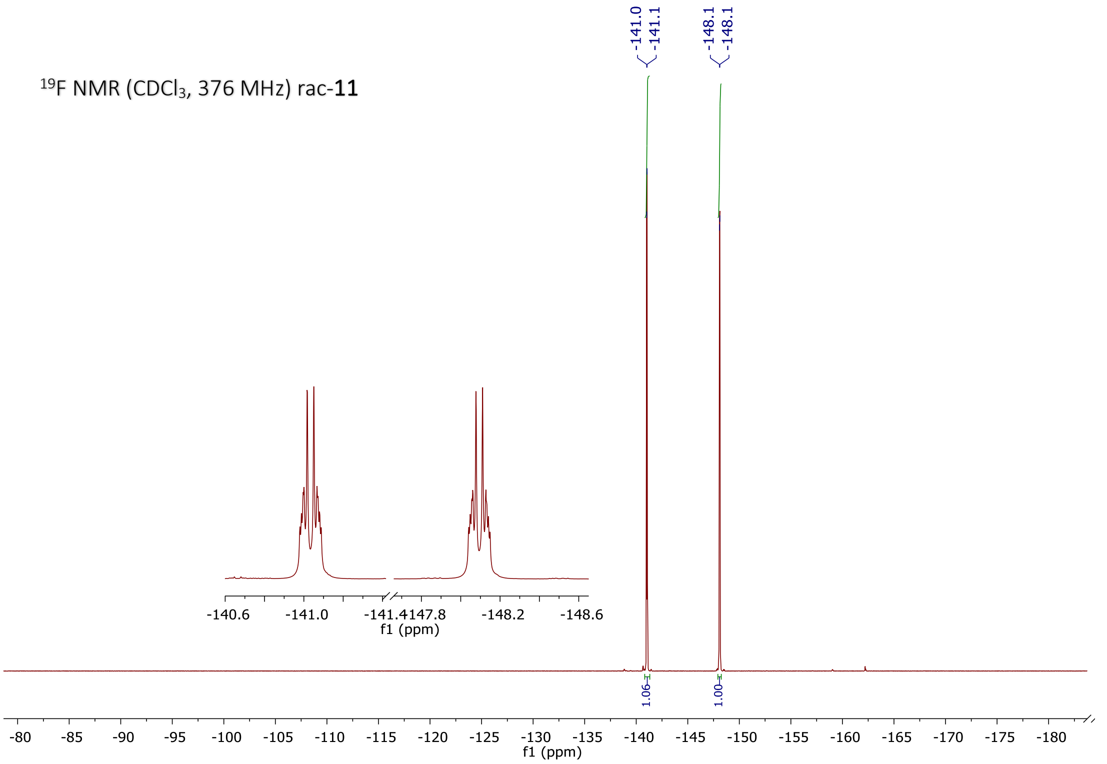

-141.0
-141.1
-148.1
-148.1
$^{19}F$ NMR ($CDCl_3$, 376 MHz) rac-**11**
1.06
1.00
f1 (ppm)

Compound *rac*-**12**

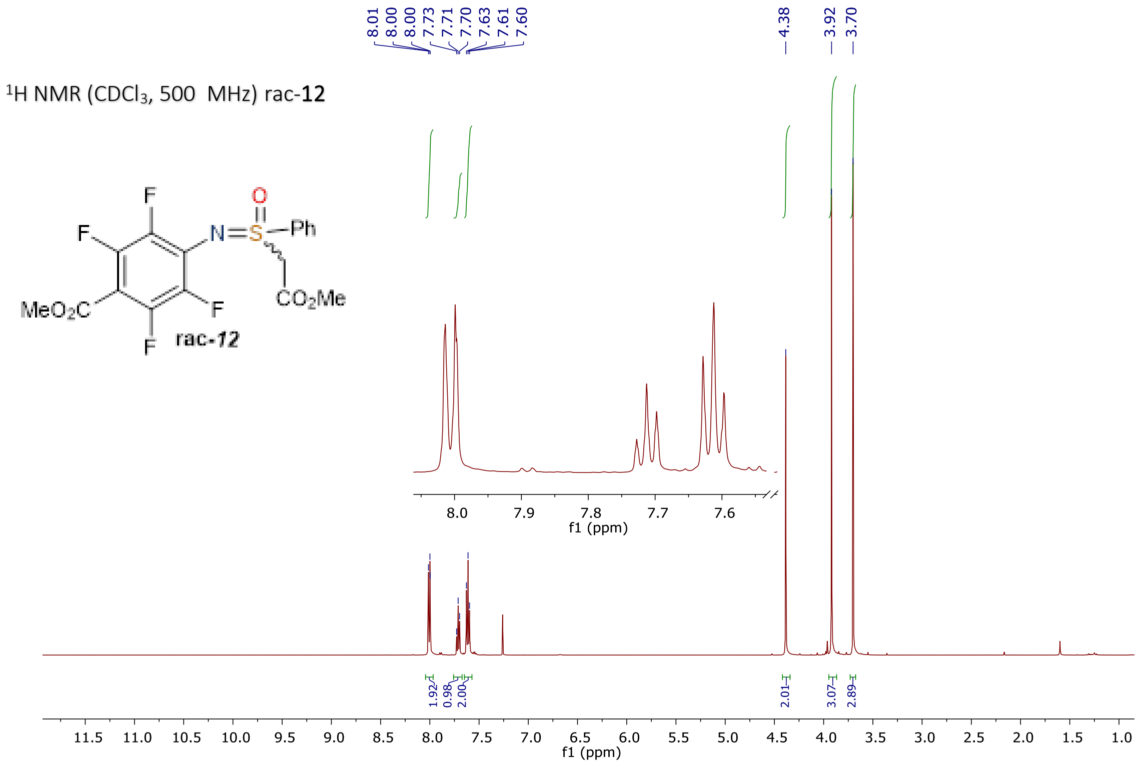


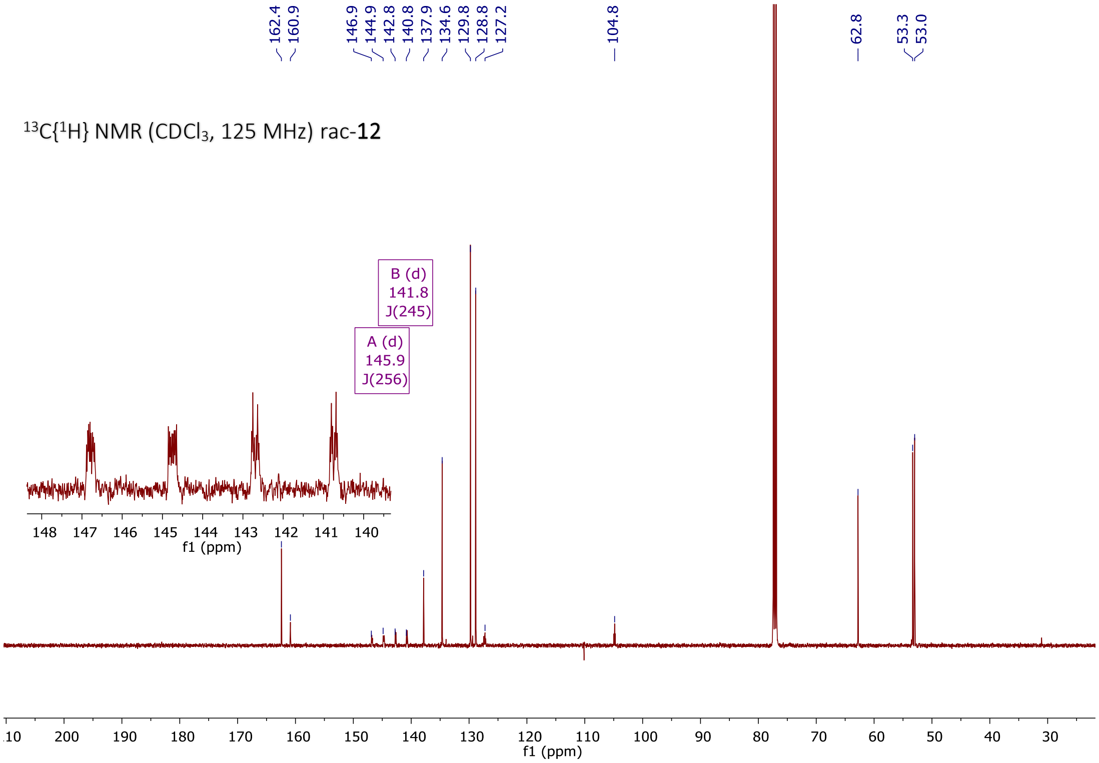

$^{19}F$ NMR ($CDCl_3$, 376 MHz) rac-**12**

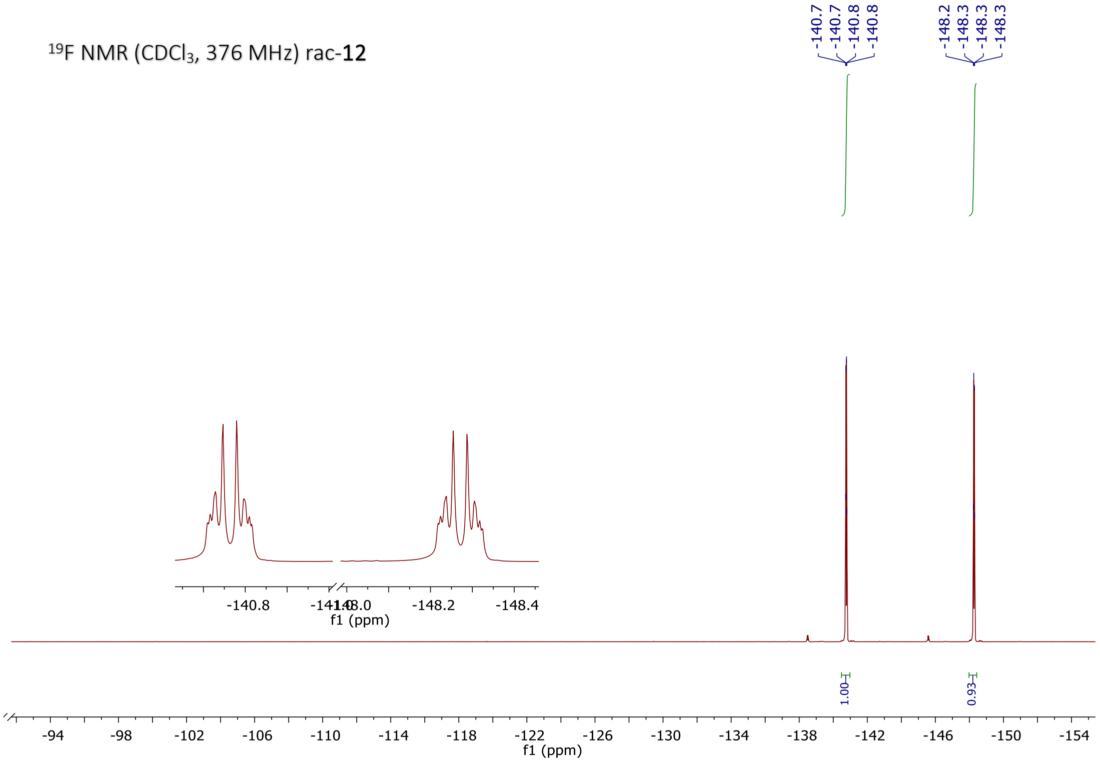


## Compound (*R*)-**13**

$^{1}H$ NMR ($CDCl_3$, 400 MHz) (R)-**13**

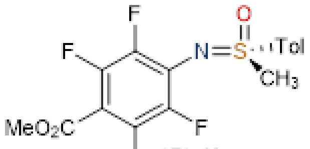

(*R*)-**13**

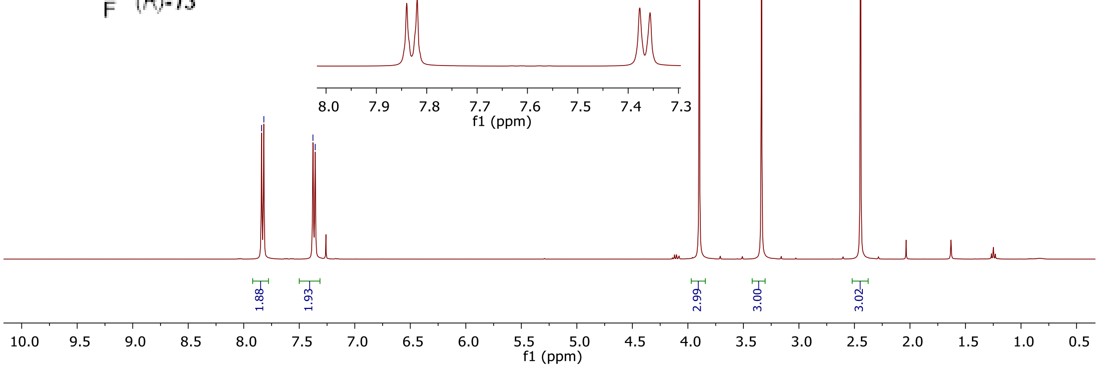

$^{13}C\{^{1}H\}$ NMR ($CDCl_3$, 125 MHz) (R)-**13**

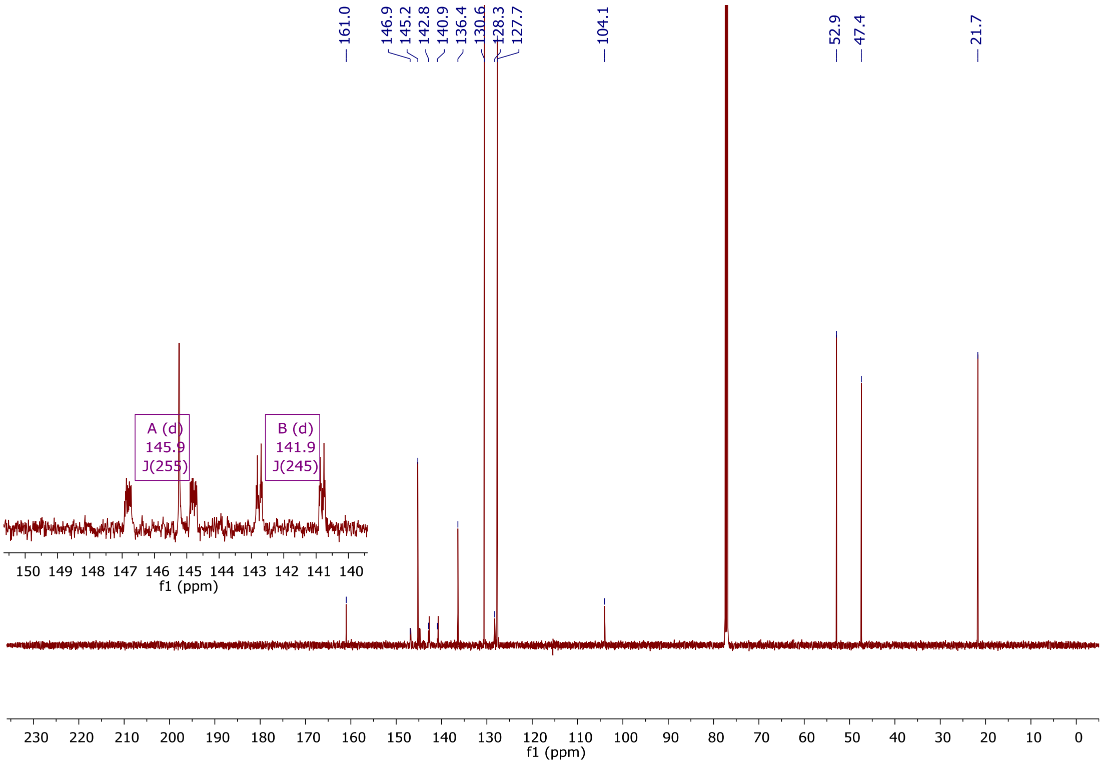


$^{19}F\{^{1}H\}$ NMR ($CDCl_3$, 376 MHz) (R)-**13**

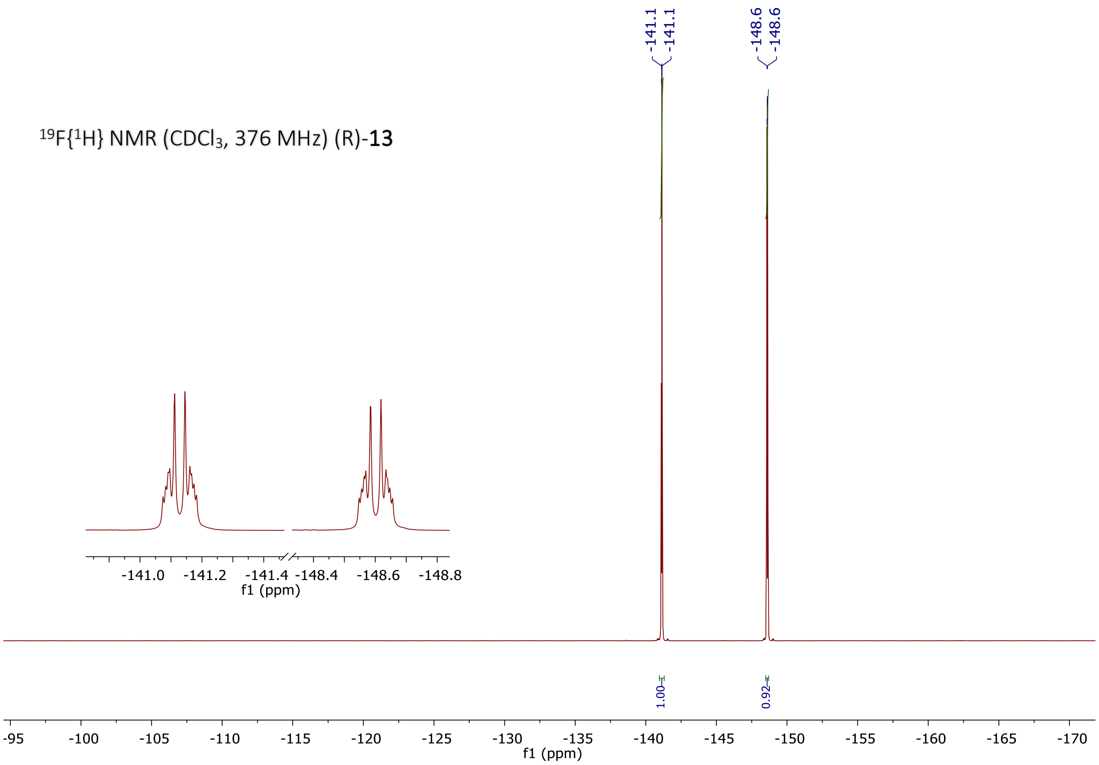

## Compound *rac*-**14**

$^1$H NMR ($CDCl_3$, 500 MHz) rac-**14**

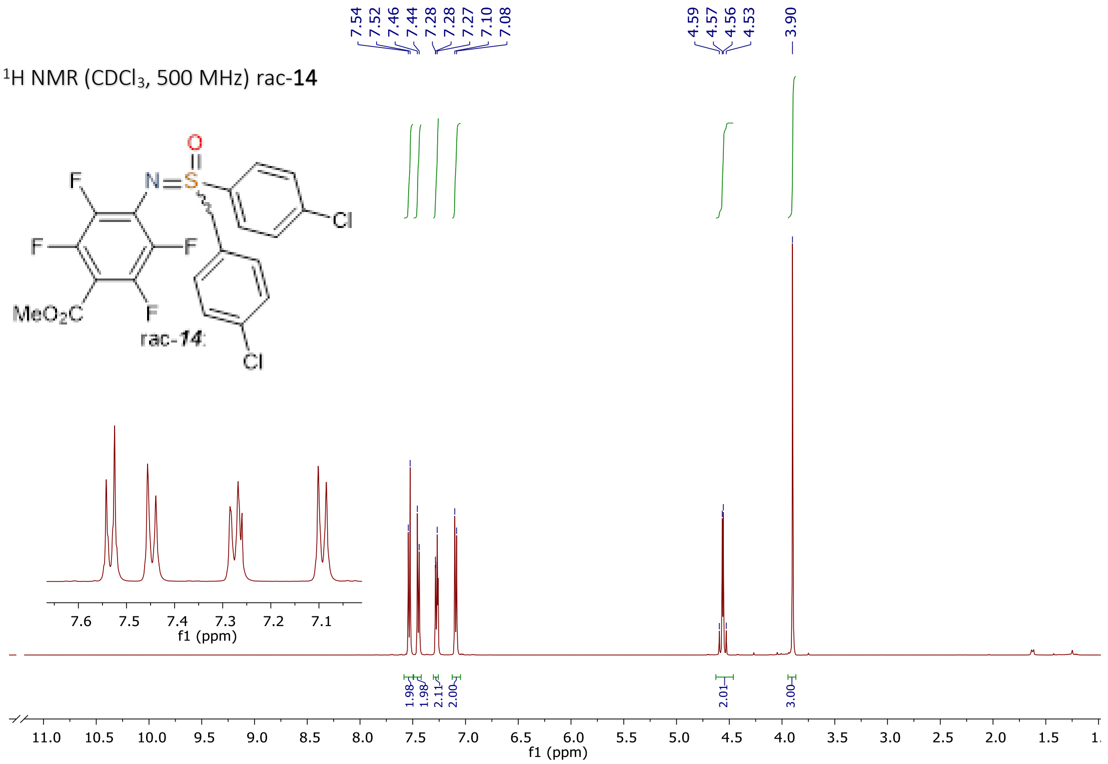


$^{13}C\{^1H\}$ NMR ($CDCl_3$, 125 MHz) rac-**14**

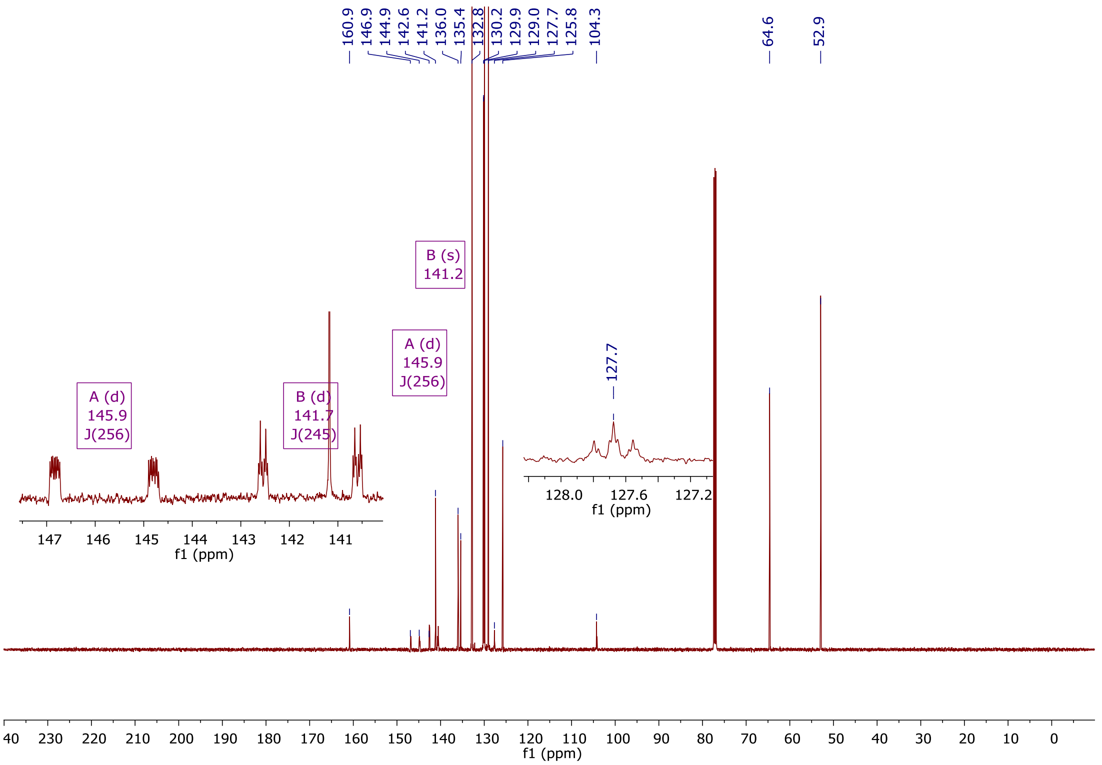

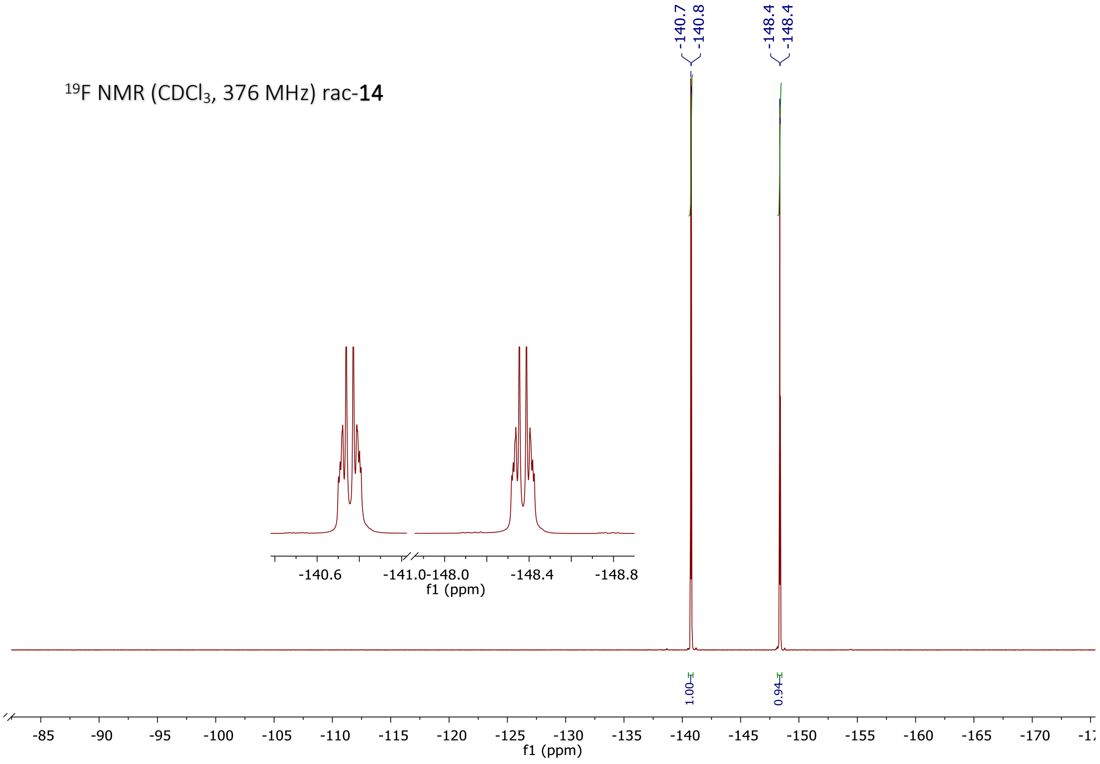

$^{19}$F NMR (CDCl$_3$, 376 MHz) rac-**14**
-140.7
-140.8
-148.4
-148.4
1.00
0.94
f1 (ppm)


Compound **15**

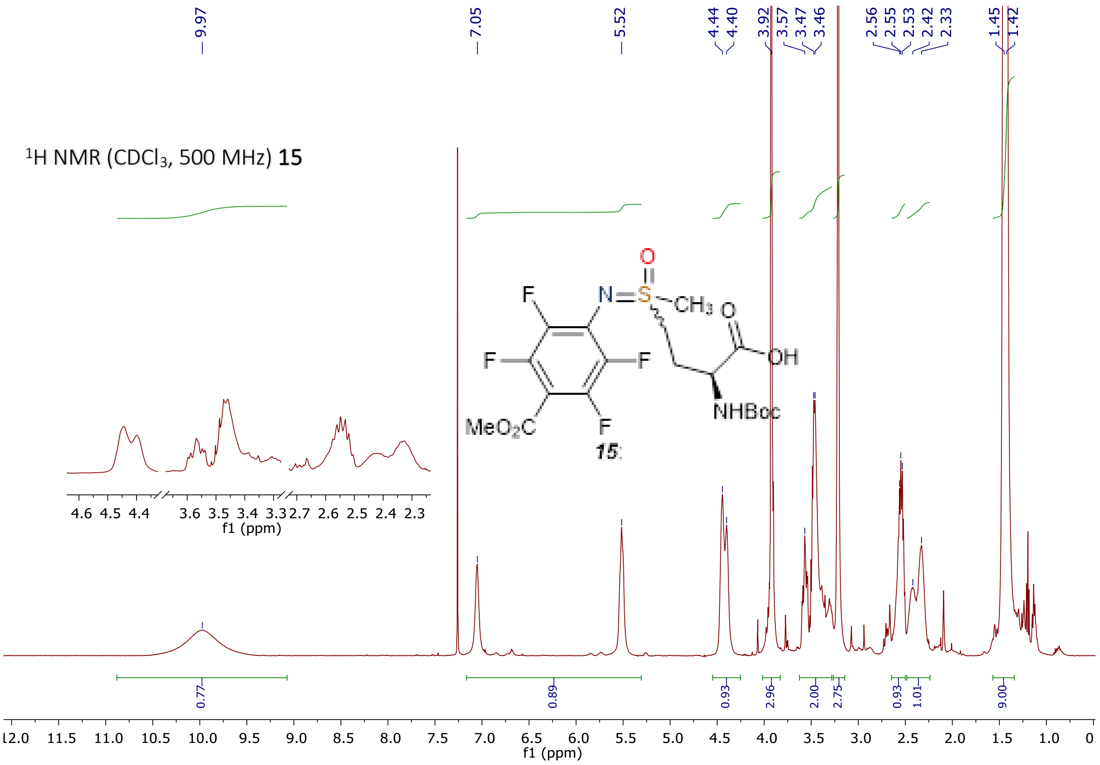

$^{1}$H NMR (CDCl$_3$, 500 MHz) **15**
9.97
7.05
5.52
4.44
4.40
3.92
3.57
3.47
3.46
2.56
2.55
2.53
2.42
2.33
1.45
1.42
0.77
0.89
0.93
2.96
2.00
2.75
0.93
1.01
9.00
f1 (ppm)
MeO$_2$C
NHBoc
OH
CH$_3$
15

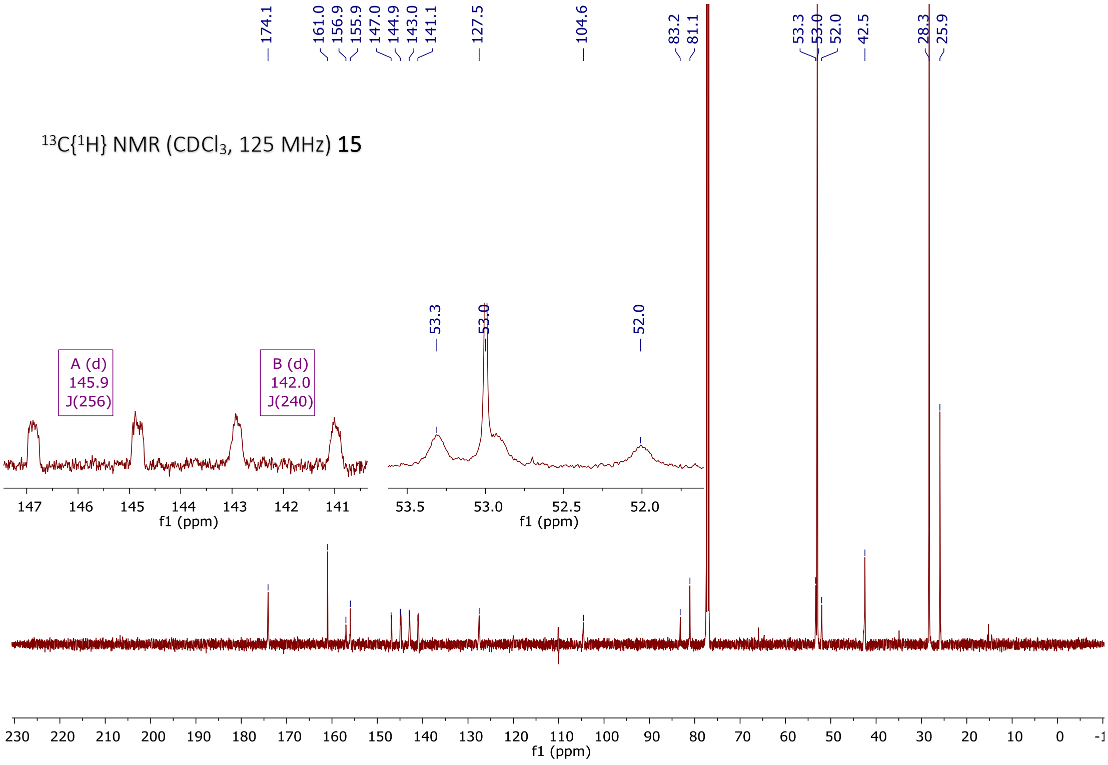


$^{13}C\{^{1}H\}$ NMR ($CDCl_3$, 125 MHz) **15**

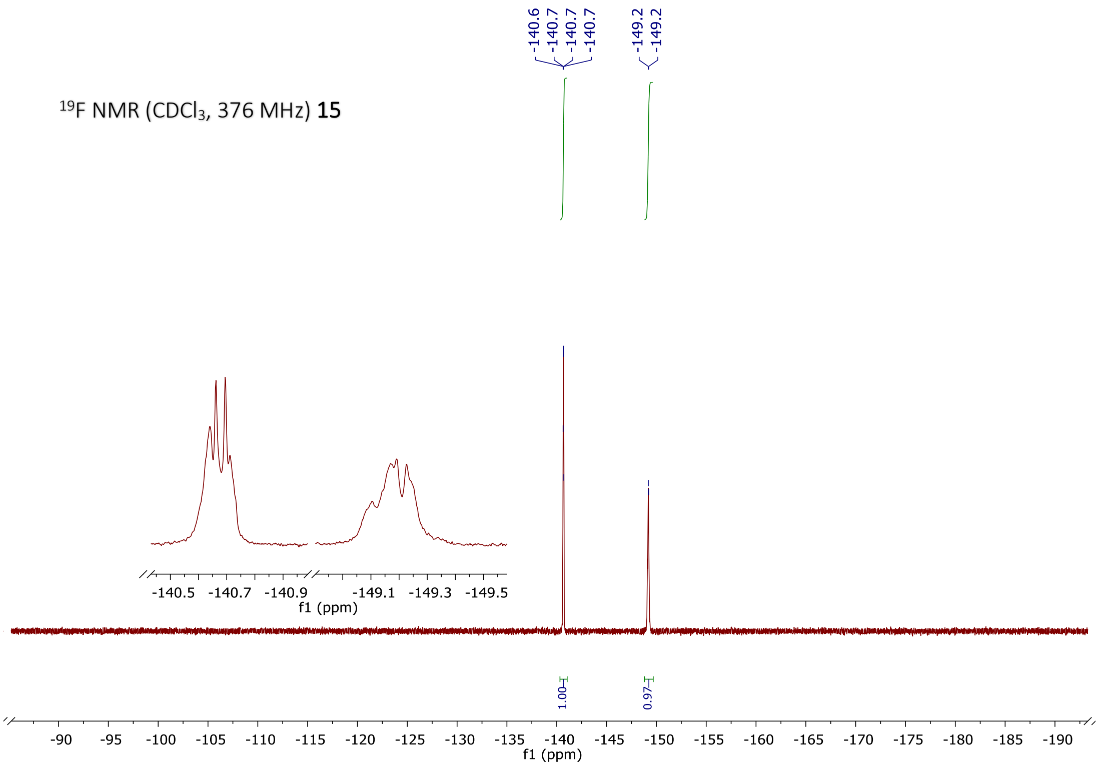


$^{19}F$ NMR ($CDCl_3$, 376 MHz) **15**

Compound *(R)*-**16a**

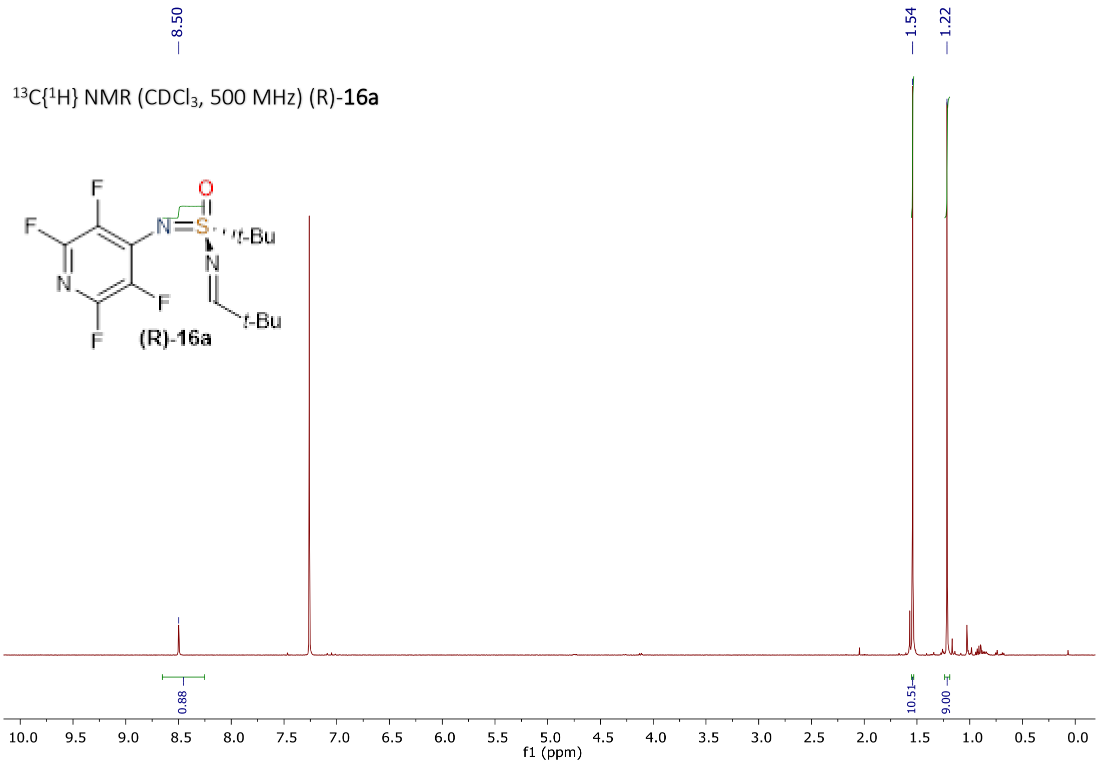


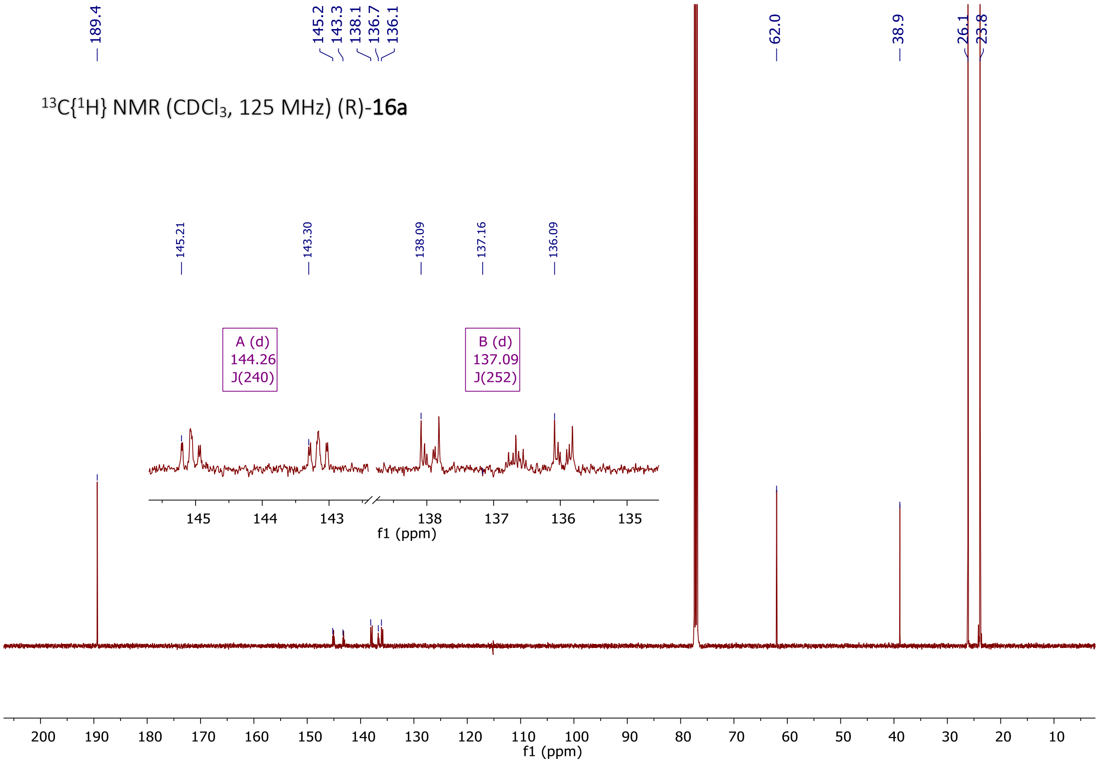

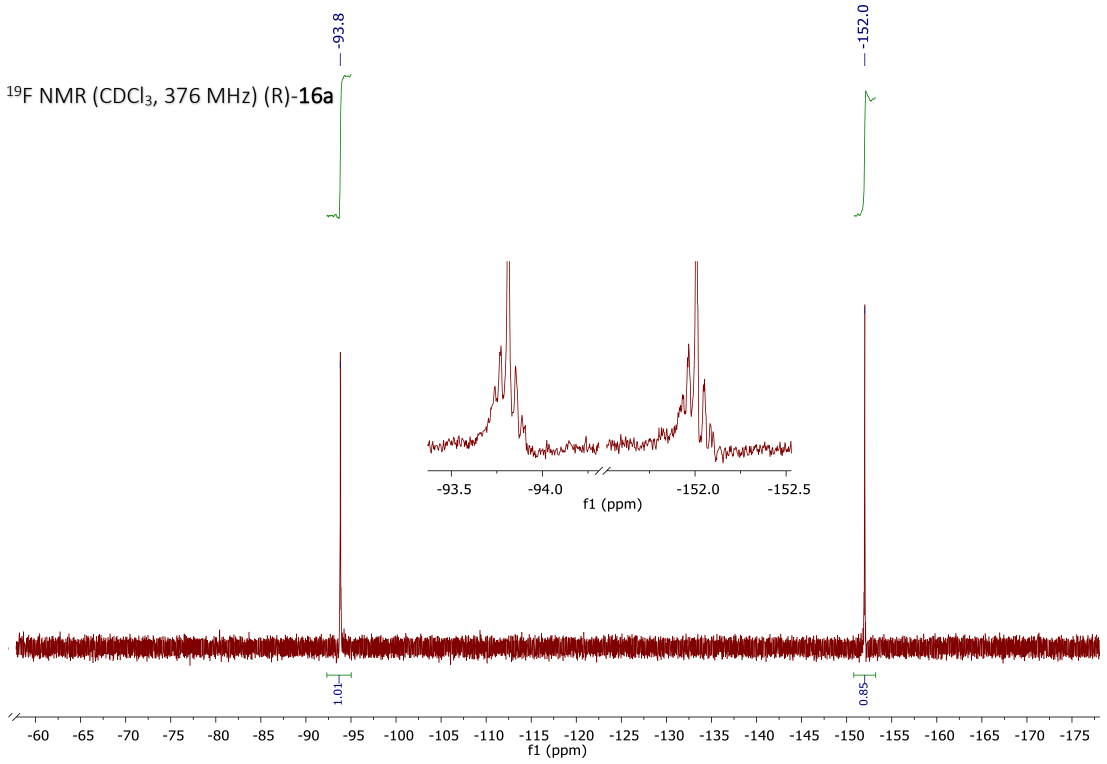


Compound *(R)*-**16b**

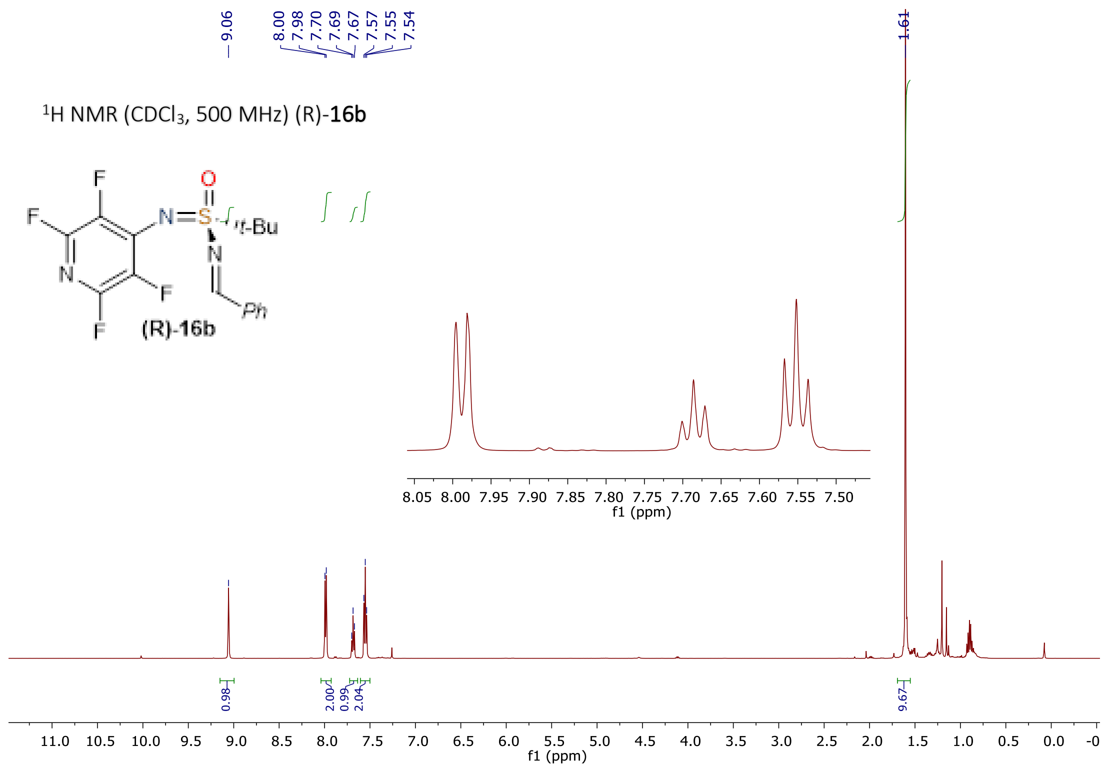

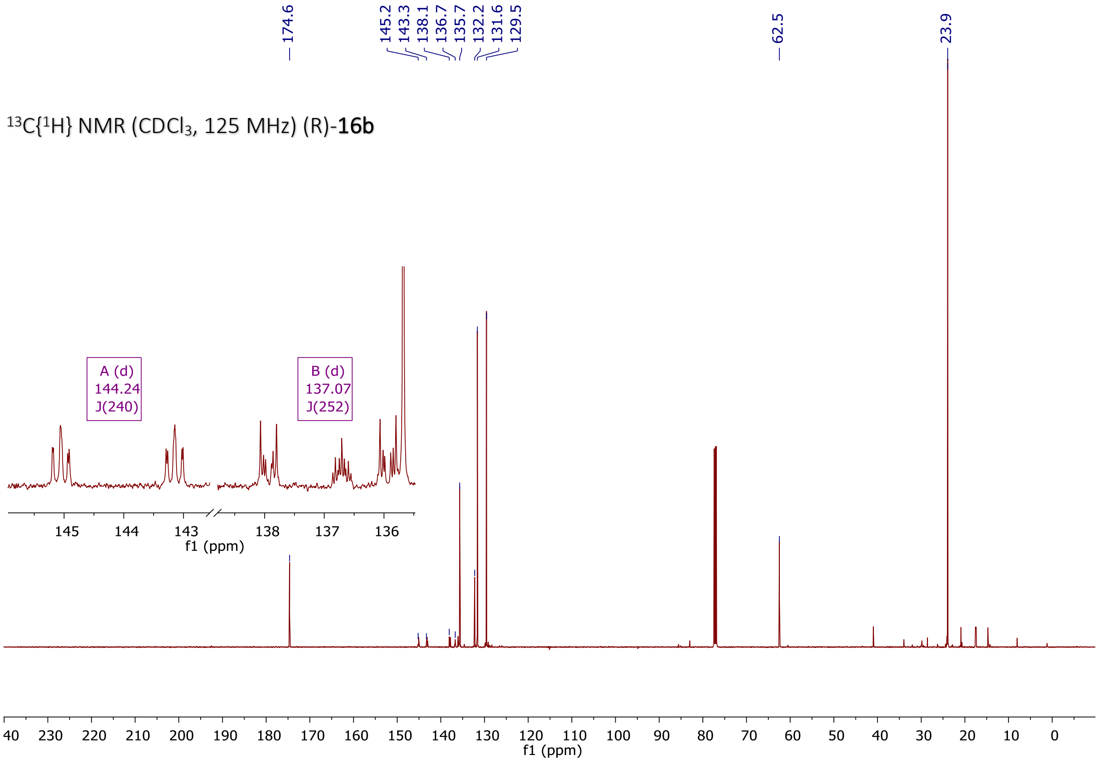
174.6
145.2
143.3
138.1
136.7
135.7
132.2
131.6
129.5
62.5
23.9
$^{13}C\{^{1}H\}$ NMR ($CDCl_3$, 125 MHz) (R)-**16b**
A (d)
144.24
J(240)
B (d)
137.07
J(252)
f1 (ppm)

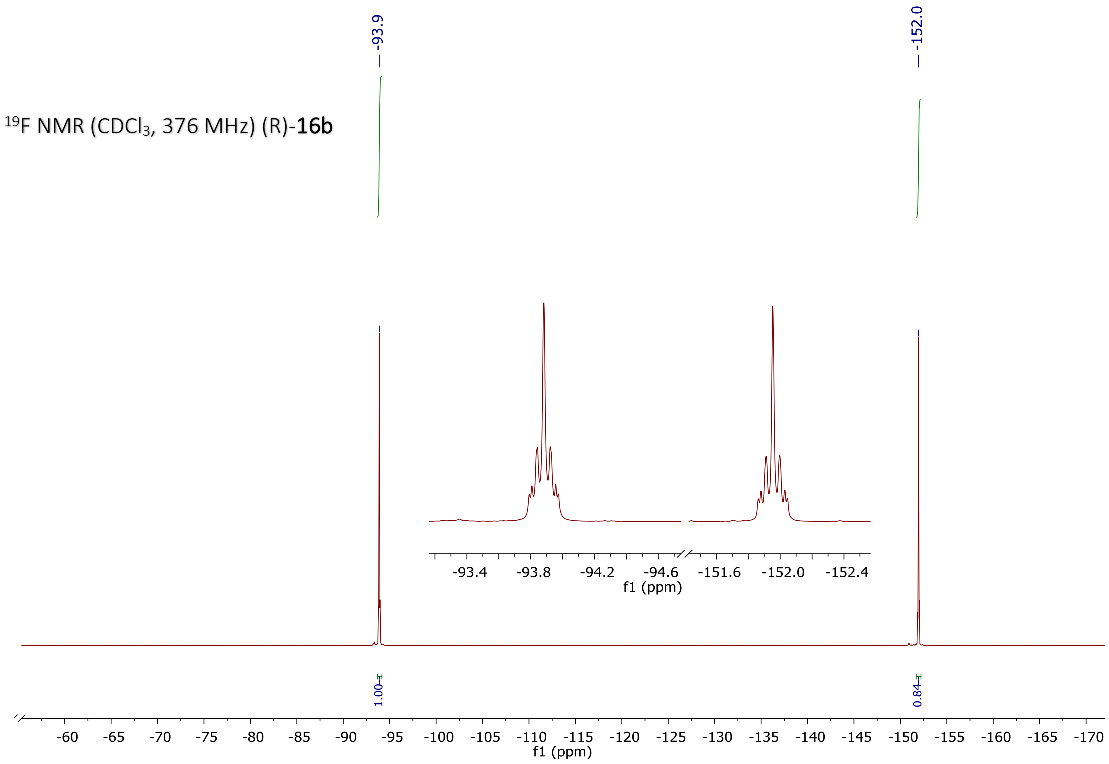
-93.9
-152.0
$^{19}F$ NMR ($CDCl_3$, 376 MHz) (R)-**16b**
1.00
0.84
f1 (ppm)

Compound *(R)*-**16c**

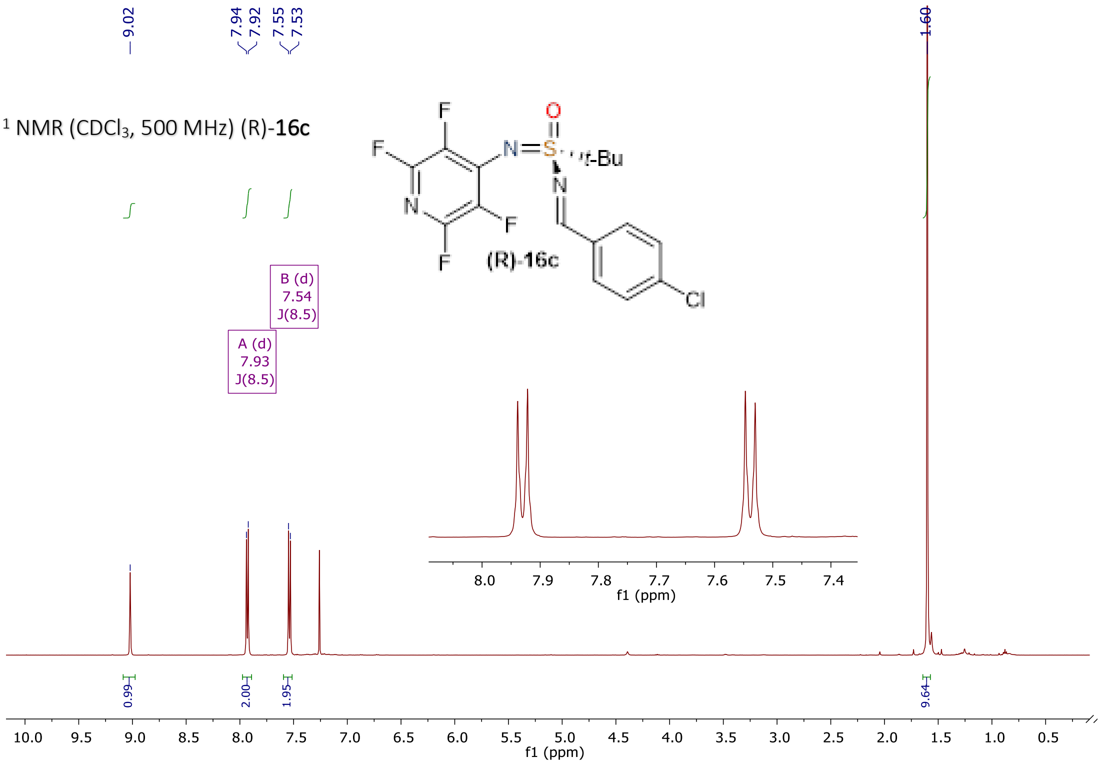


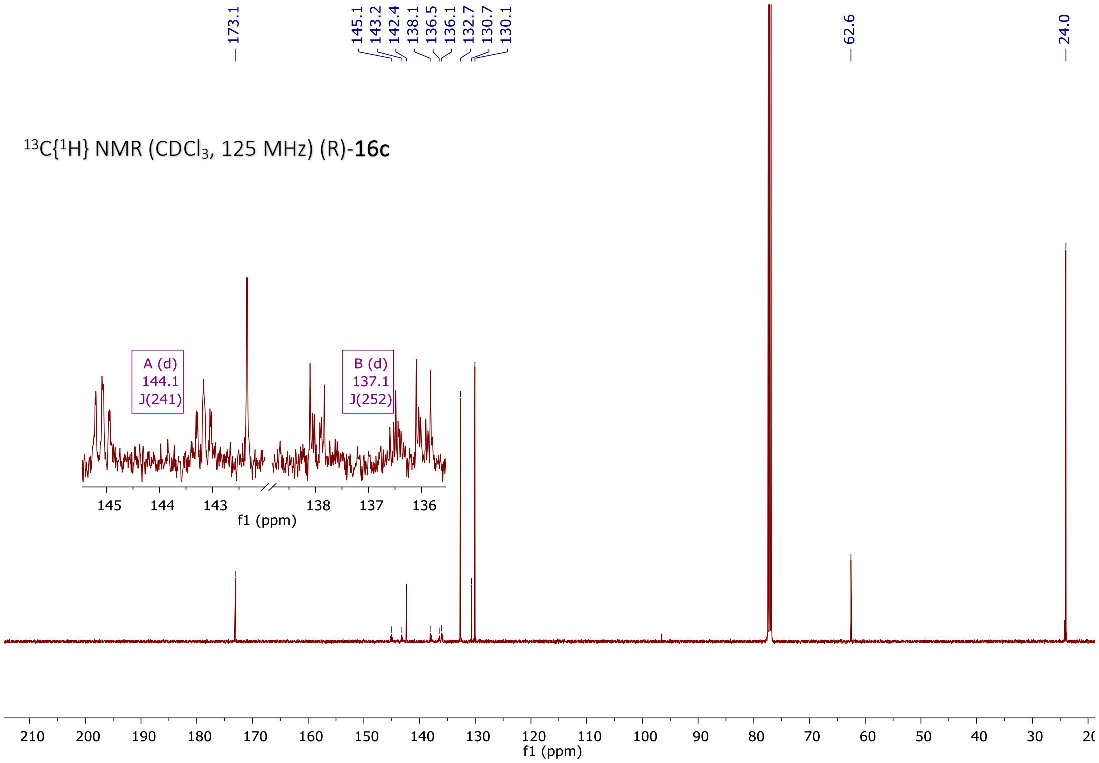

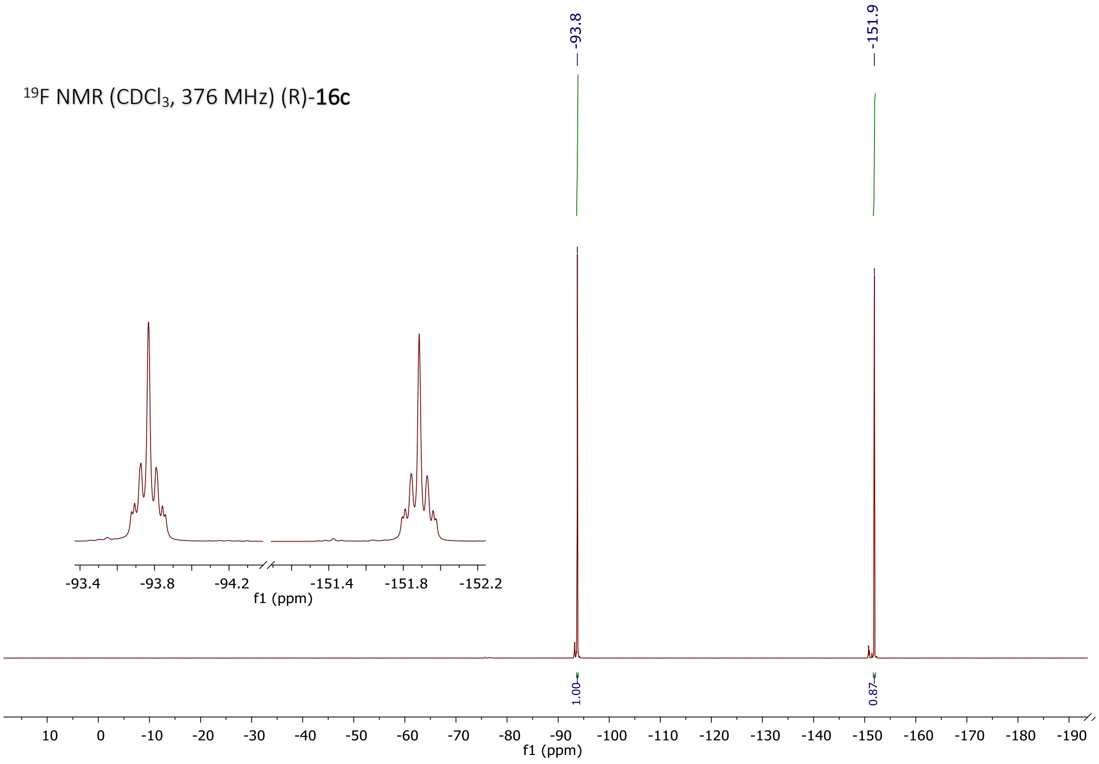

-93.8
-151.9
$^{19}$F NMR (CDCl$_3$, 376 MHz) (R)-**16c**
-93.4 -93.8 -94.2 -151.4 -151.8 -152.2
f1 (ppm)
1.00
0.87
10 0 -10 -20 -30 -40 -50 -60 -70 -80 -90 -100 -110 -120 -130 -140 -150 -160 -170 -180 -190
f1 (ppm)


## Compound **17**

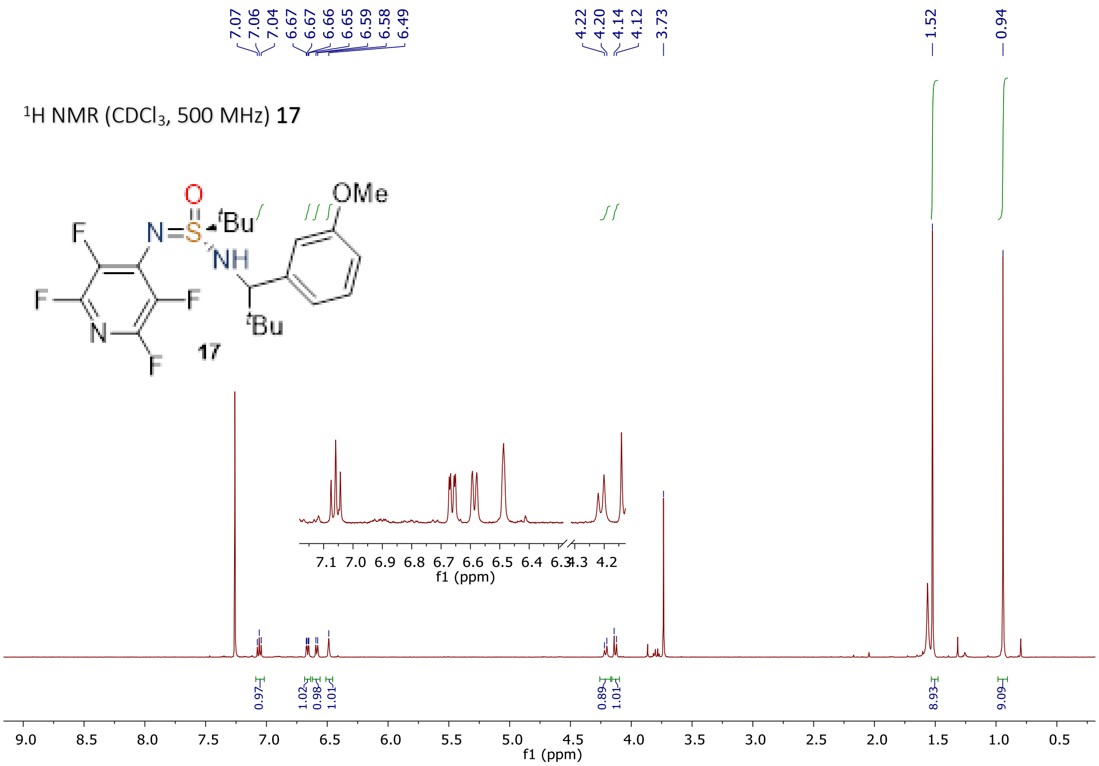

7.07 7.06 7.04 6.67 6.67 6.66 6.65 6.59 6.58 6.49
4.22 4.20 4.14 4.12 3.73
1.52
0.94
$^{1}$H NMR (CDCl$_3$, 500 MHz) **17**
OMe
O
N
S
$^t$Bu
NH
F
F
F
N
F
$^t$Bu
17
7.1 7.0 6.9 6.8 6.7 6.6 6.5 6.4 6.3 4.3 4.2
f1 (ppm)
0.97
1.02 0.98 1.01
0.89 1.01
8.93
9.09
9.0 8.5 8.0 7.5 7.0 6.5 6.0 5.5 5.0 4.5 4.0 3.5 3.0 2.5 2.0 1.5 1.0 0.5
f1 (ppm)

$^{13}C\{^1H\}$ NMR ($CDCl_3$, 125 MHz) **17**

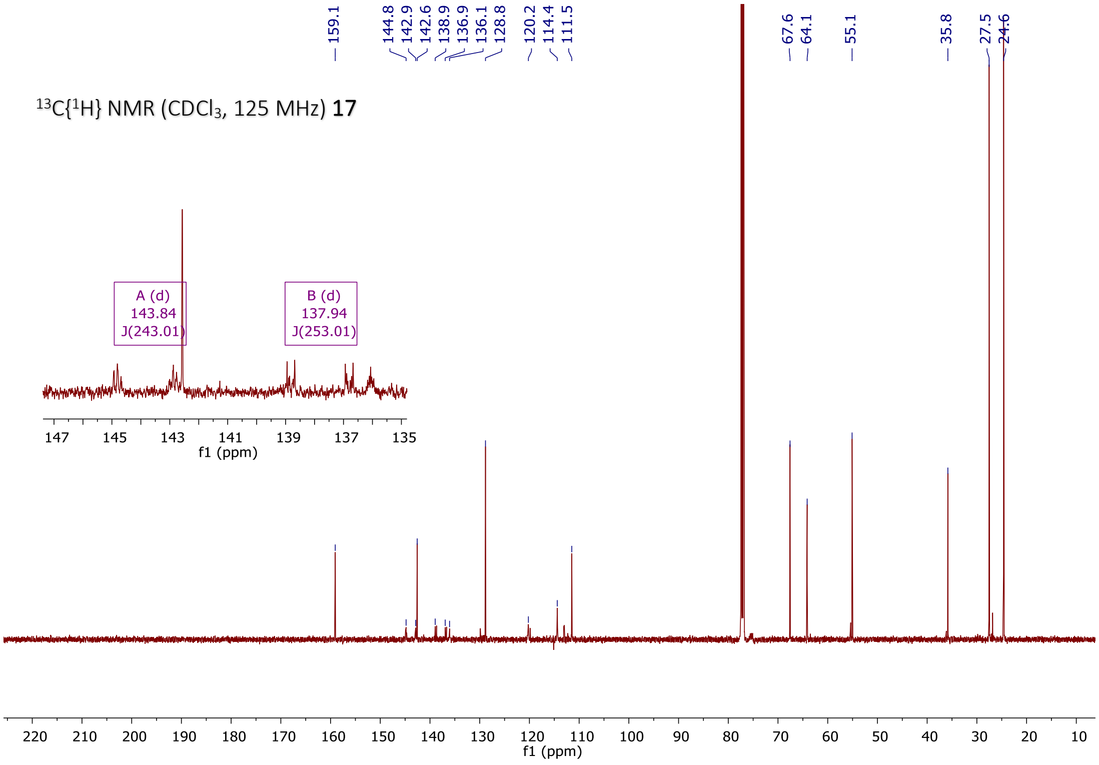


$^{19}F$ NMR ($CDCl_3$, 376 MHz) **17**

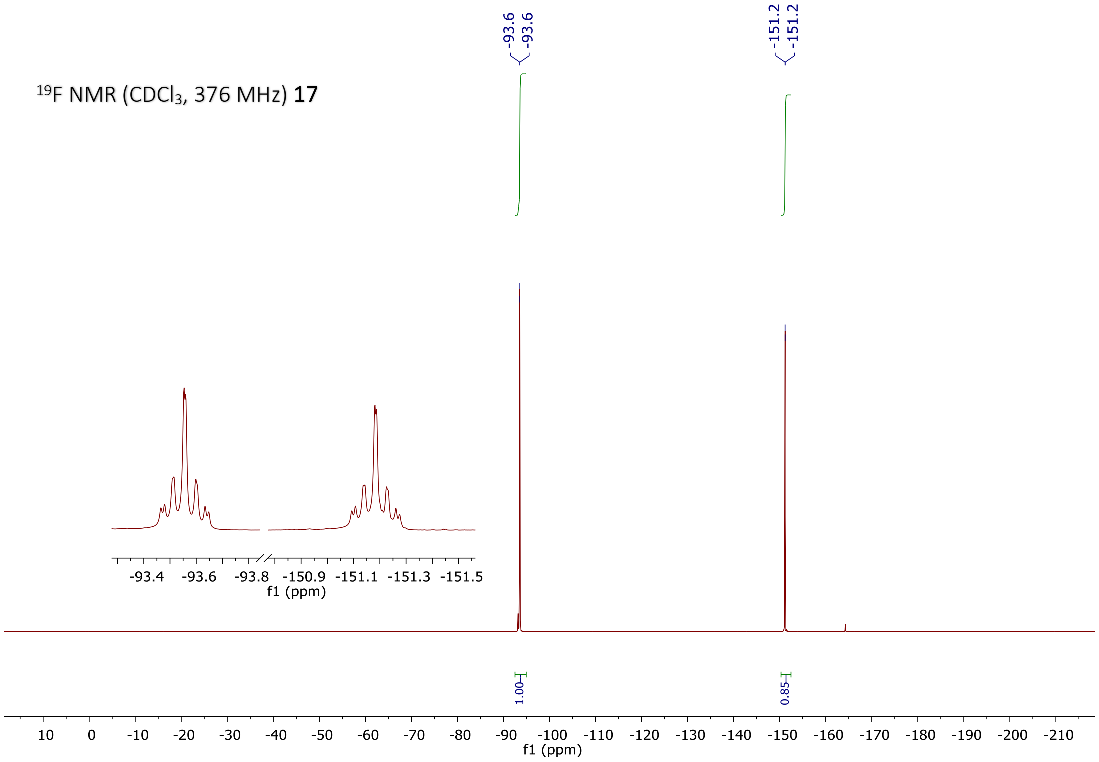

Compound **18**

$^1$H NMR ($CDCl_3$, 500 MHz) **18**

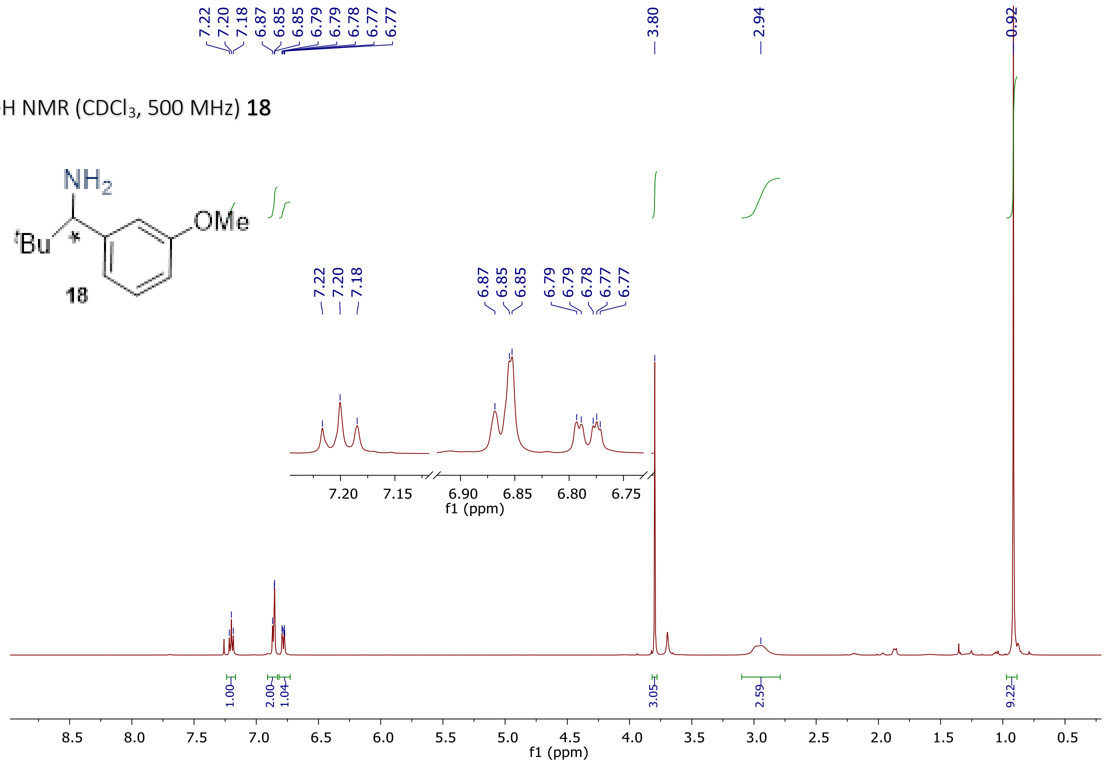


$^{13}$C{$^1$H} NMR ($CDCl_3$, 125 MHz) **18**

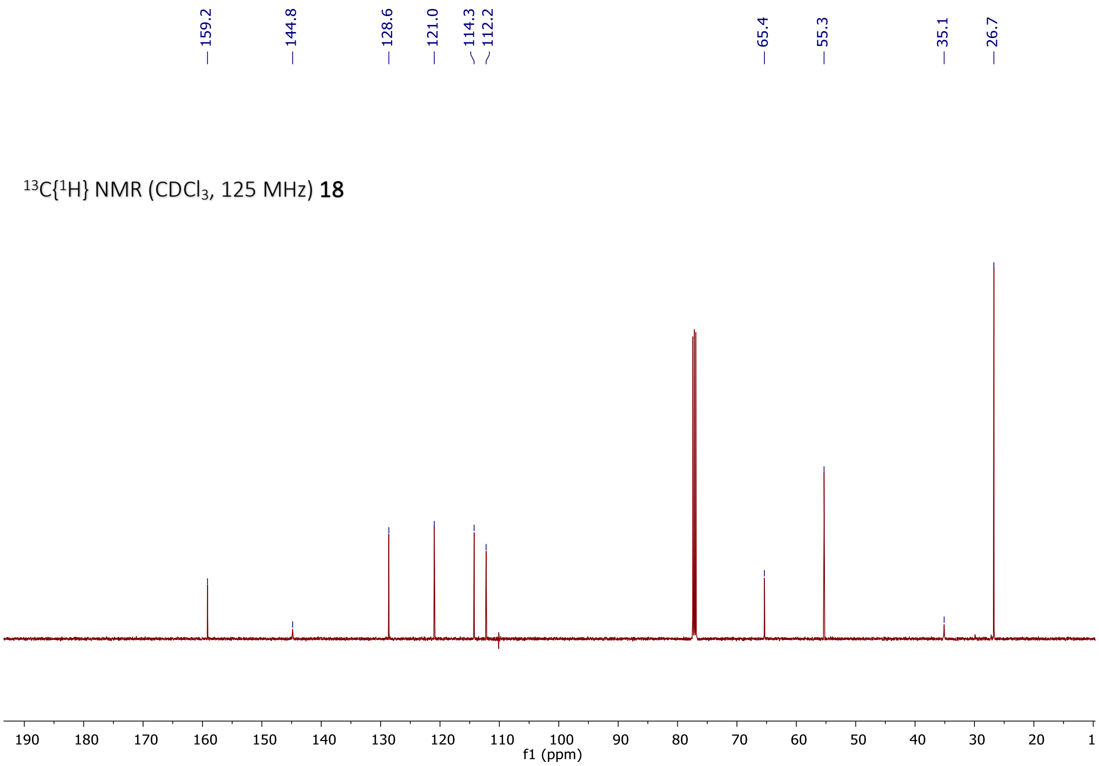

## 2. NMR spectra and HPLC chromatograms for the Grignard additions screening

$^{1}H$ NMR (Table 4, entry 1)

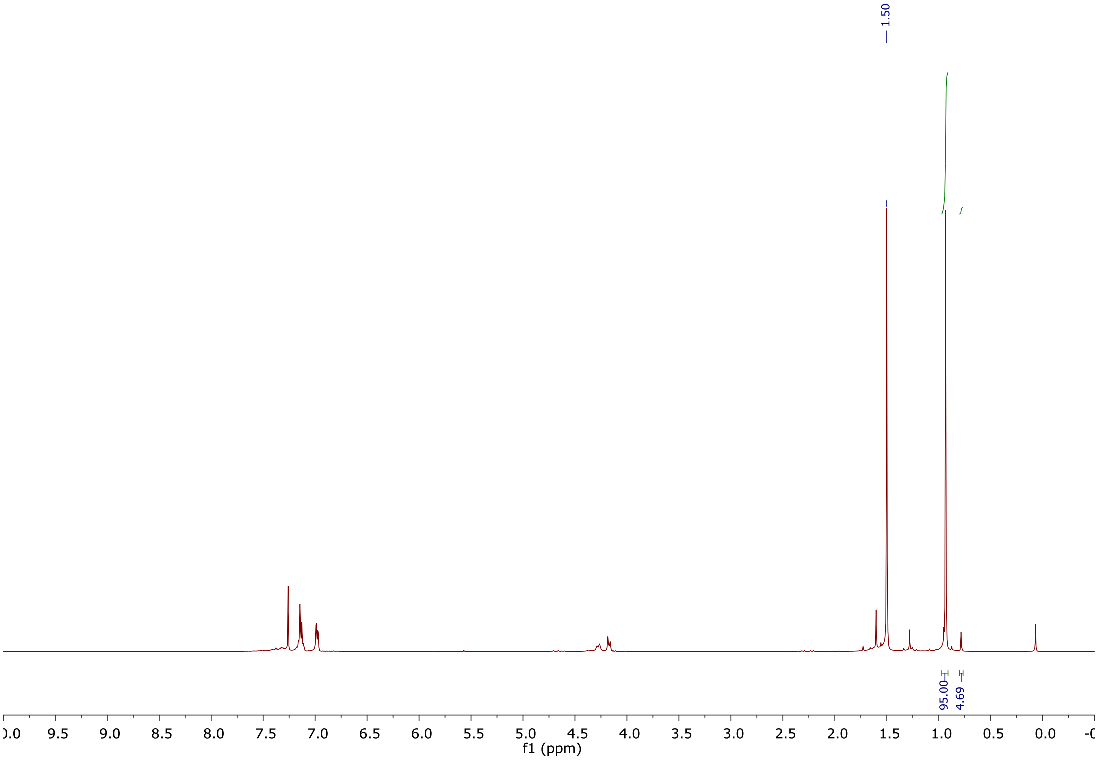


$^{1}H$ NMR (Table 4, entry 2)

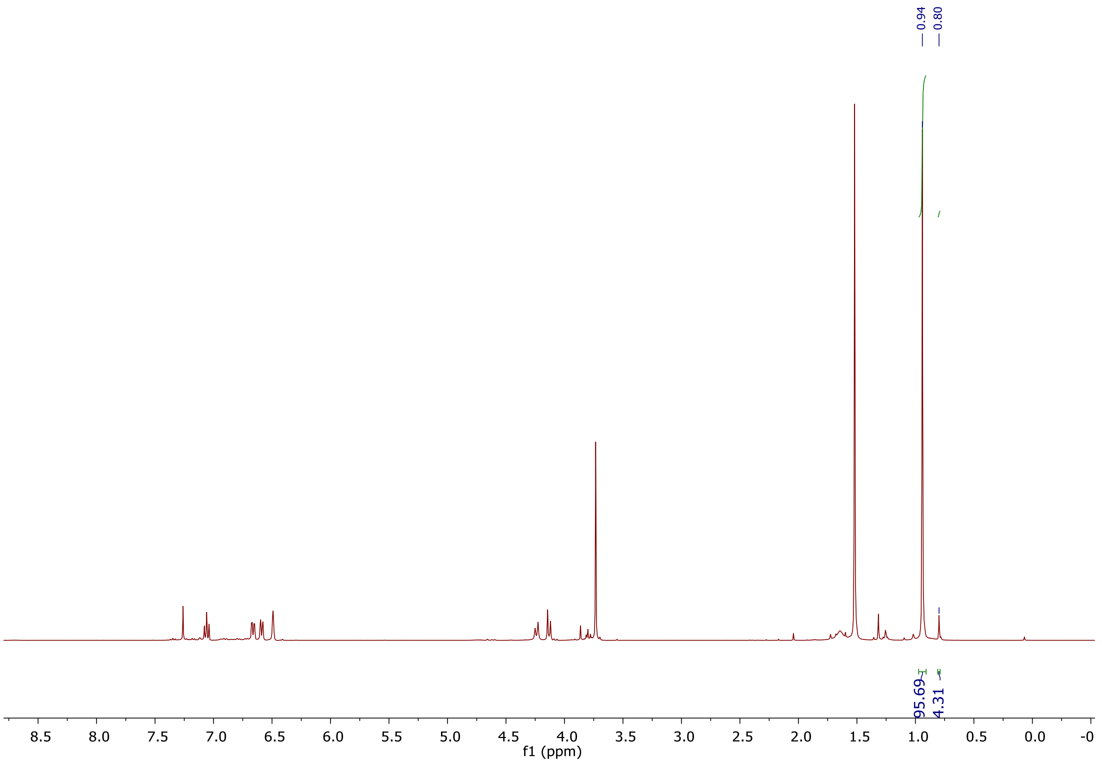

HPLC chromatogram (Table 4, entry 3)

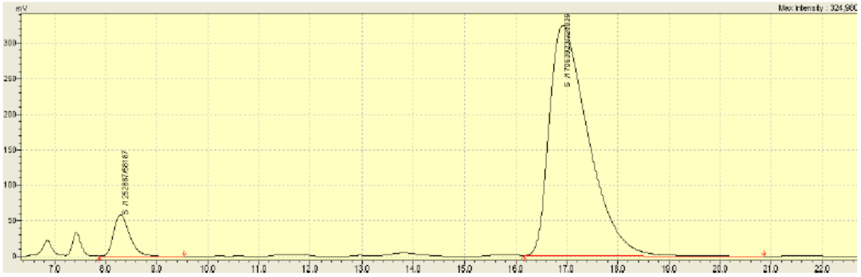

| Ret time[a] | Area | Height | Peak start | Peak end | % Area |
|---|---|---|---|---|---|
| 8.3 | 1252887 | 58187 | 7.875 | 9.542 | 6.8 |
| 16.9 | 17063923 | 323836 | 16.158 | 20.858 | 93.2 |

[a] 0.5% *i*-PrOH

HPLC (Kromasil 5-CelluCoat RP, 0.46 cm × 25 cm, n-hexane/*iso*-propanol = 99.5/0.5, flow rate = 1.0 mL/min, λ = 220 nm) tR = 8.3 min (minor), 16.9 min (major).

$^1$H NMR (Table 4, entry 4)

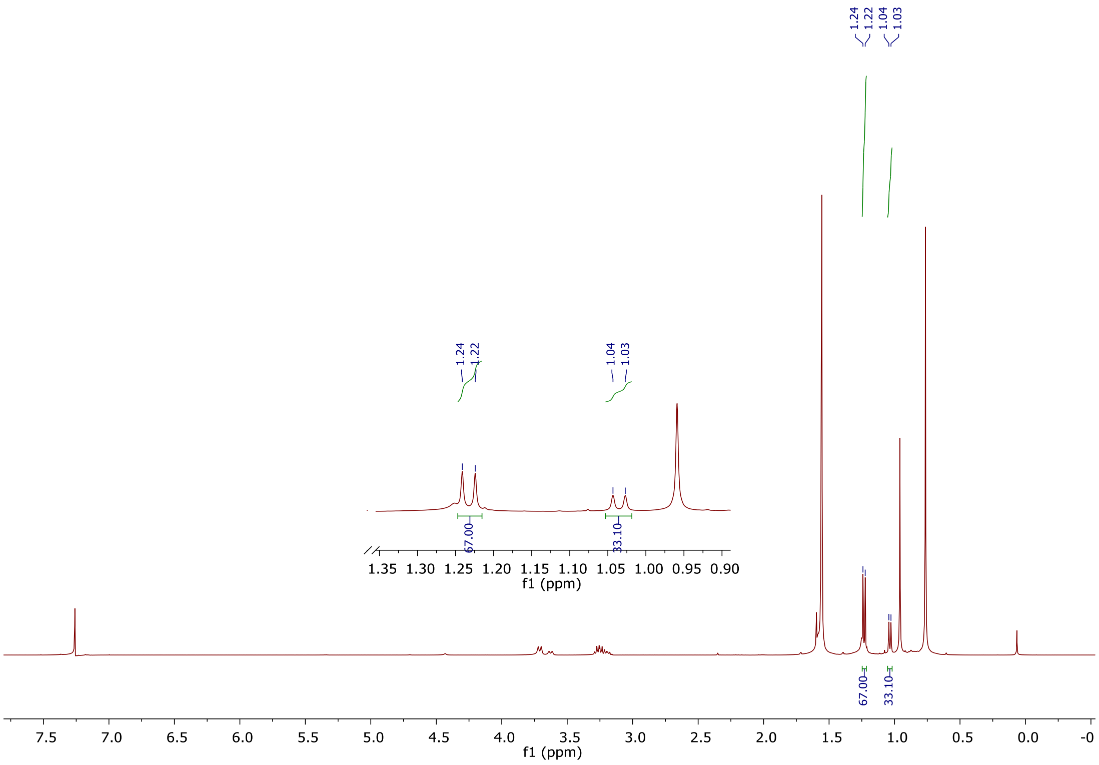

HPLC chromatogram (Table 4, entry 6)

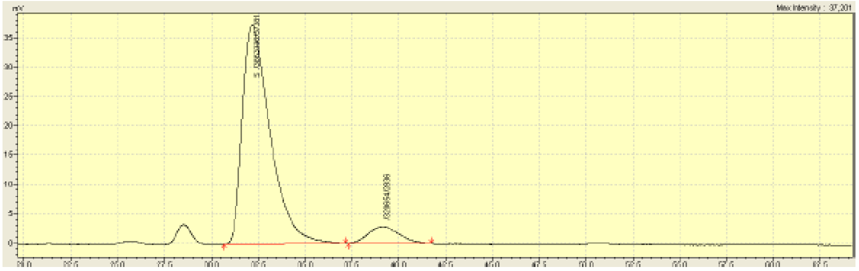

| Ret time[a] | Area | Height | Peak start | Peak end | % Area |
|---|---|---|---|---|---|
| 32.2 | 3863336 | 37381 | 30.692 | 37.15 | 92.3 |
| 39.1 | 320654 | 2836 | 37.292 | 41.733 | 7.7 |

[a] 0.2% *i*-PrOH

HPLC (Kromasil 5-CelluCoat RP, 0.46 cm × 25 cm, n-hexane/*iso*-propanol = 98.8/0.2, flow rate = 1.0 mL/min, λ = 220 nm) tR = 32.2 min (major), 39.1 min (minor).

$^{1}$H NMR (Table 4, entry 7)

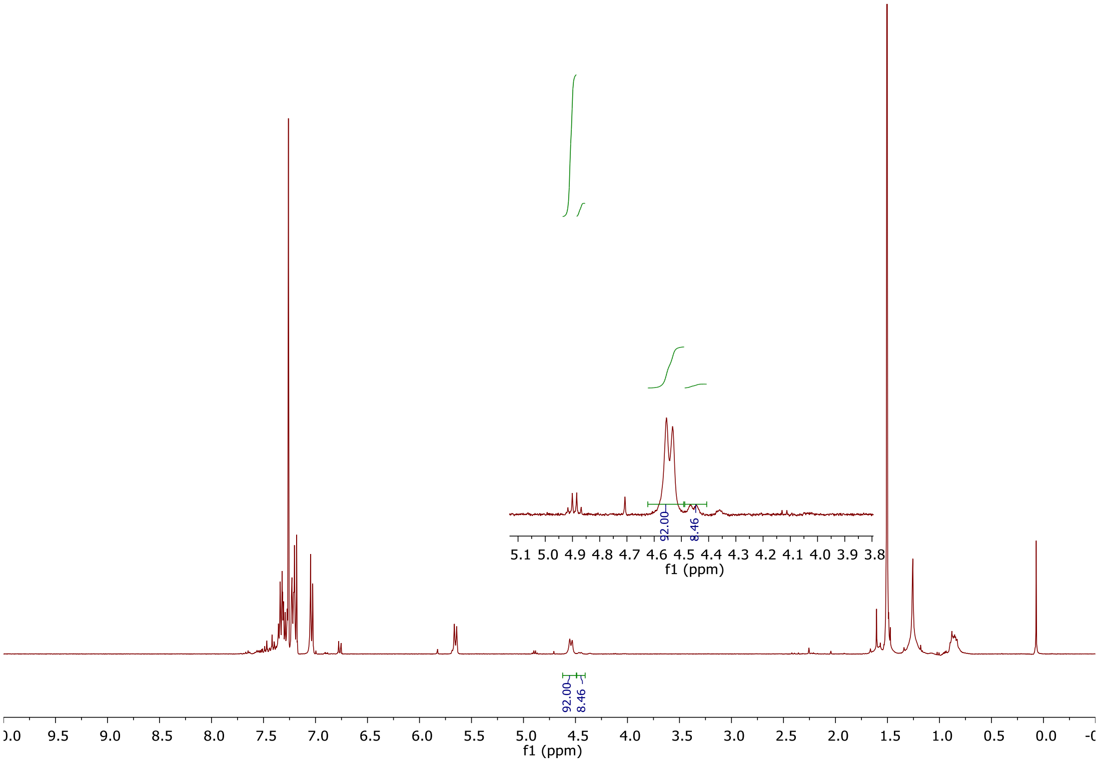

HPLC chromatogram Table 4, entry 8

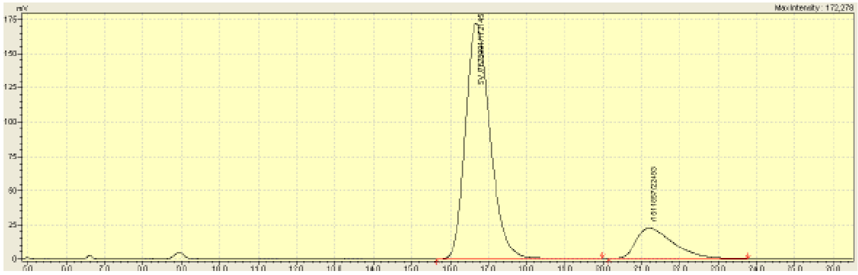

| Ret time[a] | Area | Height | Peak start | Peak end | % Area |
|---|---|---|---|---|---|
| 16.7 | 7678991 | 172145 | 15.667 | 19.975 | 81.0 |
| 21.2 | 1511857 | 22493 | 20.142 | 23.783 | 15.9 |

[a]2% *i*-PrOH

HPLC (Kromasil 5-CelluCoat RP, 0.46 cm × 25 cm, n-hexane/*iso*-propanol = 98/2, flow rate = 1.0 mL/min, λ = 220 nm) tR = 16.7 min (major), 21.2 min (minor).

HPLC chromatogram (Table 4, entry 9)

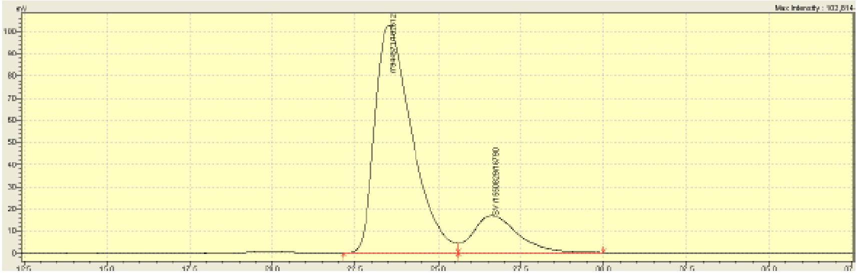

| Ret time[a] | Area | Height | Peak start | Peak end | % Area |
|---|---|---|---|---|---|
| 23.5 | 7944571 | 102612 | 22.15 | 25.608 | 83.7 |
| 26.6 | 1550829 | 16790 | 25.608 | 30 | 16.3 |

[a]0.5% *i*-PrOH

HPLC (Kromasil 5-CelluCoat RP, 0.46 cm × 25 cm, n-hexane/*iso*-propanol = 99.5/0.5, flow rate = 1.0 mL/min, λ = 220 nm) tR = 23.5 min (major), 26.6 min (minor).

$^1$H NMR (Table 4, entry 10)

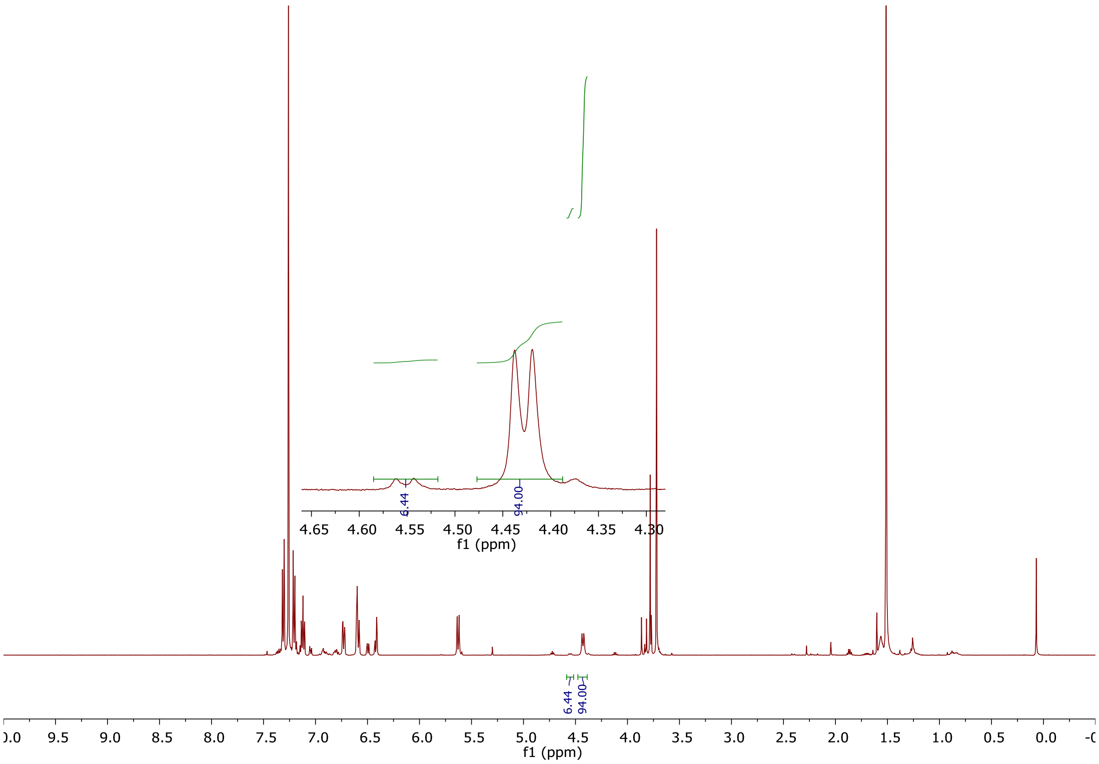

# 3. HRMS spectra

Compound (*S*)-**1**

## Display Report

**Analysis Info**

| | | | |
|---|---|---|---|
| Analysis Name | D:\Data\RawData\Azamat\KTH\Batch_2_final\M1.d | Acquisition Date | 8/22/2021 12:47:18 PM |
| Method | MS_fullscan_positive.m | Operator | Demo User |
| Sample Name | NaFormate | Instrument | impact II 1825265.10235 |
| Comment | | | |

**Acquisition Parameter**

| | | | | | |
|---|---|---|---|---|---|
| Source Type | ESI | Ion Polarity | Positive | Set Nebulizer | 2.0 Bar |
| Focus | Active | Set Capillary | 3500 V | Set Dry Heater | 250 °C |
| Scan Begin | 150 m/z | Set End Plate Offset | -400 V | Set Dry Gas | 8.0 l/min |
| Scan End | 1000 m/z | Set Charging Voltage | 2000 V | Set Divert Valve | Waste |
| | | Set Corona | 4000 nA | Set APCI Heater | 200 °C |

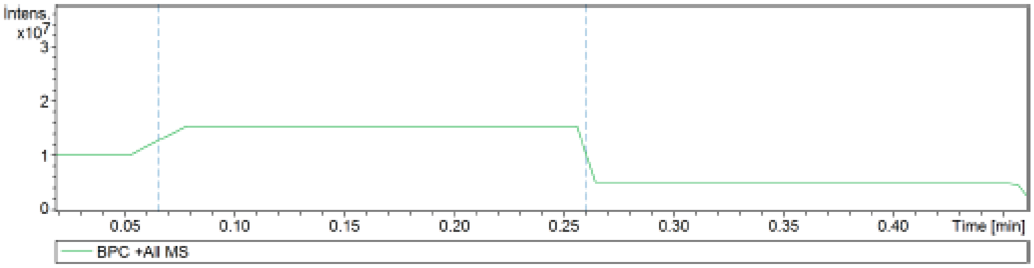


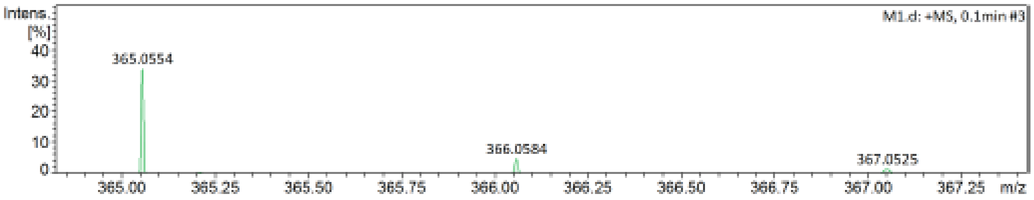


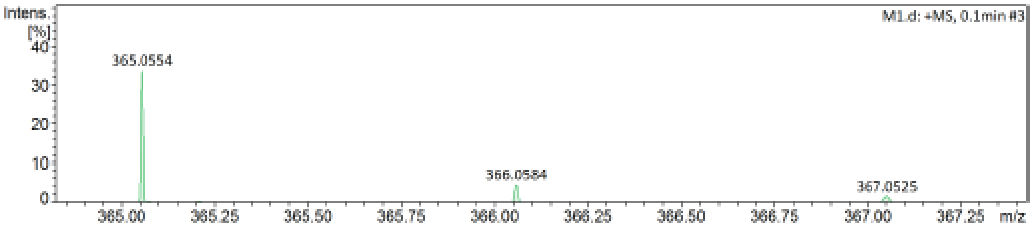


M1.d

Bruker Compass DataAnalysis 4.4 printed: 8/22/2021 5:58:37 PM by: demo Page 1 of 1

Compound (*R*)-**1**

# Display Report

**Analysis Info**

| | | | |
|---|---|---|---|
| Analysis Name | D:\Data\RawData\Azamat\KTH\Batch_2_final\M2.d | Acquisition Date | 8/22/2021 1:06:27 PM |
| Method | MS_fullscan_positive.m | Operator | Demo User |
| Sample Name | NaFormate | Instrument | impact II 1825265.10235 |
| Comment | | | |

**Acquisition Parameter**

| | | | | | |
|---|---|---|---|---|---|
| Source Type | ESI | Ion Polarity | Positive | Set Nebulizer | 2.0 Bar |
| Focus | Active | Set Capillary | 3500 V | Set Dry Heater | 250 °C |
| Scan Begin | 150 m/z | Set End Plate Offset | -400 V | Set Dry Gas | 8.0 l/min |
| Scan End | 1000 m/z | Set Charging Voltage | 2000 V | Set Divert Valve | Waste |
| | | Set Corona | 4000 nA | Set APCI Heater | 200 °C |

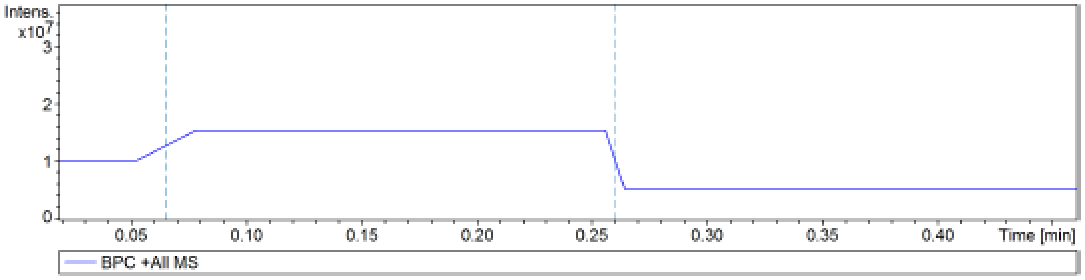


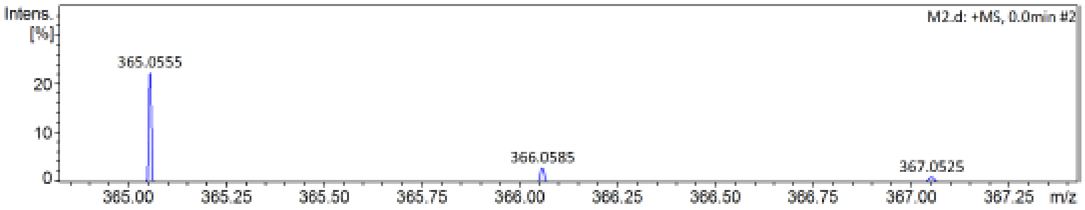


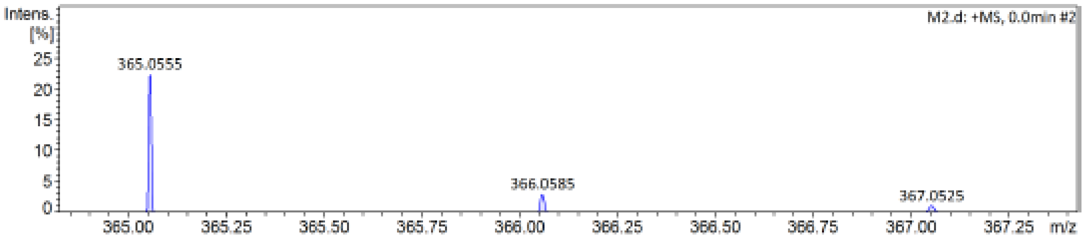


M2.d

Bruker Compass DataAnalysis 4.4 printed: 8/22/2021 6:00:11 PM by: demo Page 1 of 1

Compound (*S*)-**2**

# Display Report

**Analysis Info**

| | | | |
|---|---|---|---|
| Analysis Name | D:\Data\RawData\Azamat\KTH\Batch_2_final\M3.d | Acquisition Date | 8/22/2021 1:08:57 PM |
| Method | MS_fullscan_positive.m | Operator | Demo User |
| Sample Name | NaFormate | Instrument | impact II 1825265.10235 |
| Comment | | | |

**Acquisition Parameter**

| | | | | | |
|---|---|---|---|---|---|
| Source Type | ESI | Ion Polarity | Positive | Set Nebulizer | 2.0 Bar |
| Focus | Active | Set Capillary | 3500 V | Set Dry Heater | 250 °C |
| Scan Begin | 150 m/z | Set End Plate Offset | -400 V | Set Dry Gas | 8.0 l/min |
| Scan End | 1000 m/z | Set Charging Voltage | 2000 V | Set Divert Valve | Waste |
| | | Set Corona | 4000 nA | Set APCI Heater | 200 °C |

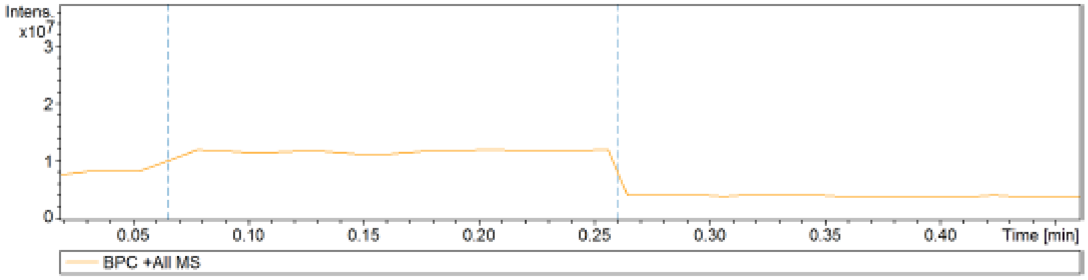


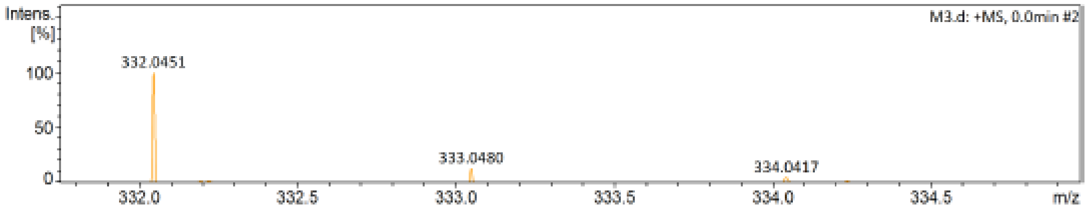


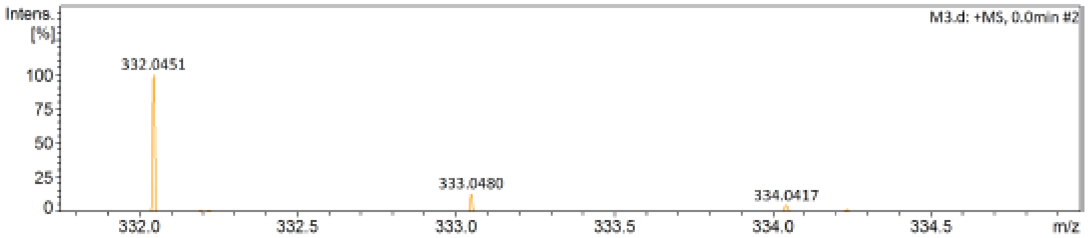


M3.d

Bruker Compass DataAnalysis 4.4 printed: 8/22/2021 6:01:46 PM by: demo Page 1 of 1

Compound (*S*)-**3**

# Display Report

**Analysis Info**

| | | | |
|---|---|---|---|
| Analysis Name | D:\Data\RawData\Azamat\KTH\Batch_2_final\M4.d | Acquisition Date | 8/22/2021 12:00:36 PM |
| Method | MS_fullscan_positive.m | Operator | Demo User |
| Sample Name | NaFormate | Instrument | impact II 1825265.10235 |
| Comment | | | |

**Acquisition Parameter**

| | | | | | |
|---|---|---|---|---|---|
| Source Type | ESI | Ion Polarity | Positive | Set Nebulizer | 2.0 Bar |
| Focus | Active | Set Capillary | 3500 V | Set Dry Heater | 250 °C |
| Scan Begin | 150 m/z | Set End Plate Offset | -400 V | Set Dry Gas | 8.0 l/min |
| Scan End | 1000 m/z | Set Charging Voltage | 2000 V | Set Divert Valve | Waste |
| | | Set Corona | 4000 nA | Set APCI Heater | 200 °C |

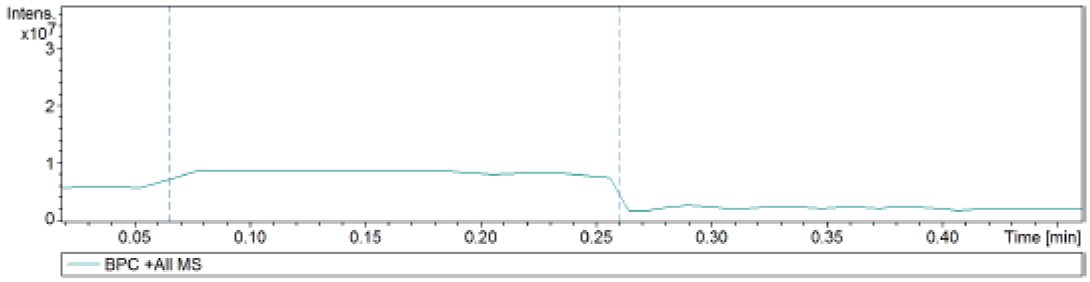


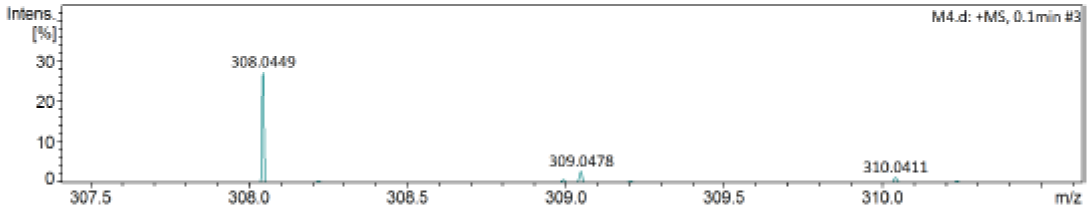


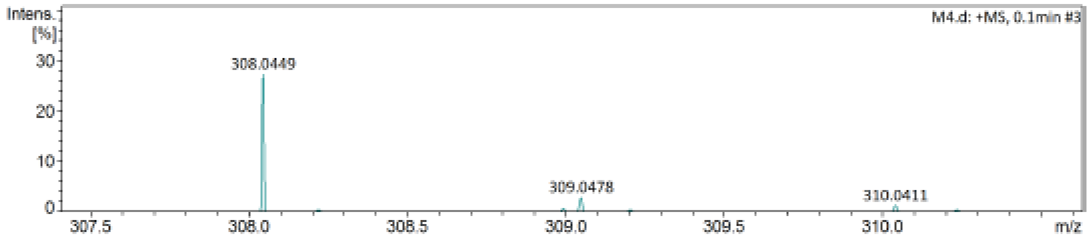

Compound (*R*)-**3**

# Display Report

**Analysis Info**

| | | | |
|---|---|---|---|
| Analysis Name | D:\Data\RawData\Azamat\KTH\Batch_2_final\M5.d | Acquisition Date | 8/22/2021 12:41:50 PM |
| Method | MS_fullscan_positive.m | Operator | Demo User |
| Sample Name | NaFormate | Instrument | impact II 1825265.10235 |
| Comment | | | |

**Acquisition Parameter**

| | | | | | |
|---|---|---|---|---|---|
| Source Type | ESI | Ion Polarity | Positive | Set Nebulizer | 2.0 Bar |
| Focus | Active | Set Capillary | 3500 V | Set Dry Heater | 250 °C |
| Scan Begin | 150 m/z | Set End Plate Offset | -400 V | Set Dry Gas | 8.0 l/min |
| Scan End | 1000 m/z | Set Charging Voltage | 2000 V | Set Divert Valve | Waste |
| | | Set Corona | 4000 nA | Set APCI Heater | 200 °C |

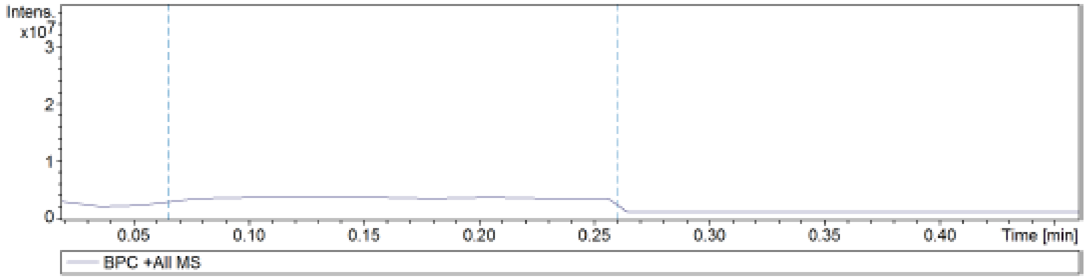


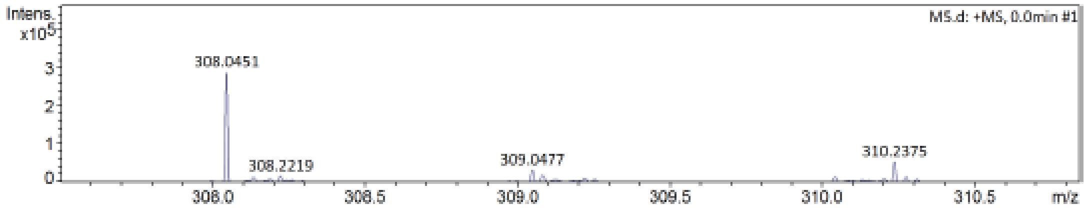


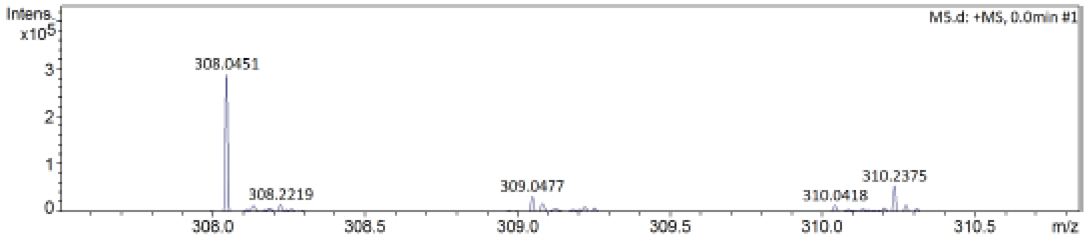


M5.d

Bruker Compass DataAnalysis 4.4 printed: 8/22/2021 6:06:18 PM by: demo Page 1 of 1

Compound (*S*)-**4**

# Display Report

**Analysis Info**

| | | | |
|---|---|---|---|
| Analysis Name | D:\Data\RawData\Azamat\KTH\Batch_2_final\M6.d | Acquisition Date | 8/22/2021 12:34:27 PM |
| Method | MS_fullscan_positive.m | Operator | Demo User |
| Sample Name | NaFormate | Instrument | impact II 1825265.10235 |
| Comment | | | |

**Acquisition Parameter**

| | | | | | |
|---|---|---|---|---|---|
| Source Type | ESI | Ion Polarity | Positive | Set Nebulizer | 2.0 Bar |
| Focus | Active | Set Capillary | 3500 V | Set Dry Heater | 250 °C |
| Scan Begin | 150 m/z | Set End Plate Offset | -400 V | Set Dry Gas | 8.0 l/min |
| Scan End | 1000 m/z | Set Charging Voltage | 2000 V | Set Divert Valve | Waste |
| | | Set Corona | 4000 nA | Set APCI Heater | 200 °C |

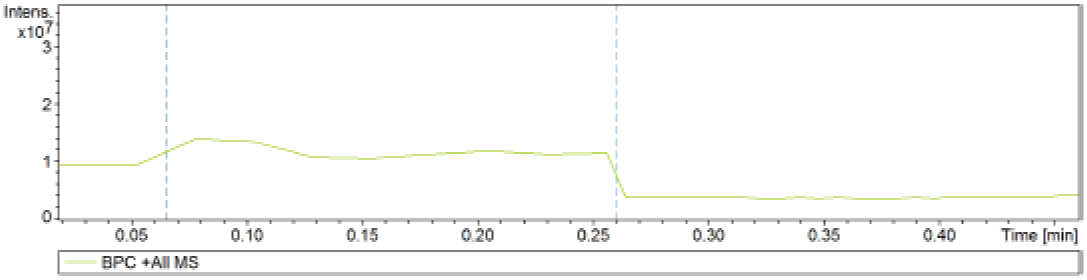


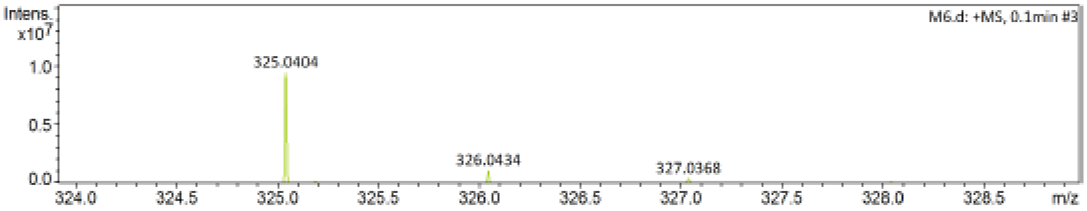


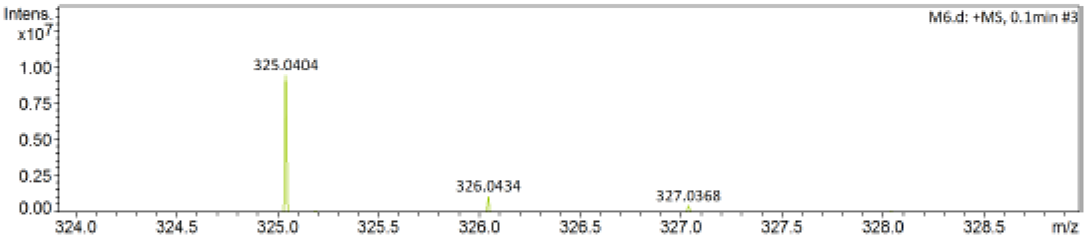


M6.d

Bruker Compass DataAnalysis 4.4 printed: 8/22/2021 6:08:16 PM by: demo Page 1 of 1

Compound (*S*)-**5**

## Display Report

**Analysis Info**

| | | | |
|---|---|---|---|
| Analysis Name | D:\Data\RawData\Azamat\KTH\Batch_2_final\M7.d | Acquisition Date | 8/22/2021 12:11:18 PM |
| Method | MS_fullscan_positive.m | Operator | Demo User |
| Sample Name | NaFormate | Instrument | impact II 1825265.10235 |
| Comment | | | |

**Acquisition Parameter**

| | | | | | |
|---|---|---|---|---|---|
| Source Type | ESI | Ion Polarity | Positive | Set Nebulizer | 2.0 Bar |
| Focus | Active | Set Capillary | 3500 V | Set Dry Heater | 250 °C |
| Scan Begin | 150 m/z | Set End Plate Offset | -400 V | Set Dry Gas | 8.0 l/min |
| Scan End | 1000 m/z | Set Charging Voltage | 2000 V | Set Divert Valve | Waste |
| | | Set Corona | 4000 nA | Set APCI Heater | 200 °C |

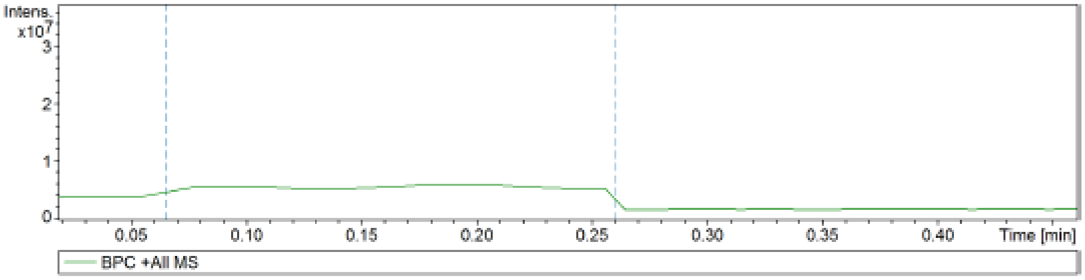


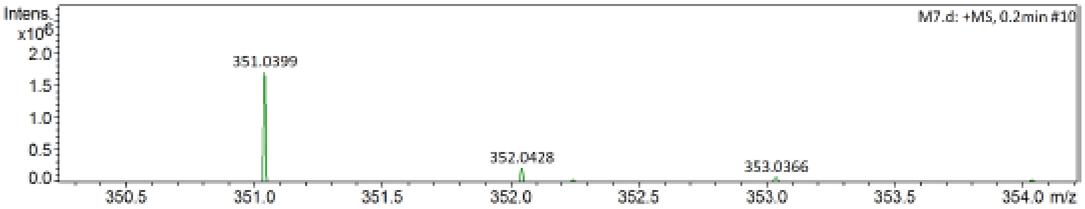


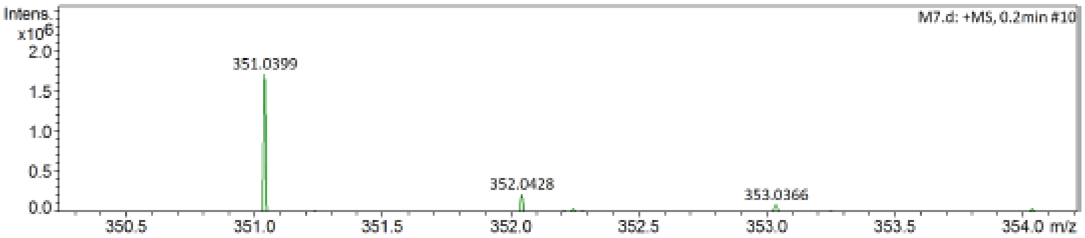


M7.d

Compound (*S*)-**7**

# Display Report

**Analysis Info**

| | | | |
|---|---|---|---|
| Analysis Name | D:\Data\RawData\Azamat\KTH\Batch_2_final\M8.d | Acquisition Date | 8/22/2021 12:31:53 PM |
| Method | MS_fullscan_positive.m | Operator | Demo User |
| Sample Name | NaFormate | Instrument | impact II 1825265.10235 |
| Comment | | | |

**Acquisition Parameter**

| | | | | | |
|---|---|---|---|---|---|
| Source Type | ESI | Ion Polarity | Positive | Set Nebulizer | 2.0 Bar |
| Focus | Active | Set Capillary | 3500 V | Set Dry Heater | 250 °C |
| Scan Begin | 150 m/z | Set End Plate Offset | -400 V | Set Dry Gas | 8.0 l/min |
| Scan End | 1000 m/z | Set Charging Voltage | 2000 V | Set Divert Valve | Waste |
| | | Set Corona | 4000 nA | Set APCI Heater | 200 °C |

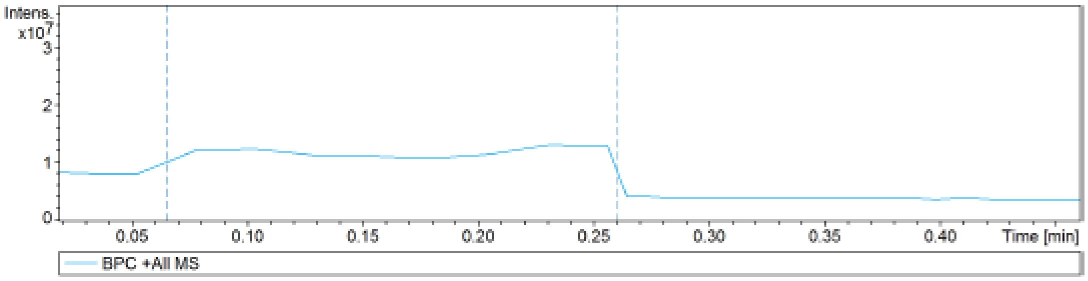


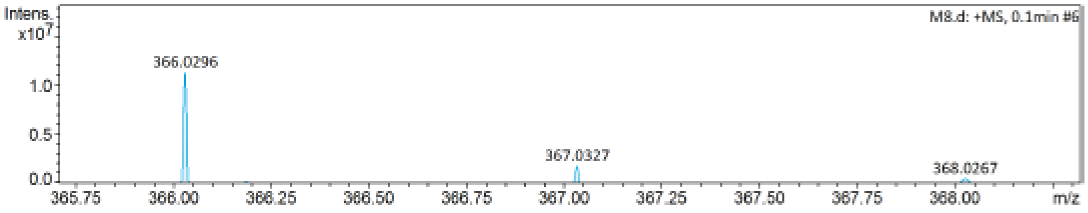


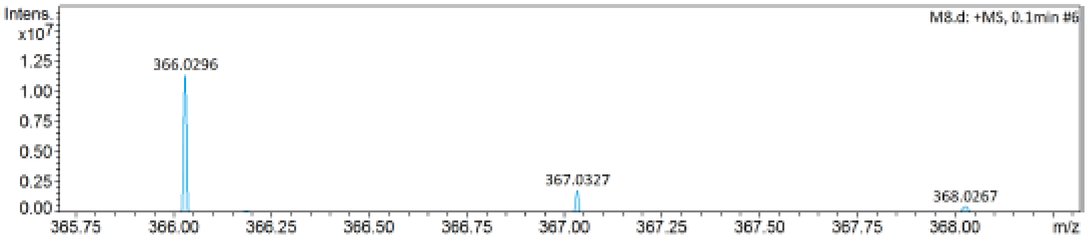

Compound Rac-**8**

# Display Report

**Analysis Info**

| | | | |
|---|---|---|---|
| Analysis Name | D:\Data\RawData\Azamat\KTH\Batch_2_final\M10.d | Acquisition Date | 8/22/2021 12:08:39 PM |
| Method | MS_fullscan_positive.m | Operator | Demo User |
| Sample Name | NaFormate | Instrument | impact II 1825265.10235 |
| Comment | | | |

**Acquisition Parameter**

| | | | | | |
|---|---|---|---|---|---|
| Source Type | ESI | Ion Polarity | Positive | Set Nebulizer | 2.0 Bar |
| Focus | Active | Set Capillary | 3500 V | Set Dry Heater | 250 °C |
| Scan Begin | 150 m/z | Set End Plate Offset | -400 V | Set Dry Gas | 8.0 l/min |
| Scan End | 1000 m/z | Set Charging Voltage | 2000 V | Set Divert Valve | Waste |
| | | Set Corona | 4000 nA | Set APCI Heater | 200 °C |

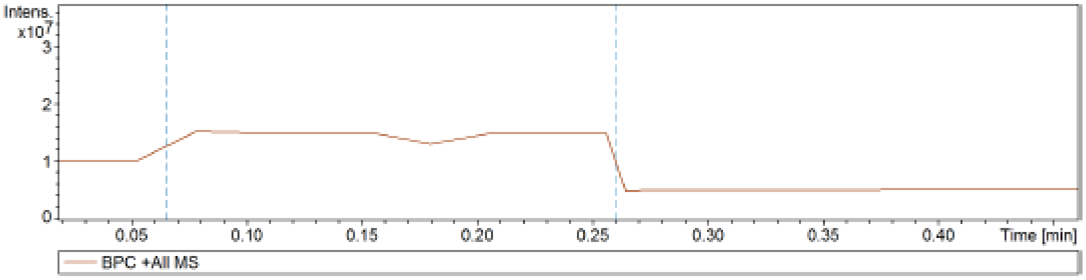


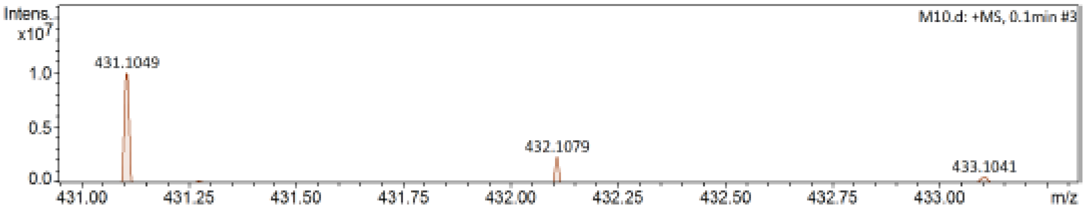


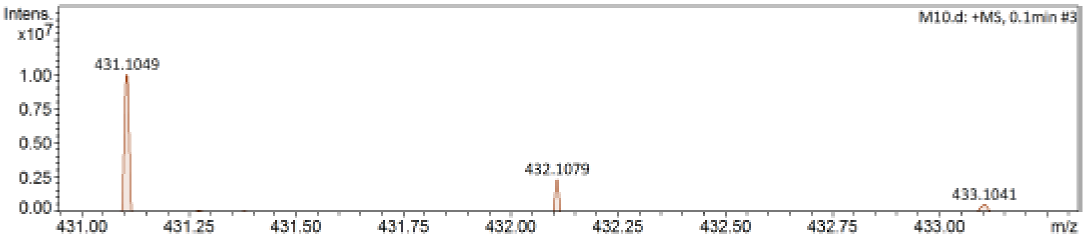


M10.d

Bruker Compass DataAnalysis 4.4 printed: 8/22/2021 6:13:11 PM by: demo Page 1 of 1

Compound **9**

# Display Report

**Analysis Info**

| | | | |
|---|---|---|---|
| Analysis Name | D:\Data\RawData\Azamat\KTH\Batch_2_final\M11.d | Acquisition Date | 8/22/2021 12:54:55 PM |
| Method | MS_fullscan_positive.m | Operator | Demo User |
| Sample Name | NaFormate | Instrument | impact II 1825265.10235 |
| Comment | | | |

**Acquisition Parameter**

| | | | | | |
|---|---|---|---|---|---|
| Source Type | ESI | Ion Polarity | Positive | Set Nebulizer | 2.0 Bar |
| Focus | Active | Set Capillary | 3500 V | Set Dry Heater | 250 °C |
| Scan Begin | 150 m/z | Set End Plate Offset | -400 V | Set Dry Gas | 8.0 l/min |
| Scan End | 1000 m/z | Set Charging Voltage | 2000 V | Set Divert Valve | Waste |
| | | Set Corona | 4000 nA | Set APCI Heater | 200 °C |

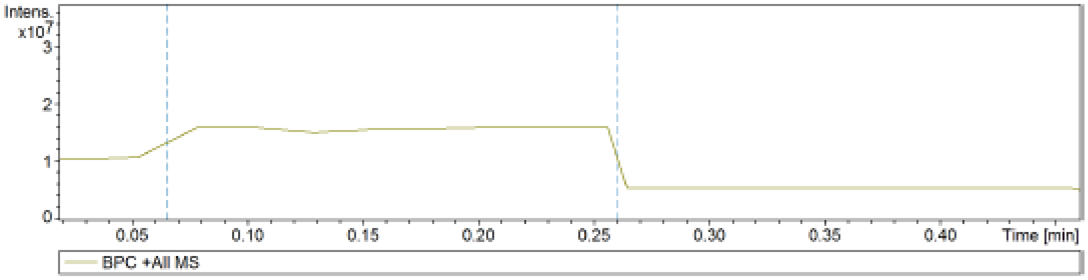


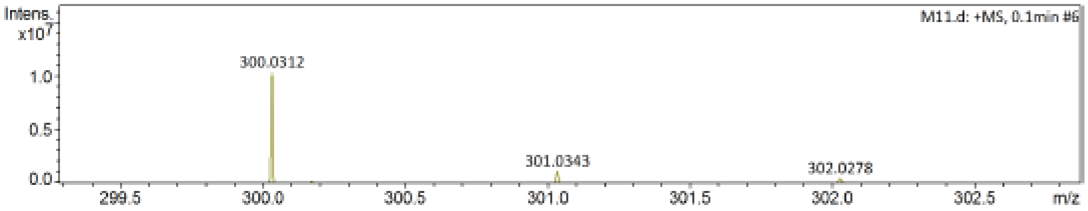


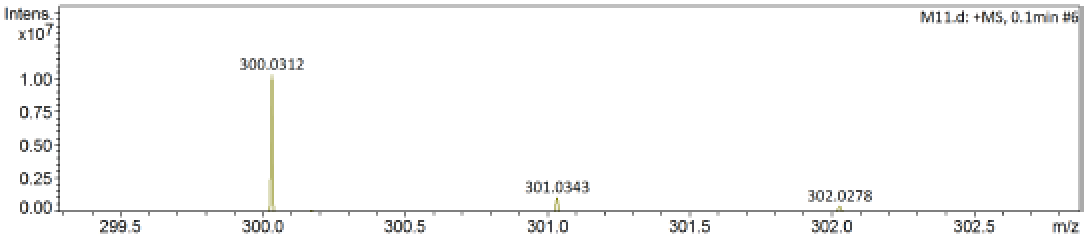


M11.d

Bruker Compass DataAnalysis 4.4 printed: 8/22/2021 6:15:46 PM by: demo Page 1 of 1

Compound Rac-**10**

# Display Report

**Analysis Info**

| | | | |
|---|---|---|---|
| Analysis Name | D:\Data\RawData\Azamat\KTH\Batch_2_final\M12.d | Acquisition Date | 8/22/2021 12:03:09 PM |
| Method | MS_fullscan_positive.m | Operator | Demo User |
| Sample Name | NaFormate | Instrument | impact II 1825265.10235 |
| Comment | | | |

**Acquisition Parameter**

| | | | | | |
|---|---|---|---|---|---|
| Source Type | ESI | Ion Polarity | Positive | Set Nebulizer | 2.0 Bar |
| Focus | Active | Set Capillary | 3500 V | Set Dry Heater | 250 °C |
| Scan Begin | 150 m/z | Set End Plate Offset | -400 V | Set Dry Gas | 8.0 l/min |
| Scan End | 1000 m/z | Set Charging Voltage | 2000 V | Set Divert Valve | Waste |
| | | Set Corona | 4000 nA | Set APCI Heater | 200 °C |

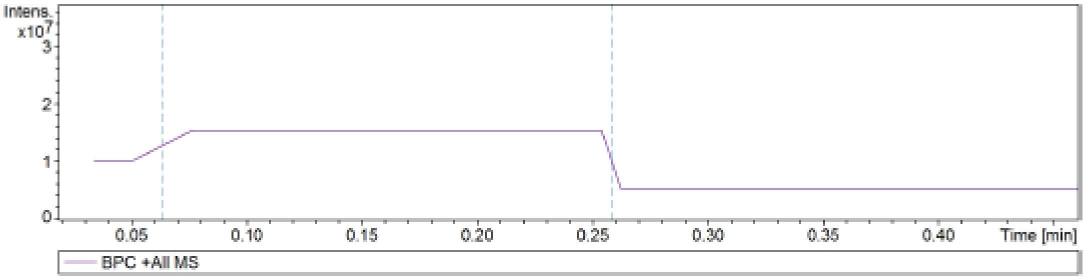


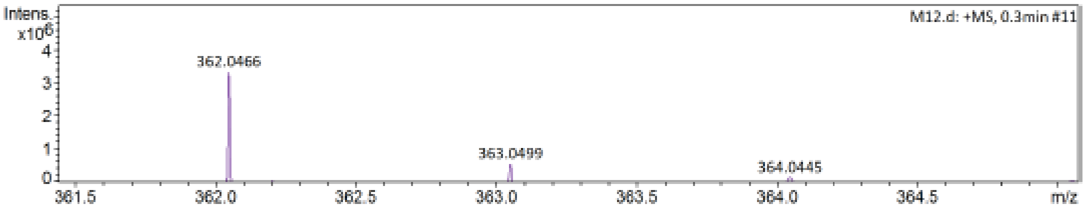


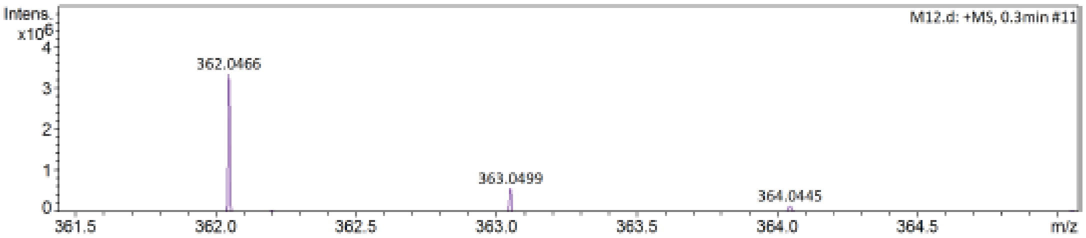

Rac-**11**

# Display Report

**Analysis Info**

| | | | |
|---|---|---|---|
| Analysis Name | D:\Data\RawData\Azamat\KTH\Batch_2_final\M13.d | Acquisition Date | 8/22/2021 12:37:33 PM |
| Method | MS_fullscan_positive.m | Operator | Demo User |
| Sample Name | NaFormate | Instrument | impact II 1825265.10235 |
| Comment | | | |

**Acquisition Parameter**

| | | | | | |
|---|---|---|---|---|---|
| Source Type | ESI | Ion Polarity | Positive | Set Nebulizer | 2.0 Bar |
| Focus | Active | Set Capillary | 3500 V | Set Dry Heater | 250 °C |
| Scan Begin | 150 m/z | Set End Plate Offset | -400 V | Set Dry Gas | 8.0 l/min |
| Scan End | 1000 m/z | Set Charging Voltage | 2000 V | Set Divert Valve | Waste |
| | | Set Corona | 4000 nA | Set APCI Heater | 200 °C |

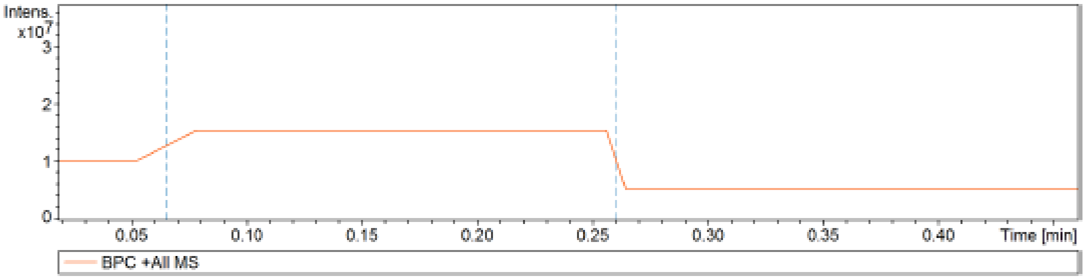


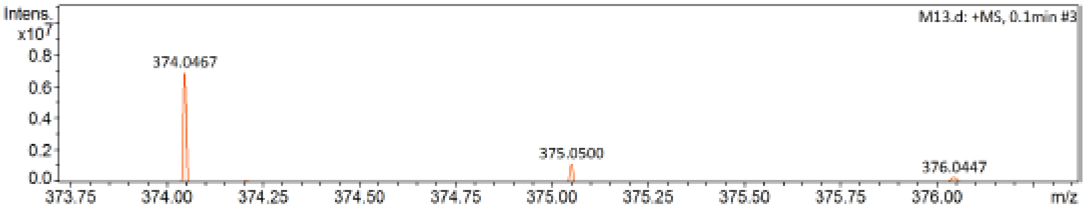


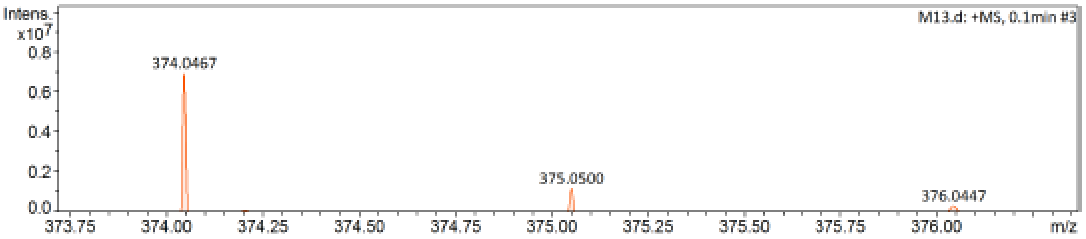

Compound Rac-**12**

# Display Report

**Analysis Info**

| | | | |
|---|---|---|---|
| Analysis Name | D:\Data\RawData\Azamat\KTH\Batch_2_final\M14.d | Acquisition Date | 8/22/2021 1:03:44 PM |
| Method | MS_fullscan_positive.m | Operator | Demo User |
| Sample Name | NaFormate | Instrument | impact II 1825265.10235 |
| Comment | | | |

**Acquisition Parameter**

| | | | | | |
|---|---|---|---|---|---|
| Source Type | ESI | Ion Polarity | Positive | Set Nebulizer | 2.0 Bar |
| Focus | Active | Set Capillary | 3500 V | Set Dry Heater | 250 °C |
| Scan Begin | 150 m/z | Set End Plate Offset | -400 V | Set Dry Gas | 8.0 l/min |
| Scan End | 1000 m/z | Set Charging Voltage | 2000 V | Set Divert Valve | Waste |
| | | Set Corona | 4000 nA | Set APCI Heater | 200 °C |

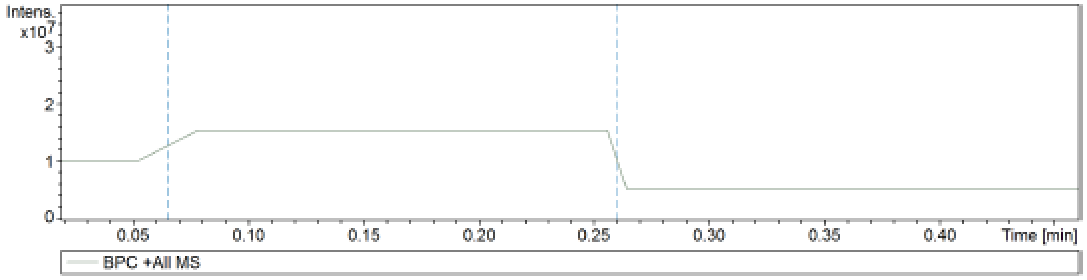


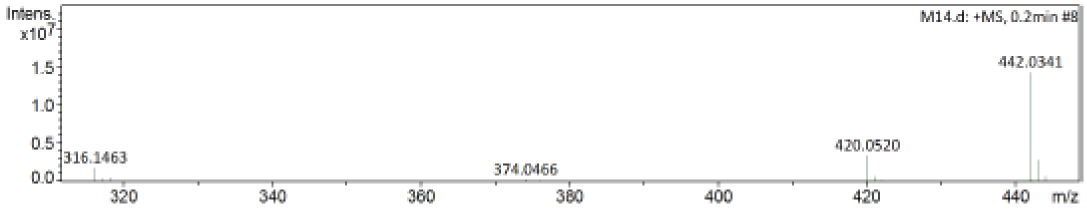


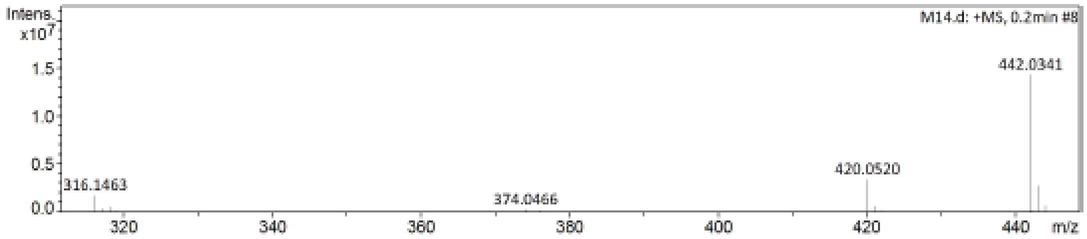


M14.d

Bruker Compass DataAnalysis 4.4 printed: 8/22/2021 6:27:33 PM by: demo Page 1 of 1

Compound (*R*)-**13**

# Display Report

**Analysis Info**

| | | | |
|---|---|---|---|
| Analysis Name | D:\Data\RawData\Azamat\KTH\Batch_2_final\M15.d | Acquisition Date | 8/22/2021 12:57:23 PM |
| Method | MS_fullscan_positive.m | Operator | Demo User |
| Sample Name | NaFormate | Instrument | impact II 1825265.10235 |
| Comment | | | |

**Acquisition Parameter**

| | | | | | |
|---|---|---|---|---|---|
| Source Type | ESI | Ion Polarity | Positive | Set Nebulizer | 2.0 Bar |
| Focus | Active | Set Capillary | 3500 V | Set Dry Heater | 250 °C |
| Scan Begin | 150 m/z | Set End Plate Offset | -400 V | Set Dry Gas | 8.0 l/min |
| Scan End | 1000 m/z | Set Charging Voltage | 2000 V | Set Divert Valve | Waste |
| | | Set Corona | 4000 nA | Set APCI Heater | 200 °C |

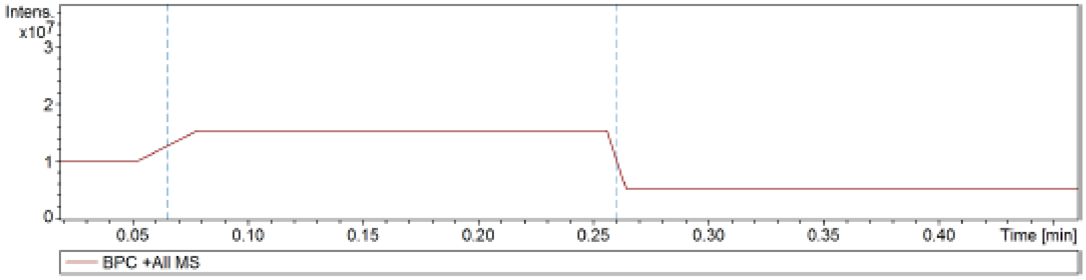


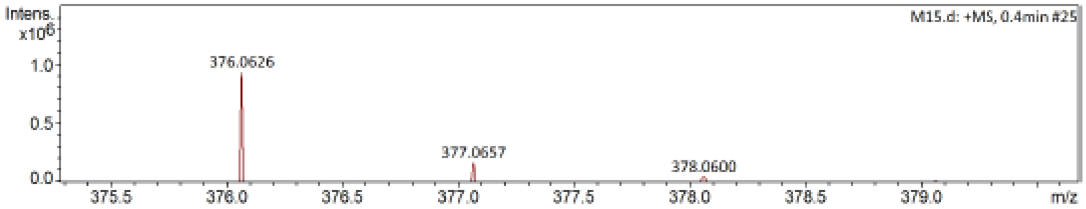


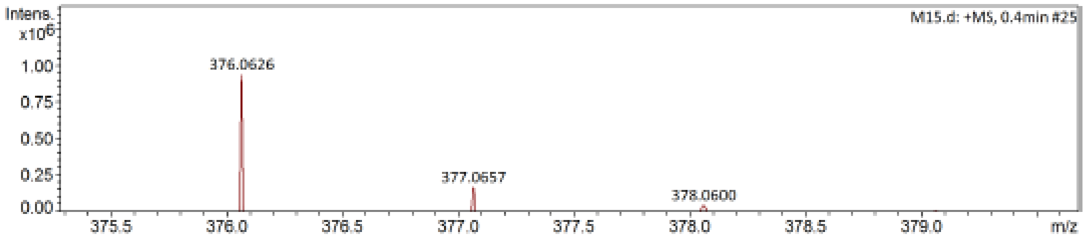


M15.d

Bruker Compass DataAnalysis 4.4 printed: 8/22/2021 6:29:29 PM by: demo Page 1 of 1

Compound rac-**14**

# Display Report

**Analysis Info**

| | | | |
|---|---|---|---|
| Analysis Name | D:\Data\RawData\Azamat\KTH\Batch_2_final\M16.d | Acquisition Date | 8/22/2021 12:13:59 PM |
| Method | MS_fullscan_positive.m | Operator | Demo User |
| Sample Name | NaFormate | Instrument | impact II 1825265.10235 |
| Comment | | | |

**Acquisition Parameter**

| | | | | | |
|---|---|---|---|---|---|
| Source Type | ESI | Ion Polarity | Positive | Set Nebulizer | 2.0 Bar |
| Focus | Active | Set Capillary | 3500 V | Set Dry Heater | 250 °C |
| Scan Begin | 150 m/z | Set End Plate Offset | -400 V | Set Dry Gas | 8.0 l/min |
| Scan End | 1000 m/z | Set Charging Voltage | 2000 V | Set Divert Valve | Waste |
| | | Set Corona | 4000 nA | Set APCI Heater | 200 °C |

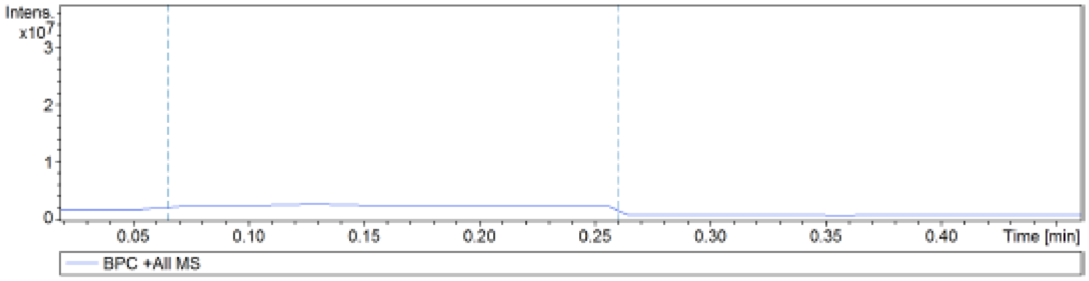


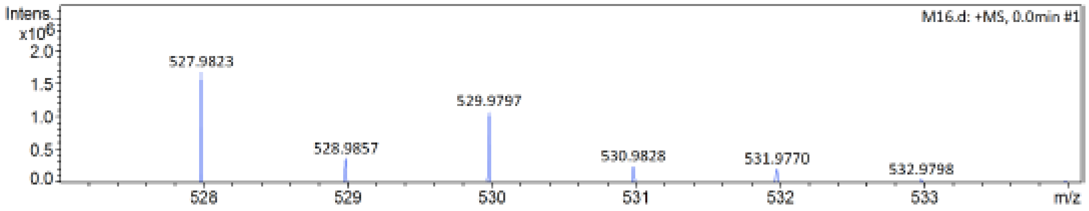


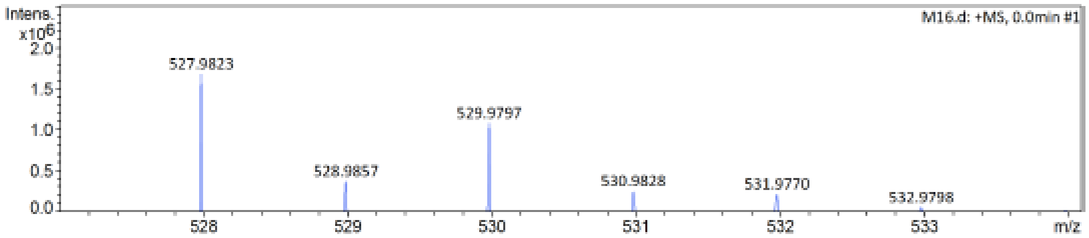

Compound **15**

# Display Report

**Analysis Info**

| | | | |
|---|---|---|---|
| Analysis Name | D:\Data\RawData\Azamat\KTH\Batch_2_final\M17.d | Acquisition Date | 8/22/2021 1:11:36 PM |
| Method | MS_fullscan_positive.m | Operator | Demo User |
| Sample Name | NaFormate | Instrument | impact II 1825265.10235 |
| Comment | | | |

**Acquisition Parameter**

| | | | | | |
|---|---|---|---|---|---|
| Source Type | ESI | Ion Polarity | Positive | Set Nebulizer | 2.0 Bar |
| Focus | Active | Set Capillary | 3500 V | Set Dry Heater | 250 °C |
| Scan Begin | 150 m/z | Set End Plate Offset | -400 V | Set Dry Gas | 8.0 l/min |
| Scan End | 1000 m/z | Set Charging Voltage | 2000 V | Set Divert Valve | Waste |
| | | Set Corona | 4000 nA | Set APCI Heater | 200 °C |

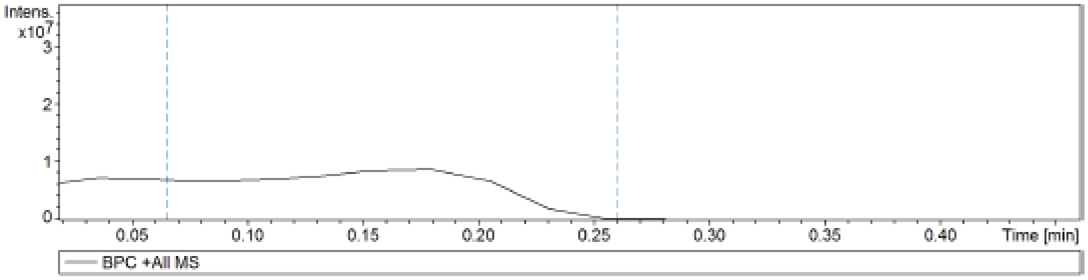


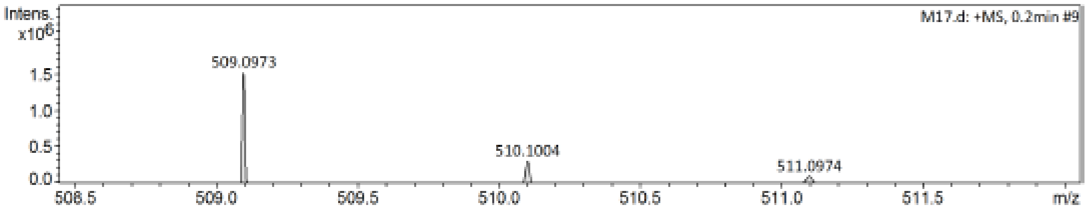


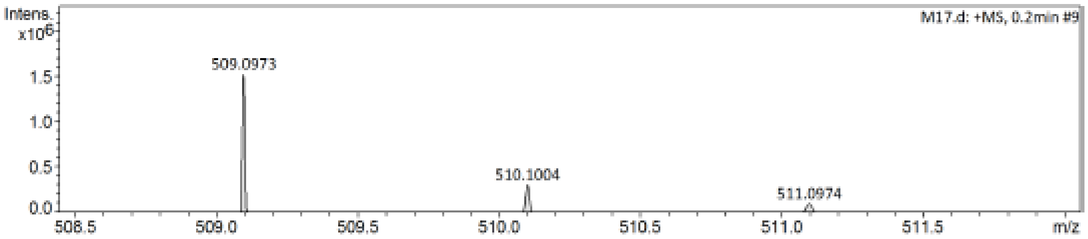


M17.d

Bruker Compass DataAnalysis 4.4 printed: 8/22/2021 6:31:54 PM by: demo Page 1 of 1

Compound (*R*)-**16a**

# Display Report

**Analysis Info**

| | | | |
|---|---|---|---|
| Analysis Name | D:\Data\RawData\Azamat\KTH\Batch_2_final\M18.d | Acquisition Date | 8/22/2021 1:13:21 PM |
| Method | MS_fullscan_positive.m | Operator | Demo User |
| Sample Name | NaFormate | Instrument | impact II 1825265.10235 |
| Comment | | | |

**Acquisition Parameter**

| | | | | | |
|---|---|---|---|---|---|
| Source Type | ESI | Ion Polarity | Positive | Set Nebulizer | 2.0 Bar |
| Focus | Active | Set Capillary | 3500 V | Set Dry Heater | 250 °C |
| Scan Begin | 150 m/z | Set End Plate Offset | -400 V | Set Dry Gas | 8.0 l/min |
| Scan End | 1000 m/z | Set Charging Voltage | 2000 V | Set Divert Valve | Waste |
| | | Set Corona | 4000 nA | Set APCI Heater | 200 °C |

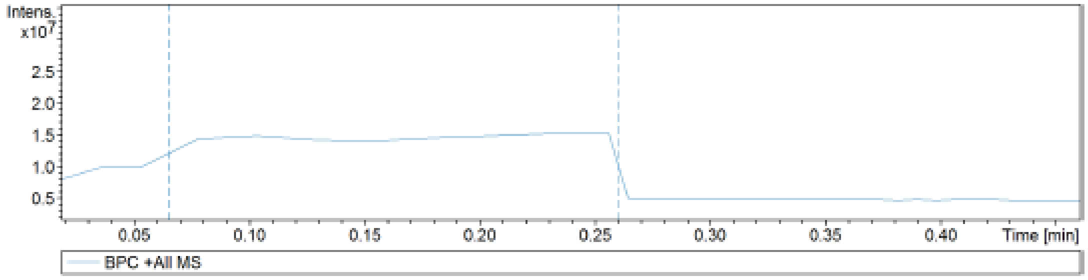


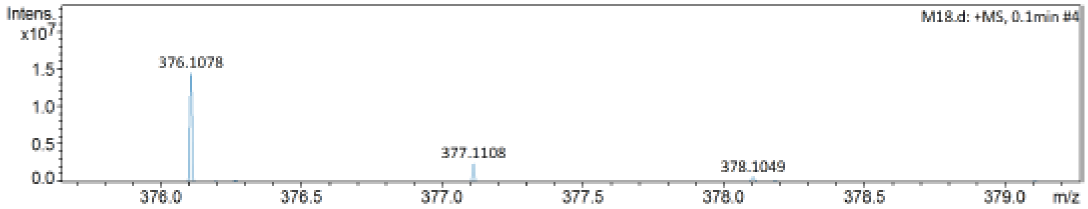


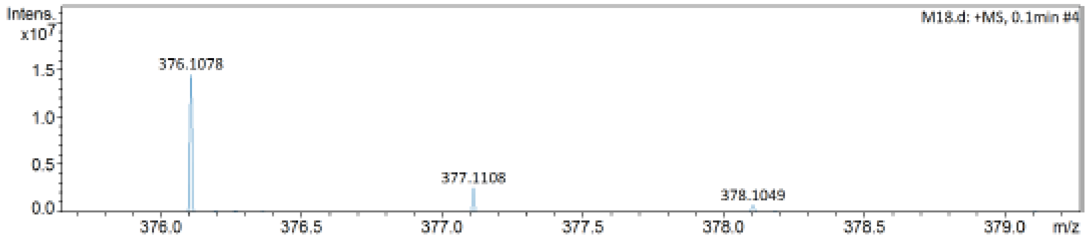

Compound (*R*)-**16b**

# Display Report

**Analysis Info**

| | | | |
|---|---|---|---|
| Analysis Name | D:\Data\RawData\Azamat\KTH\Batch_2_final\M1a.d | Acquisition Date | 8/22/2021 1:15:54 PM |
| Method | MS_fullscan_positive.m | Operator | Demo User |
| Sample Name | NaFormate | Instrument | impact II 1825265.10235 |
| Comment | | | |

**Acquisition Parameter**

| | | | | | |
|---|---|---|---|---|---|
| Source Type | ESI | Ion Polarity | Positive | Set Nebulizer | 2.0 Bar |
| Focus | Active | Set Capillary | 3500 V | Set Dry Heater | 250 °C |
| Scan Begin | 150 m/z | Set End Plate Offset | -400 V | Set Dry Gas | 8.0 l/min |
| Scan End | 1000 m/z | Set Charging Voltage | 2000 V | Set Divert Valve | Waste |
| | | Set Corona | 4000 nA | Set APCI Heater | 200 °C |

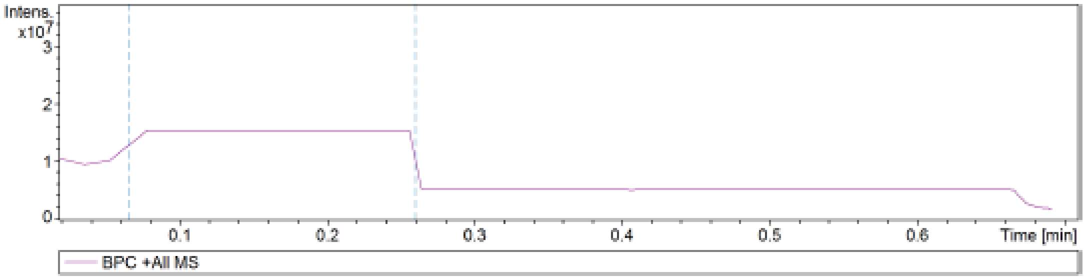


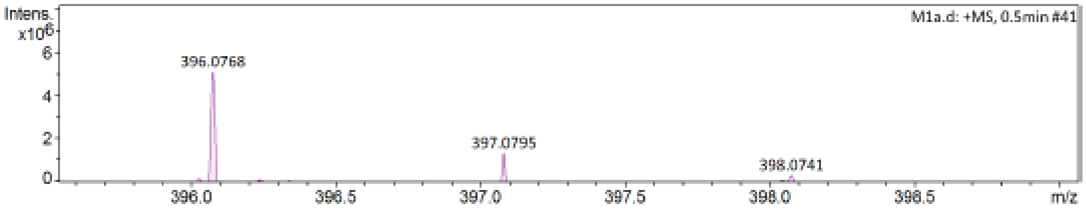


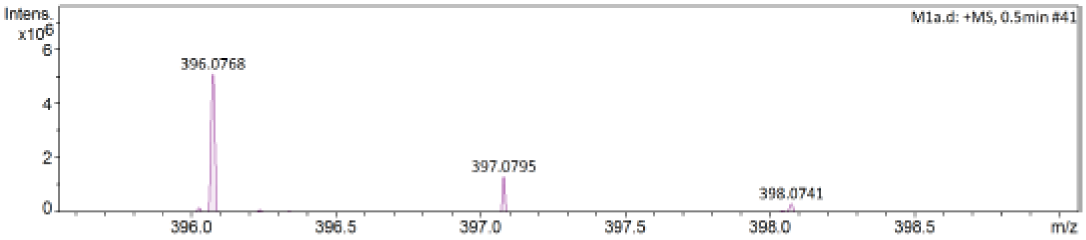


M1a.d

Bruker Compass DataAnalysis 4.4 printed: 8/22/2021 6:34:30 PM by: demo Page 1 of 1

Compound (*R*)-**16c**

# Display Report

**Analysis Info**

| | | | |
|---|---|---|---|
| Analysis Name | D:\Data\RawData\Azamat\KTH\Batch_2_final\M20.d | Acquisition Date | 8/22/2021 12:44:11 PM |
| Method | MS_fullscan_positive.m | Operator | Demo User |
| Sample Name | NaFormate | Instrument | impact II 1825265.10235 |
| Comment | | | |

**Acquisition Parameter**

| | | | | | |
|---|---|---|---|---|---|
| Source Type | ESI | Ion Polarity | Positive | Set Nebulizer | 2.0 Bar |
| Focus | Active | Set Capillary | 3500 V | Set Dry Heater | 250 °C |
| Scan Begin | 150 m/z | Set End Plate Offset | -400 V | Set Dry Gas | 8.0 l/min |
| Scan End | 1000 m/z | Set Charging Voltage | 2000 V | Set Divert Valve | Waste |
| | | Set Corona | 4000 nA | Set APCI Heater | 200 °C |

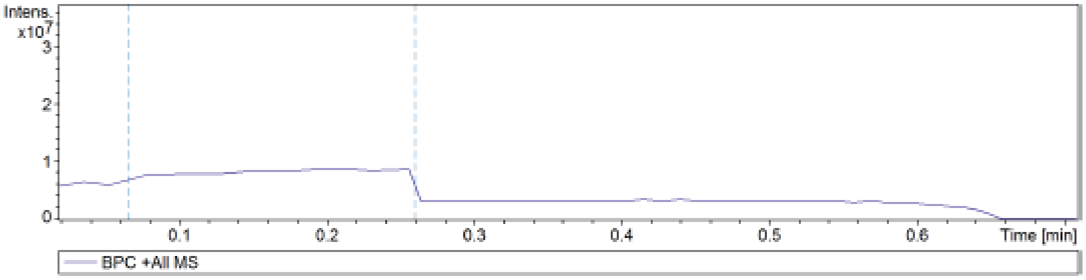


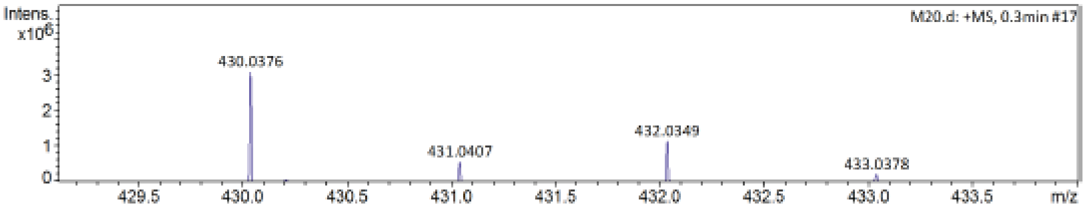


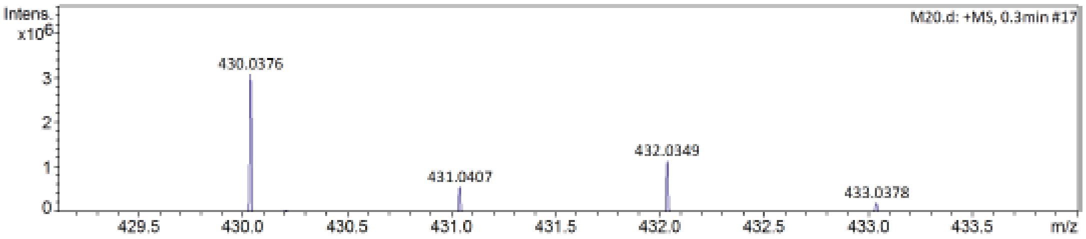


M20.d

Bruker Compass DataAnalysis 4.4 printed: 8/22/2021 6:38:00 PM by: demo Page 1 of 1

Compound **17**

# Display Report

**Analysis Info**

| | | | |
|---|---|---|---|
| Analysis Name | D:\Data\RawData\Azamat\KTH\Batch_2_final\M22.d | Acquisition Date | 8/22/2021 12:29:10 PM |
| Method | MS_fullscan_positive.m | Operator | Demo User |
| Sample Name | NaFormate | Instrument | impact II 1825265.10235 |
| Comment | | | |

**Acquisition Parameter**

| | | | | | |
|---|---|---|---|---|---|
| Source Type | ESI | Ion Polarity | Positive | Set Nebulizer | 2.0 Bar |
| Focus | Active | Set Capillary | 3500 V | Set Dry Heater | 250 °C |
| Scan Begin | 150 m/z | Set End Plate Offset | -400 V | Set Dry Gas | 8.0 l/min |
| Scan End | 1000 m/z | Set Charging Voltage | 2000 V | Set Divert Valve | Waste |
| | | Set Corona | 4000 nA | Set APCI Heater | 200 °C |

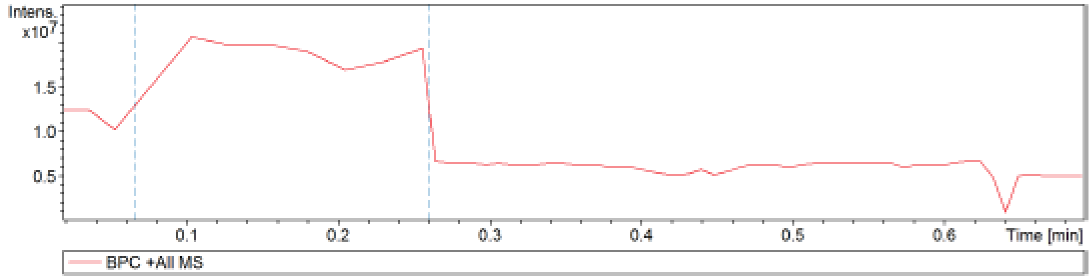


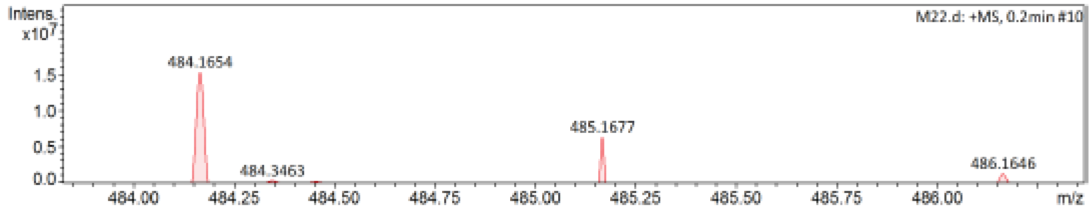


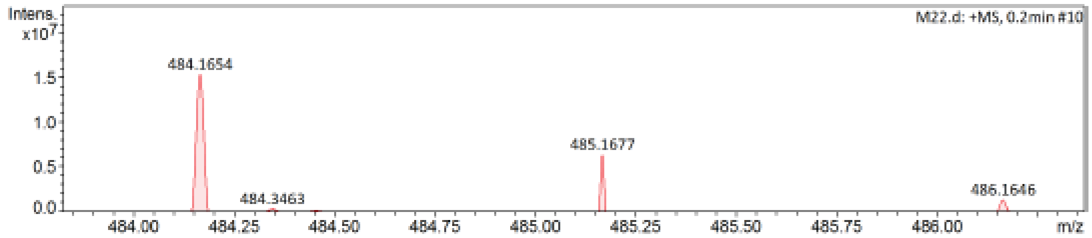

Compound **18**

# Display Report

**Analysis Info**

| | | | |
|---|---|---|---|
| Analysis Name | D:\Data\RawData\Azamat\KTH\Batch_2_final\M21.d | Acquisition Date | 8/22/2021 12:59:57 PM |
| Method | MS_fullscan_positive.m | Operator | Demo User |
| Sample Name | NaFormate | Instrument | impact II 1825265.10235 |
| Comment | | | |

**Acquisition Parameter**

| | | | | | |
|---|---|---|---|---|---|
| Source Type | ESI | Ion Polarity | Positive | Set Nebulizer | 2.0 Bar |
| Focus | Active | Set Capillary | 3500 V | Set Dry Heater | 250 °C |
| Scan Begin | 150 m/z | Set End Plate Offset | -400 V | Set Dry Gas | 8.0 l/min |
| Scan End | 1000 m/z | Set Charging Voltage | 2000 V | Set Divert Valve | Waste |
| | | Set Corona | 4000 nA | Set APCI Heater | 200 °C |

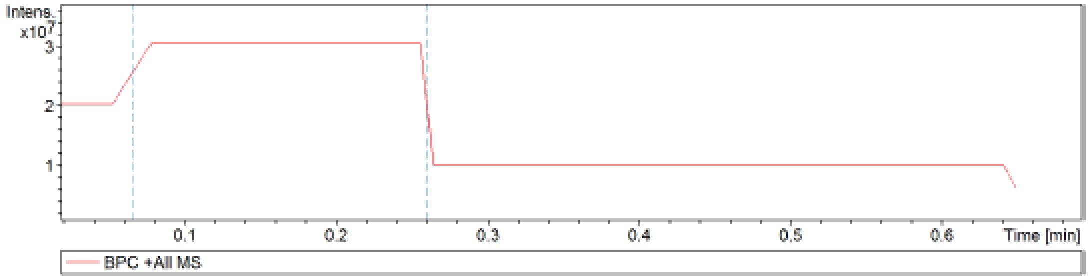


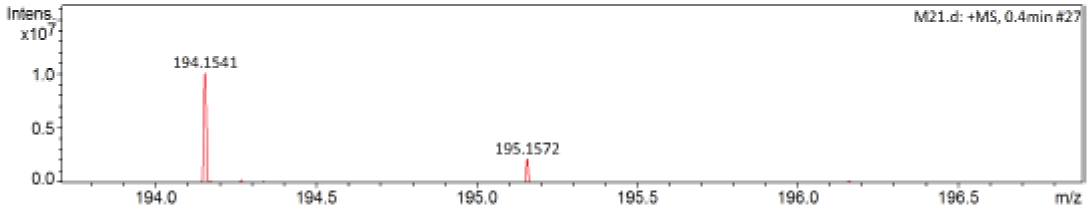


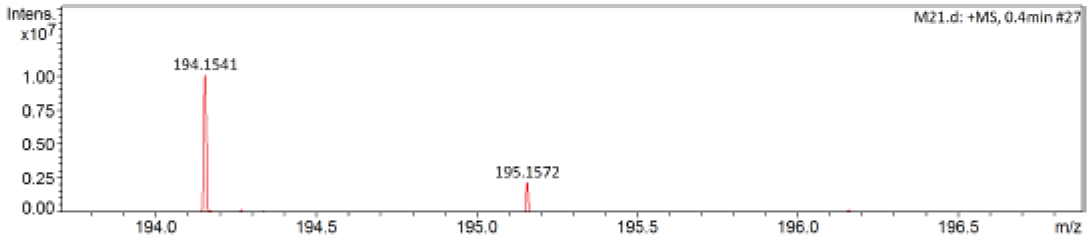

Supplement: Supplementary file 1 — jo1c02241_si_001.pdf [file jo1c02241_si_001.pdf]
